# Reference values of EORTC QLQ-C30, EORTC QLQ-BR23, and EQ-5D-5L for women with non-metastatic breast cancer at diagnosis and 2 years after

**DOI:** 10.1007/s11136-022-03327-4

**Published:** 2023-01-11

**Authors:** Carme Miret, Miren Orive, Maria Sala, Susana García-Gutiérrez, Cristina Sarasqueta, Maria Jose Legarreta, Maximino Redondo, Amado Rivero, Xavier Castells, José M. Quintana, Olatz Garin, Montse Ferrer, the REDISSEC-CaMISS Group, Mercè Comas, Laia Domingo, Francesc Macià, Marta Roman, Anabel Romero, Teresa Barata, Isabel Diez de la Lastra, Mariola de la Vega, Marisa Bare, Núria Torà, Joana Ferrer, Francesc Castanyer, Carmen Carmona, Susana García, Maximina Martín, Nerea Gonzalez, Maria Amparo Valverde, Alberto Saez, Inma Barredo, Manuel de Toro, Josefa Ferreiro, Jeanette Pérez, Cristina Valcárcel, María del Carmen Padilla, Teresa Téllez, Irene Zarcos, Cristina Churruca, Amaia Perales, Javier Recio, Irune Ruiz, Jose María Urraca, MªJesús Michelena, Julio Moreno, Gaizka Mallabiabarrena, Patricia Cobos, Borja Otero, Javier Gorostiaga, Itsaso Troya

**Affiliations:** 1 Department of Pediatrics, Obstetrics and Gynecology, Preventive Medicine and Public Health, Universitat Autònoma de Barcelona (UAB), 08193 Bellaterra, Barcelona, Spain; 2 Preventive Medicine and Public Health Training Unit PSMar-UPF-ASPB, Parc de Salut Mar, Agència de Salut Pública de Barcelona, i Universitat Pompeu Fabra, Barcelona, Spain; 3 IMIM (Hospital del Mar Medical Research Institute), Barcelona, Spain; 4 Departamento Psicología Social, Facultad Farmacia, UPV/EHU, Vitoria-Gasteiz, Araba Spain; 5 Osakidetza Basque Health Service, Research Unit, Galdakao-Usansolo University Hospital, Galdakao, Bizkai Spain; 6 KRONIKGUNE-Institute for Health Service Research, Barakaldo, Bizkaia Spain; 7 Health Services Research on Chronic Patients Network (REDISSEC), Galdakao, Bizkaia Spain; 8 Red de Investigación en Cronicidad, Atención Primaria y Promoción de la Salud (RICAPPS), Madrid, Spain; 9 Biodonostia Health Research Institute, Donostia University Hospital, Donostia, Gipuzkoa Spain; 10 Research and Innovation Unit, Hospital Costa del Sol, Marbella, Spain; 11 Servicio de Evaluación y Planificación del Servicio Canario de la Salud (SESCS), Tenerife, Spain; 12 Health Services Research Group, IMIM (Hospital del Mar Medical Research Institute), Doctor Aiguader 88, 08003 Barcelona, Spain; 13 CIBER en Epidemiología y Salud Pública (CIBERESP), Madrid, Spain; 14 Departament de Medicina i Ciències de la Vida, Universitat Pompeu Fabra (UPF), Barcelona, Spain

**Keywords:** Health-related quality of life, Breast cancer, Reference values, EORTC, EQ-5D-5L

## Abstract

**Purpose:**

To obtain reference norms of EORTC QLQ-C30, EORTC QLQ-BR23, and EQ-5D-5L, based on a population of Spanish non-metastatic breast cancer patients at diagnosis and 2 years after, according to relevant demographic and clinical characteristics.

**Methods:**

Multicentric prospective cohort study including consecutive women aged ≥ 18 years with a diagnosis of incident non-metastatic breast cancer from April 2013 to May 2015. Health-related quality of life (HRQoL) questionnaires were administered between diagnosis and beginning the therapy, and 2 years after. HRQoL differences according to age, comorbidity and stage were tested with ANOVA or Chi Square test and multivariate linear regression models.

**Results:**

1276 patients were included, with a mean age of 58 years. Multivariate models of EORTC QLQ-C30 summary score and EQ-5D-5L index at diagnosis and at 2-year follow-up show the independent association of comorbidity and tumor stage with HRQoL. The standardized multivariate regression coefficient of EORTC QLQ-C30 summary score was lower (poorer HRQoL) for women with stage II and III than for those with stage 0 at diagnosis (− 0.11 and − 0.07, *p* < 0.05) and follow-up (− 0.15 and − 0.10, *p* < 0.01). The EQ-5D-5L index indicated poorer HRQoL for women with Charlson comorbidity index ≥ 2 than comorbidity 0 both at diagnosis (− 0.13, *p* < 0.001) and follow-up (− 0.18, *p* < 0.001). Therefore, we provided the reference norms at diagnosis and at the 2-year follow-up, stratified by age, comorbidity index, and tumor stage.

**Conclusion:**

These HRQoL reference norms can be useful to interpret the scores of women with non-metastatic breast cancer, comparing them with country-specific reference values for this population.

**Supplementary Information:**

The online version contains supplementary material available at 10.1007/s11136-022-03327-4.

## Plain English summary

Breast Cancer is a chronic disease, since it is an ongoing condition that can recur, requires medical treatment and negatively affects health-related quality of life. The difficulty in interpreting quality of life scores prevents their use, but reference norms can help interpreting them. This study provides reference norms of three widespread quality of life measuring instruments (EORTC QLQ-C30, EORTC QLQ-BR23, and EQ-5D-5L), based on non-metastatic breast cancer patients in Spain.

Health-related quality of life questionnaires were administered to 1276 women with breast cancer at diagnosis and 2 years after. Quality of life was worse for women diagnosed at higher stages of cancer, and for those with other medical conditions. For this reason, reference norms at diagnosis and at the 2-year follow-up were provided according to age, number of conditions, and cancer stage. These reference norms can be useful to interpret the quality of life scores of women with non-metastatic breast cancer.

## Background

Breast Cancer is a chronic disease [1] since it is an ongoing condition that can recur, requires medical treatment and affects negatively the health-related quality of life (HRQoL) [2], which therefore has become an important outcome in these patients [3–6].

HRQoL instruments are generic or specific according to their target population, and they can in turn be classified as psychometric profiles or econometric indexes according to their measurement model [7]. Generic instruments are applicable to any population, and are well suited for the comparison among diseases [5, 8, 9], while disease-specific instruments are more responsive in detecting changes over time and differences between groups. The EQ-5D has probably been the most widely used generic econometric instrument [8, 9], and the instruments developed by the European Organization for Research and Treatment of Cancer (EORTC) are the most frequently used cancer-specific ones [10]: the Quality of Life Questionnaire C-30 (EORTC QLQ-C30), common for all tumor locations [11], and the specific module for Breast cancer (EORTC QLQ-BR23) [12].

The difficulty in interpreting HRQoL scores has been identified as one of the main barriers to the widespread use of this type of outcomes [13]. A well-established approach to aid interpretation of scores has been to produce tables of normative data [11, 13, 14]. Reference norms of the EQ-5D-5L based on general population are currently available for several countries [15–19], but not for population with breast cancer. Reference values of EORTC QLQ-C30 and EORTC QLQ-BR23 based on breast cancer patients are available since 2008 [20], and updated for the EORTC QLQ-C30 in 2020 stratifying by age, breast cancer stage (early or metastatic), comorbid conditions, and performance status [21]. However, in this update norms were constructed by pooling samples of multinational randomized clinical trials without providing data at country level, despite the consistent differences between countries [14] and geographic areas [21].

Although there are general population-based reference norms of EORTC QLQ-C30 available at country level [22–25], there are patient-based reference norms for women with breast cancer and other breast diseases only for Germany [26].

The present study aimed to obtain reference norms of the EORTC QLQ-C30, the EORTC QLQ-BR23, and the EQ-5D-5L, based on a population of Spanish non-metastatic breast cancer patients at diagnosis and 2 years after, according to relevant demographic and clinical characteristics.

## Methods

### Study population and setting

We analyzed data of patients included in the CaMISS (Spanish abbreviation for Health Services Research in Breast Cancer) prospective observational study [27] including consecutive women aged 18 years or older diagnosed with incident breast cancer in one of the participant Spanish hospitals from April 2013 to May 2015. Exclusion criteria were: diagnosis of sarcoma, lymphoma or inflammatory carcinoma; breast cancer recurrence; terminal illness; or inability to respond to questionnaires for any reason.

Women were selected from the lists for surgery and other oncological treatments through revision of inclusion and exclusion criteria in their medical records. Eligible patients were contacted, informed, and invited to participate by phone, and their written informed consent was requested. Out of the 1629 eligible patients invited to participate in the CaMISS study, 1456 accepted (89%): 1176 from 6 hospitals in the Basque Country, 97 from 2 hospitals in the Canary Islands, 97 from one hospital in Catalonia, and 86 from one hospital in Andalusia. After excluding 75 patients with no tumor stage information, 18 in stage IV and 87 without HRQoL data at diagnosis, 1276 patients were included in the analyses. Participants were followed for 2 years (Median = 1.98 years; P25–P75 = 1.94–2.05), and their re-evaluation was performed from May 2015 to December 2017.

HRQoL questionnaires were administered before surgery or before beginning the neoadjuvant therapy, and 2 years after diagnosis. The first administration between diagnosis and treatment was performed during a hospital visit (43.3%) or through telephone interviews (56.7%). 2 years after diagnosis, HRQoL questionnaires were sent by post mail, with reminders at 2 weeks and at 2 months. In the interval among reminders, non-responders were also telephoned to remind them that a questionnaire had been sent and also to offer them the option of responding over the phone if they preferred (17.1% were administered through telephone interviews). There was one person responsible for data collection per hospital, who were trained for recruitment procedures, extraction of data from medical records and administration of HRQoL questionnaires.

The study was approved by the ethics committees of all participating hospitals and conducted according to the principles expressed in the 2000 revision of the Declaration of Helsinki.

### Study variables

Participants' age, date of breast cancer diagnosis, clinical TNM classification, and diagnosis of other diseases were collected from medical records at recruitment. The Charlson Comorbidity Index [28] was constructed with 17 items, scoring from 0 to 6 points. Information about the treatments performed and recurrences suffered during the follow-up were collected from medical records 2 years after diagnosis.

The following socio-demographic variables were self-reported: education level, occupation, social class, and marital status. Social class was based on the Spanish National Classification of Occupations 2011, using a neo-Weberian approach [29]: (I) Large employers (≥ 10 employees) and higher grade professionals or managers; (II) Small employers, lower grade professionals or managers, higher grade technicians, sports professionals and artists; (III) Intermediate occupations (white collar workers); (IV) Lower supervisory and lower technician occupations; (V) Skilled primary workers and semi-skilled workers; and VI) Unskilled workers.

### Health-related quality of life measures

HRQoL was assessed using the Spanish validated versions of EORTC QLQ-C30 [30, 31], EORTC QLQ-BR23 [12], and EQ-5D-5L [32].

The EORTC QLQ-C30 [31, 33] is comprised of 30 items that assess five functional scales, eight cancer symptoms, a financial difficulties scale, and a global health status scale. A single higher-order summary score was calculated, using 27 out of the 30 items (excluding global health status and financial impact) [34]. The EORTC QLQ-BR23 [12] comprises 23 items that assess four functional and four symptoms scales. Responses were transformed into scores ranging from 0 to 100, were higher scores indicate greater burden for symptom scales, but better results in functional scales and the EORTC QLQ-C30 summary score [34].

The EQ-5D-5L [32] comprises 5 questions to measure the mobility, self-care, usual activities, pain/discomfort, and anxiety/depression dimensions with a 5 response options Likert scale (from none to extreme problems). The EQ-5D-5L includes also a 6^th^ question to self-rate health (EQ-VAS) with a visual analogue scale ranging from 0 to 100 (best health state imaginable). Applying the Spanish social preferences [35], a health index was obtained ranging from 1 (perfect health) to negative values for those health states considered worse than death. These preference values were obtained using a standardized protocol, combining the techniques of time trade-off and discrete choice [36].

### Statistical analysis

The statistical power of the study was estimated as > 0.8, with an alpha risk of 0.05 (Type I error), to detect mean differences of 0.4 standard deviation on HRQoL scores for the comparison with the smallest sample size between women with breast cancer in stage 0 and stage III (*N* = 116 and 72, respectively). The study power was calculated with R package version 1.3–0 [37].

Characteristics of women who completed the HRQoL telephone interview at diagnosis and after 2 years were compared to women who did not complete the follow-up evaluation, using the Chi square test. HRQoL differences according to age, comorbidity and stage were tested with ANOVA and Tukey post hoc analyses or Chi Square test. Bar chart figures were created showing our results together with general population-based reference norms of the EQ-5D-5L for Spanish women [15], and of the EORTC QLQ-C30 for patients with non-metastatic breast cancer from European and Anglo-Saxon countries [21].

To identify the factors independently associated with HRQoL at diagnosis and 2 years after, linear regression models with the EORTC QLQ-C30 summary score were constructed, as well as censored linear regression models (Tobit) with the EQ-5D-5L index due to the right-skewed distribution. Marginal effects were obtained from the Tobit model as averaged individual marginal effects to restore the original range of the EQ-5D-5L index [38].

Reference norms were estimated for all patients with non-metastatic breast cancer, stratifying by the variables independently associated with HRQoL. We provide the percentage and standard error for the EQ-5D-5L dimensions. For the continuous scores of EQ-5D-5L, EORTC QLQ-C30 and BR-23 we provide the deciles, percentiles 5 and 95, mean, standard deviation (SD), and 95% confidence interval (95% CI). All analyses were performed with IBM SPSS Statistics, version 25.

## Results

### Sample characteristics at diagnosis

Table 1 shows socio-demographic and clinical characteristics of the sample, as well as treatment and recurrences observed during the 2-year follow-up. Mean age of the 1276 participants at diagnosis was 58 years (SD = 12), most were married or with a couple (67%), and with breast cancer diagnosed in stage I (52%) and II (30%). The treatments adjuvant to surgery most frequently applied were hormonotherapy (85%) and external radiotherapy (83%). The most frequent treatment combinations are: surgery with radiotherapy and hormonotherapy, which was applied to more than 40% of patients, followed by this combination in addition with chemotherapy (18%), and surgery with hormonotherapy in almost 10% of the patients. Patients with other treatments received concomitant medication for side effects or other diseases, such as granulocyte colony-stimulating factor (8 patients), methotrexate or zoledronic acid.

**Table 1:** Characteristics of women with non-metastatic breast cancer at diagnosis, treatment applied and recurrences detected during the 2-year follow-up

	All patients	Patients with HRQoL assessment at diagnosis and follow-up	Patients without HRQoL assessment at follow-up	*p* value*
N	1276	1108	168	
Socio-demographic				
Age (years)				
< 40	76 (6.0%)	57 (5.1%)	19 (11.3%)	0.007
40–65	878 (68.8%)	771 (69.6%)	107 (63.7%)	
> 65	322 (25.2%)	280 (25.3%)	42 (25.0%)	
Education level				
Primary school or less	317 (25.3%)	267 (24.6%)	50 (30.3%)	0.066
Middle school	332 (26.5%)	299 (27.5%)	33 (20.0%)	
High school	302 (24.1%)	267 (24.6%)	35 (21.2%)	
University and above	300 (24.0%)	253 (23.3%)	47 (28.5%)	
Occupation				
Working	582 (46.7%)	511 (47.2%)	71 (43.3%)	0.039
Housewife	236 (18.9%)	211 (19.5%)	25 (15.2%)	
Retired or unable to work	91 (7.3%)	70 (6.5%)	21 (12.8%)	
Unemployed	314 (25.2%)	271 (25.0%)	43 (26.2%)	
Others	23 (1.8%)	19 (1.8%)	4 (2.4%)	
Social class				
I–II	240 (22.9%)	205 (22.3%)	35 (27.6%)	0.156
III	232 (22.2%)	213 (23.2%)	19 (15.0%)	
IV	425 (40.6%)	373 (40.5%)	52 (40.9%)	
V–VI	150 (14.3%)	129 (14.0%)	21 (16.5%)	
* Missings*	*229*	*188*	*41*	
Marital status				
Single	133 (10.6%)	106 (9.7%)	27 (16.3%)	0.019
Married/couple	849 (67.4%)	750 (68.6%)	99 (59.6%)	
Widowed/divorced/separated	278 (22.1%)	238 (21.8%)	40 (24.1%)	
Clinical				
Screening detection				
No	598 (46.9%)	506 (45.7%)	92 (54.8%)	0.028
Yes	678 (53.1%)	602 (54.3%)	76 (45.2%)	
Tumor stage				
Stage 0	132 (10.3%)	116 (10.5%)	16 (9.5%)	0.058
Stage I	666 (52.2%)	588 (53.1%)	78 (46.4%)	
Stage II	386 (30.3%)	332 (30.0%)	54 (32.1%)	
Stage III	92 (7.2%)	72 (6.5%)	20 (11.9%)	
Charlson comorbidity index				
0	1017 (79.7%)	881 (79.5%)	136 (81.0%)	0.299
1	165 (12.9%)	143 (12.9%)	22 (13.1%)	
2	68 (5.3%)	63 (5.7%)	5 (3.0%)	
3	21 (1.6%)	16 (1.4%)	5 (3.0%)	
4 or more	5 (0.4%)	5 (0.5%)	0 (0.0%)	
Treatment type during 2 years follow-up				
Any neoadjuvant treatment	120 (9.4%)	98 (8.8%)	22 (13.1%)	0.079
Breast surgery				
Breast-conserving surgery	551 (43.2%)	490 (44.3%)	61 (36.3%)	0.001
Mastectomy	298 (23.4%)	239 (21.6%)	59 (35.1%)	
Missing	426 (33.4%)	378 (34.1%)	48 (28.6%)	
Lymphadenectomy	273 (21.4%)	227 (20.5%)	46 (27.4%)	0.042
Adjuvant treatment				
Chemotherapy	436 (34.3%)	379 (34.3%)	57 (34.3%)	0.944
External Radiotherapy	1059 (83.4%)	934 (84.6%)	125 (75.3%)	0.001
Brachytherapy	65 (5.1%)	56 (5.1%)	9 (5.4%)	0.868
Hormonotherapy	1081 (85.1%)	945 (85.5%)	136 (82.4%)	0.145
Anti-HER2	112 (8.8%)	95 (8.6%)	17 (10.3%)	0.510
Other	11 (0.9%)	9 (0.9%)	2 (1.3%)	0.607
Recurrences during 2 years follow-up				
Loco-regional	19 (1.5%)	11 (1.0%)	8 (4.8%)	< 0.001
Metastases	27 (2.1%)	14 (1.3%)	13 (7.7%)	< 0.001

*PRO* patient reported outcome

*Chi-Squared Test

Statistically significant differences were found between the 1108 patients who completed the two HRQoL assessments and the 168 who did not complete the second evaluation. The latter group were younger, more frequently without couple, underwent mastectomy and lymphadenectomy, and had a higher percentage of detected recurrences.

**Fig. 1:**
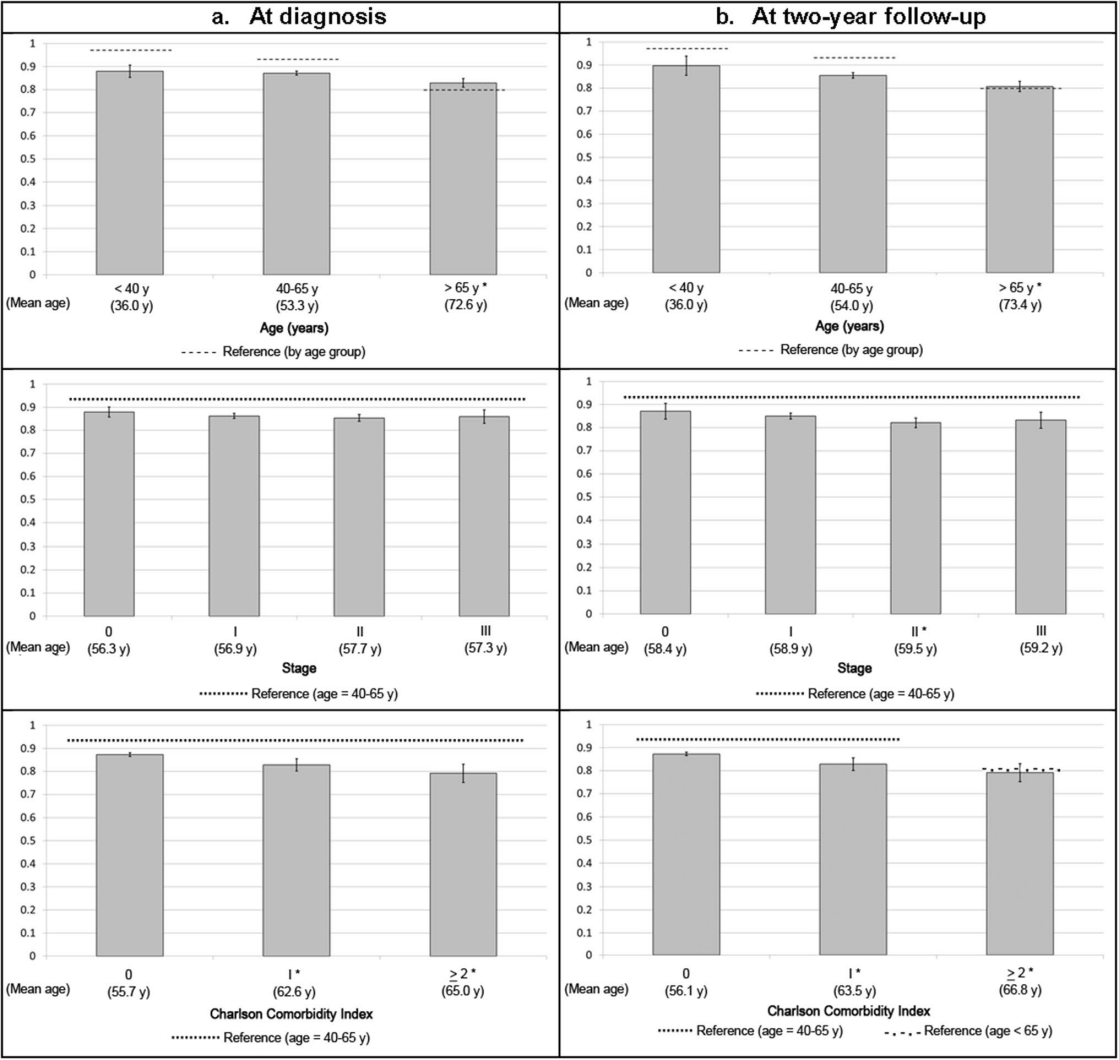
EQ-5D-5L Index according to age, tumor stage and Charlson Comorbidity Index: bars represent means and their 95% confidence intervals at diagnosis and at 2-year follow-up. Discontinuous lines represent the EQ-5D-5L index means of reference norms based on general population [15] Spanish women. Y axis represents the EQ-5D-5L index values from 1 (perfect health) to 0 (death). *p value < 0.05 obtained using Tukey post hoc analysis for comparing each category with the reference: aged < 40 years old, tumor stage 0, or Charlson Comorbidity Index 0

**Fig. 2:**
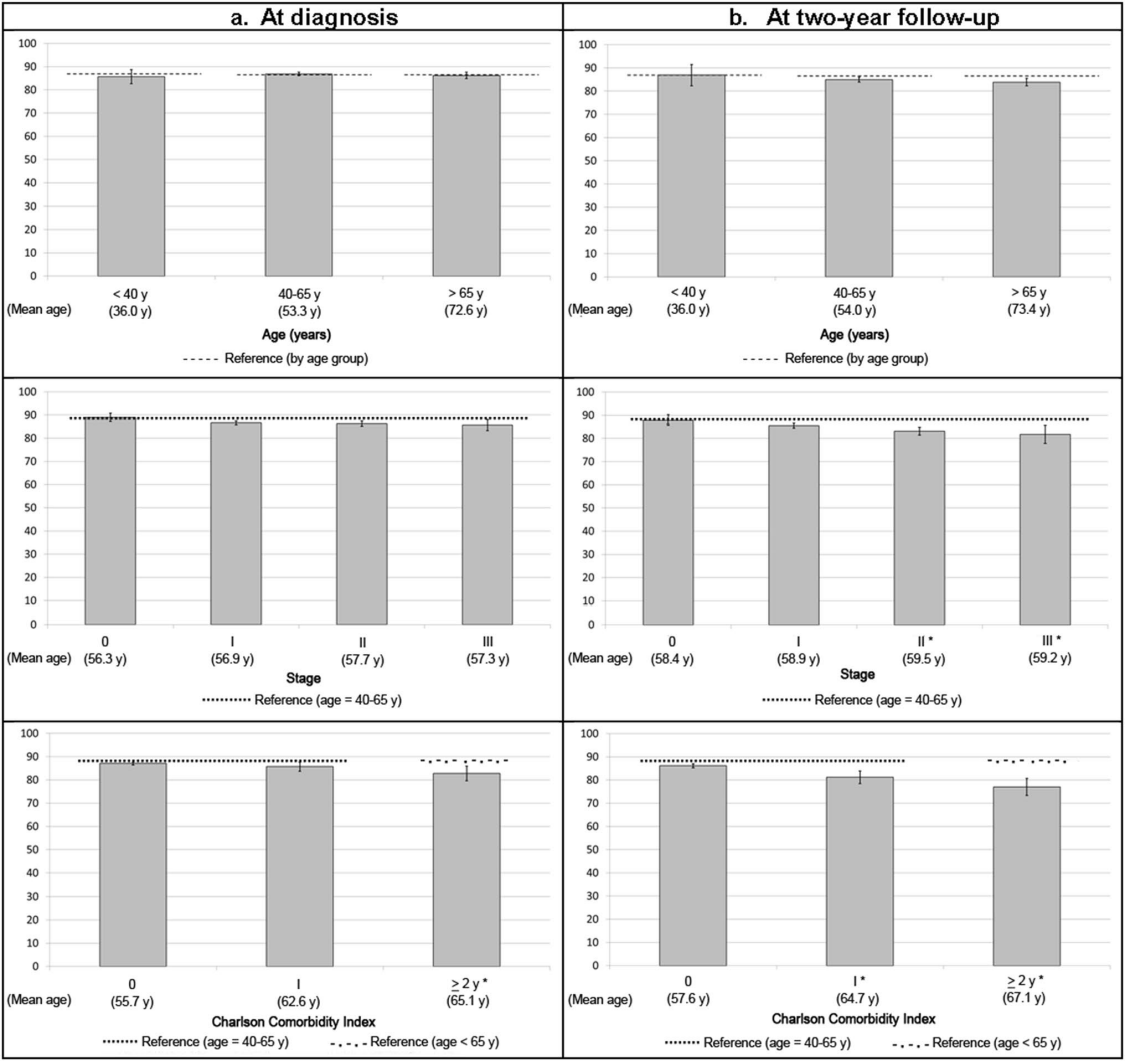
EORTC QLQ-C30 summary score according to age, tumor stage and Charlson Comorbidity Index: bars represent means and their 95% confidence intervals at diagnosis and at 2-year follow-up. Discontinuous lines represent the EORTC QLQ-C30 reference norms based on patients with non-metastatic breast cancer at diagnosis [21]. Y axis represents the EORTC QLQ-C30 summary score from 0 (worst) to 100 (best results). *p value < 0.05 obtained using Tukey post hoc analysis for comparing each category with the reference: aged < 40 years old, tumor stage 0, or Charlson Comorbidity Index 0

Figure 1 shows that EQ-5D-5L index means for breast cancer patients were significantly lower (worse) than the reference norms based on general population of Spanish women [15] at the two time points, except for patients aged > 65 years. In general, the EORTC QLQ-C30 summary score means for breast cancer patients at diagnosis (Fig. 2) were similar to the reference norms based on European and Anglo-Saxon patients [21] at diagnosis, but significantly lower (worse) at the 2-year follow-up.

Table 2 shows statistically significant differences on EQ-5D-5L by age group and comorbidity, both at diagnosis and 2 years after. Only usual activities and pain/discomfort EQ-5D dimensions presented differences by tumor stage at diagnosis; while all the dimensions, the index and the EQ-VAS presented statistically significant differences after 2 years. Similarly, more EORTC QLQ-C30 scales (Table 3) presented statistically significant differences by comorbidity index and tumor stage at 2 years of follow-up than at diagnosis. Some scores of the EORTC BR23 (Table 4) also presented significant differences among groups defined by these variables.

**Table 2:** EQ-5D-5L results according to age, Charlson Comorbidity Index and stage: frequency and percentage of patients that reported having any problem in each dimension and mean (SD) of EQ-5D-5L index* and EQ-VAS**

	Mobility	Self-care	Activity	Pain	Anxiety	EQ-5D-5L index	EQ-VAS
AT diagnosis							
All	146 (11.5%)	59 (4.6%)	162 (12.7%)	468 (36.8%)	811 (63.8%)	0.86 (0.14)	73.1 (19.9)
Age							
< 40	3 (3.9%)	3 (3.9%)	13 (17.1%)	30 (40.0%)	43 (57.3%)	0.90 (0.10)	78.0 (20.8)
40–65	58 (6.6%)	29 (3.3%)	85 (9.7%)	306 (34.9%)	557 (63.4%)	0.87 (0.13)	73.5 (20.0)
> 65	85 (26.6%)	27 (8.4%)	64 (20.0%)	132 (41.3%)	211 (66.1%)	0.84 (0.16)	72.3 (19.9)
*p* value	< 0.001	0.001	< 0.001	0.106	0.339	< .001	.259
Comorbidity index							
0	73 (7.2%)	35 (3.4%)	112 (11.0%)	356 (35.1%)	638 (62.9%)	0.87 (0.13)	74.6 (18.9)
≥ 1	73 (28.3%)	24 (9.4%)	50 (19.4%)	112 (43.4%)	173 (67.1%)	0.82 (0.18)	67.4 (22.5)
*p* value	< 0.001	< 0.001	< 0.001	0.013	0.217	< .001	< .001
Tumor stage							
0	12 (9.1%)	7 (5.3%)	13 (9.8%)	45 (34.1%)	81 (61.4%)	0.88 (0.12)	75.5 (18.8)
I	66 (9.9%)	28 (4.2%)	72 (10.8%)	229 (34.4%)	434 (65.2%)	0.86 (0.14)	73.8 418.9)
II	55 (14.3%)	19 (4.9%)	61 (15.9%)	164 (42.7%)	238 (62.0%)	0.85 (0.15)	72.1 (21.2)
III	13 (14.1%)	5 (5.4%)	16 (17.4%)	30 (33.0%)	58 (64.4%)	0.86 (0.14)	69.5 (22.7)
*p* value	0.109	0.888	0.038	0.038	0.696	.341	.085
At 2-year follow-up							
All	302 (26.5%)	117 (10.3%)	359 (31.5%)	639 (56.3%)	502 (44.2%)	0.84 (0.18)	74.5 (18.4)
Age							
< 40 years	2 (5.6%)	2 (5.6%)	7 (19.4%)	16 (44.4%)	16 (44.4%)	0.90 (0.12)	79.9 (13.6)
40–65 years	168 (22.1%)	57 (7.5%)	223 (29.4%)	421 (55.4%)	334 (44.1%)	0.85 (0.16)	76.1 (17.8)
> 65 years	131 (38.9%)	57 (17.0%)	128 (37.9%)	201 (60.2%)	152 (45.2%)	0.81 (0.21)	70.2 (19.6)
*p* value	< 0.001	< 0.001	0.006	0.114	0.937	< .001	< .001
Comorbidity index							
0	191 (21.2%)	67 (7.4%)	251 (27.8%)	479 (53.2%)	383 (42.6%)	0.86 (0.16)	76.4 (17.4)
≥ 1	111 (46.8%)	50 (21.2%)	108 (45.6%)	160 (68.1%)	119 (50.2%)	0.77 (0.23)	67.1 (20.3)
*p* value	0.278	< 0.001	0.001	0.007	0.155	< .001	< .001
Tumor stage							
0	23 (19.7%)	5 (4.3%)	20 (17.2%)	56 (47.9%)	41 (34.5%)	0.87 (0.19)	78.6 (16.6)
I	161 (26.4%)	51 (8.4%)	186 (30.5%)	326 (53.6%)	274 (45.2%)	0.85 (0.17)	74.9 (18.5)
II	97 (28.8%)	53 (15.8%)	124 (36.6%)	208 (61.7%)	152 (45.1%)	0.82 (0.19)	73.2 (19.0)
III	21 (28.4%)	8 (10.8%)	29 (39.2%)	49 (66.2%)	35 (47.3%)	0.83 (0.15)	69.9 (17.4)
*p* value	< 0.001	< 0.001	< 0.001	< 0.001	0.036	.027	.006

The five-level response scale of EQ-5D-5L dimensions was dichotomized into “no problems” versus “any problem” (slight, moderate, severe, or extreme problems), and percentages were compared with Chi Square test

^*^ EQ-5D-5L index ranged from 1 (perfect health) to negative values for those health states considered worse than death. ** EQ-VAS ranged from 0 to 100 (best state imaginable health). Differences on mean scores of the EQ-5D-5L index and EQVAS were tested with ANOVA test

**Table 3:** Mean scores and standard deviations of EORTC QLQ-C30 according to age, Charlson Comorbidity Index, and tumor stage

	All	Age				Comorbidity index			Tumor stage				
		< 40	40–65	> 65	*p* value*	0	≥ 1	*p* value*	0	I	II	III	*p* value*
At diagnosis													
Summary score	86.6 (11.5)	85.7 (13.0)	86.9 (11.2)	86.2 (12.2)	.533	87.1 (10.9)	84.6 (13.6)	.002	89.0 (10.3)	86.6 (11.7)	86.2 (11.7)	85.7 (11.6)	.085
Physical function	92.6 (13.6)	96.9 (7.0)	94.5 (11.4)	86.6 (17.8)	< .001	94.3 (11.2)	86.0 (19.0)	< .001	93.9 (12.9)	93.4 (12.6)	91.1 (15.2)	91.9 (14.0)	.045
Role function	91.4 (19.7)	89.5 (21.7)	91.7 (19.1)	91.0 (20.9)	.589	91.7 (18.9)	90.4 (22.7)	.365	92.2 (17.4)	91.9 (19.2)	90.8 (20.8)	89.7 (21.7)	.643
Emotional function	65.7 (23.3)	60.0 (22.6)	65.5 (23.3)	67.4 (23.5)	.043	65.9 (23.0)	64.7 (24.7)	.442	70.5 (22.2)	64.9 (23.7)	66.8 (22.2)	59.6 (25.0)	.003
Cognitive function	85.6 (20.3)	86.0 (17.4)	85.3 (20.7)	86.2 (20.0)	.782	85.9 (19.9)	84.3 (21.9)	.265	85.8 (20.6)	85.4 (20.1)	86.4 (20.3)	83.2 (21.6)	.559
Social function	89.0 (21.0)	81.8 (27.4)	88.4 (21.2)	92.4 (17.7)	< .001	89.1 (20.8)	88.4 (21.6)	.640	90.7 (19.7)	89.7 (19.9)	87.7 (22.5)	87.0 (23.0)	.282
Global health status	73.5 (19.6)	72.8 (18.9)	73.9 (19.3)	72.6 (20.8)	.561	74.7 (18.7)	68.7 (22.4)	< .001	75.0 (19.0)	74.2 (19.2)	72.1 (19.9)	71.9 (21.8)	.263
Fatigue	15.9 (19.8)	16.1 (21.3)	15.0 (19.3)	18.2 (20.5)	.041	14.8 (18.6)	19.9 (23.6)	< .001	13.2 (17.8)	15.1 (19.1)	17.7 (21.1)	17.3 (20.8)	.066
Nausea	2.7 (9.9)	2.9 (9.6)	2.9 (10.2)	1.9 (8.9)	.257	2.6 (9.4)	2.9 (11.5)	.722	2.6 (9.4)	2.5 (9.3)	2.9 (11.0)	2.9 (9.8)	.926
Pain	13.0 (20.6)	13.6 (20.7)	11.7 (19.4)	16.2 (23.3)	.003	12.3 (19.7)	15.6 (23.6)	.020	11.0 (18.4)	12.4 (20.2)	14.8 (22.0)	11.8 (20.3)	.161
Dyspnea	9.4 (19.9)	6.1 (14.1)	9.5 (20.1)	10.0 (20.7)	.311	9.3 (19.6)	10.0 (21.2)	.609	8.1 (18.5)	9.7 (20.2)	9.3 (20.3)	9.4 (18.7)	.871
Insomnia	32.0 (31.1)	29.4 (31.7)	32.5 (30.9)	31.2 (31.5)	.608	31.7 (30.5)	33.2 (33.3)	.475	30.3 (31.2)	33.0 (31.1)	30.6 (30.4)	32.6 (33.1)	.575
Appetite loss	12.7 (22.5)	17.8 (25.3)	12.9 (22.7)	11.1 (21.2)	.065	12.3 (21.8)	14.2 (24.8)	.247	9.3 (20.3)	12.4 (22.0)	14.4 (24.0)	12.3 (22.5)	.147
Constipation	9.8 (21.2)	7.5 (18.5)	9.0 (20.3)	12.6 (23.7)	.023	9.1 (20.0)	12.6 (25.0)	.019	8.7 (17.9)	9.7 (21.5)	10.9 (22.1)	8.1 (18.8)	.567
Diarrhea	5.0 (14.7)	4.8 (16.1)	5.2 (14.6)	4.4 (14.6)	.753	4.6 (13.5)	6.4 (18.5)	.075	2.6 (10.7)	5.2 (14.9)	4.7 (14.3)	7.7 (18.6)	.075
Financial difficulties	6.2 (18.1)	10.1 (24.4)	6.4 (18.1)	4.5 (16.0)	.039	5.8 (17.3)	7.5 (20.7)	.187	5.4 (15.4)	6.6 (18.9)	6.1 (18.0)	4.4 (15.2)	.681
At 2-year follow-up													
Summary score	84.7 (14.6)	86.9 (13.7)	84.9 (14.7)	83.9 (14.6)	.368	86.1 (13.5)	79.6 (17.2)	< .001	87.9 (12.5)	85.5 (14.0)	83.0 (15.6)	81.8 (16.4)	.002
Physical function	86.3 (17.5)	93.0 (10.4)	88.4 (15.0)	81.0 (21.6)	< .001	88.6 (15.0)	78.0 (22.9)	< .001	91.6 (12.8)	87.5 (16.6)	82.9 (20.2)	83.3 (14.6)	< .001
Role function	85.8 (23.6)	88.4 (20.6)	87.2 (22.0)	82.1 (27.0)	.004	87.7 (21.8)	78.5 (28.4)	< .001	89.4 (19.9)	87.2 (22.2)	82.6 (26.3)	81.9 (25.6)	.004
Emotional function	78.1 (22.6)	77.8 (24.4)	77.6 (23.3)	78.9 (20.9)	.714	78.9 (22.1)	75.1 (24.4)	.020	81.1 (21.4)	77.8 (22.5)	78.0 (22.6)	76.0 (25.0)	.427
Cognitive function	84.0 (22.2)	87.0 (22.9)	83.8 (22.9)	84.0 (20.6)	.685	84.8 (21.8)	80.8 (23.7)	.012	85.7 (21.1)	84.3 (21.8)	83.7 (22.6)	80.0 (25.7)	.346
Social function	85.7 (23.8)	84.7 (19.3)	84.7 (24.5)	88.0 (22.6)	.106	87.0 (22.6)	80.8 (27.5)	< .001	91.7 (17.9)	87.0 (21.9)	82.1 (26.6)	82.2 (30.0)	< .001
Global health status	72.3 (20.6)	76.4 (17.3)	73.3 (20.2)	69.4 (21.8)	.008	74.4 (19.6)	64.4 (22.4)	< .001	74.6 (20.1)	73.4 (19.6)	69.7 (22.6)	71.1 (19.8)	.031
Fatigue	25.4 (24.5)	21.8 (19.8)	24.9 (24.5)	27.3 (24.9)	.211	23.2 (23.3)	33.9 (26.8)	< .001	21.6 (23.4)	24.9 (24.2)	27.5 (25.1)	26.8 (25.1)	.114
Nausea	3.4 (11.0)	1.4 (4.7)	3.4 (10.6)	3.6 (12.5)	.535	2.9 (9.6)	5.1 (15.1)	.006	3.6 (11.1)	2.7 (9.7)	4.0 (11.9)	5.6 (15.9)	.090
Pain	21.4 (24.8)	18.1 (23.4)	21.3 (24.7)	22.4 (25.2)	.567	19.7 (23.5)	28.2 (28.2)	< .001	17.5 (24.4)	20.0 (23.8)	24.9 (26.0)	24.1 (25.8)	.006
Dyspnea	11.4 (21.4)	12.0 (22.8)	12.2 (21.6)	9.8 (20.8)	.212	10.0 (19.7)	16.9 (26.0)	< .001	11.7 (20.1)	10.5 (20.0)	12.8 (23.7)	12.7 (22.8)	.429
Insomnia	31.4 (32.4)	26.9 (30.7)	32.3 (33.0)	30.1 (31.4)	.407	30.3 (31.6)	35.3 (35.0)	.035	26.6 (31.5)	31.3 (32.1)	31.7 (32.2)	38.4 (36.5)	.111
Appetite loss	8.3 (20.0)	2.8 (9.3)	7.6 (18.8)	10.7 (23.2)	.017	7.0 (17.8)	13.3 (26.3)	< .001	4.4 (12.2)	7.5 (18.7)	10.3 (22.4)	12.7 (27.2)	.007
Constipation	14.6 (24.9)	13.9 (24.4)	13.5 (24.1)	17.2 (26.6)	.077	13.6 (24.1)	18.3 (27.5)	.010	13.7 (23.5)	14.4 (24.6)	15.2 (26.2)	14.0 (23.4)	.932
Diarrhea	6.0 (16.2)	4.6 (14.1)	5.0 (14.5)	8.5 (19.7)	.003	5.1 (14.4)	9.1 (21.4)	.001	4.5 (13.0)	5.7 (15.6)	6.6 (17.0)	7.7 (21.0)	.470
Financial difficulties	11.6 (24.1)	18.5 (30.3)	13.2 (25.4)	7.4 (19.4)	< .001	11.4 (23.7)	12.2 (25.4)	.648	7.8 (19.7)	10.8 (23.7)	13.9 (26.0)	12.8 (24.0)	.073

*EORTC QLQ-C30 differences according to age, comorbidity and stage were tested with ANOVA

**Table 4:** Mean scores and standard deviations of EORTC QLQ-BR-23 according to age, Charlson Comorbidity Index, and stage

	All	Age				Comorbidity index			Tumor stage				
		< 40	40–65	> 65	*p* value*	0	≥ 1	*p* value*	0	I	II	III	*p* value*
**At diagnosis**													
Body image	92.3 (16.7)	87.5 (20.8)	92.0 (17.0)	94.5 (14.4)	.003	92.4 (16.3)	92.1 (18.4)	.779	94.2 (12.6)	92.6 (16.7)	91.9 (17.2)	89.8 (19.8)	.258
Sexual function	23.5 (26.1)	31.3 (27.1)	27.4 (26.6)	10.6 (19.2)	< .001	24.9 (25.8)	18.2 (26.5)	< .001	24.7 (26.5)	24.9 (26.1)	21.1 (25.4)	21.8 (28.3)	.126
Sexual enjoy	54.6 (29.1)	59.4 (32.1)	56.2 (28.8)	42.5 (26.1)	< .001	55.3 (28.9)	50.5 (29.9)	.140	54.5 (26.6)	54.8 (28.3)	53.6 (29.7)	57 (36.0)	.913
Future perspective	45.9 (31.9)	36.4 (32.0)	45.7 (31.7)	48.8 (32.2)	.009	45.8 (31.3)	46.3 (34.2)	.822	50.3 (29.9)	45.8 (32.1)	46.4 (32.4)	39.2 (31.3)	.090
Systemic therapy effects	12.5 (13.9)	11.5 (13.9)	12.7 (14.4)	12.4 (12.7)	.764	12.2 (13.8)	13.6 (14.3)	.169	12.6 (13.5)	11.8 (13.2)	13.2 (14.8)	14.4 (16.2)	.235
Breast symptoms	13.2 (16.9)	19.2 (20.4)	13.4 (16.9)	11.1 (15.8)	.001	13.7 (17.2)	11.0 (15.6)	.021	13.4 (17.2)	12.5 (17.2)	14.4 (16.7)	12.9 (15.8)	.342
Arm symptoms	8.7 (15.6)	6.4 (12.5)	8.4 (14.7)	10.0 (18.3)	.127	8.2 (14.7)	11.0 (18.5)	.011	7.8 (14.0)	8.4 (15.5)	9.6 (15.8)	8.6 (17.2)	.592
Upset hair loss	24.0 (31.6)	12.3 (27.7)	25.6 (33.2)	23.6 (27.7)	.223	23.5 (31.4)	26.7 (33.1)	.564	11.1 (18.5)	22.9 (32.8)	28.6 (32.5)	33.3 (31.2)	.053
**At 2-year follow-up**													
Body image	84.6 (24.6)	79.7 (28.6)	82.9 (25.8)	88.8 (21.0)	.001	84.6 (24.7)	84.7 (24.4)	.956	90.1 (18.3)	86.7 (22.4)	81.4 (27.0)	73.5 (33.7)	< .001
Sexual function	20.9 (23.5)	29.5 (21.0)	25 (24.1)	9.9 (17.9)	< .001	22.4 (24.0)	15.4 (21.0)	< .001	23.8 (24.1)	21.7 (24.3)	18.9 (22.5)	19 (19.8)	.163
Sexual enjoy	50.3 (27.8)	62.8 (23.7)	53.2 (27.0)	34.3 (27.1)	< .001	51.3 (28.1)	45.6 (26.2)	.070	52.8 (26.3)	51.3 (29.2)	48.2 (27.1)	47 (22.6)	.518
Future perspective	59.5 (32.1)	55.9 (37.4)	57.1 (31.9)	65.0 (31.4)	.001	59.2 (31.9)	60.6 (33.0)	.557	66.4 (29.3)	59.6 (31.6)	59.1 (32.4)	48.4 (36.9)	.003
Systemic therapy effects	18.3 (16.5)	12.2 (11.1)	18.9 (16.8)	17.9 (16.1)	.056	17.7 (16.3)	20.7 (17.3)	.014	16.6 (15.4)	17.8 (15.8)	19.3 (17.8)	21.1 (17.8)	.171
Breast symptoms	16.0 (17.8)	17.4 (19.5)	17.4 (18.0)	13.0 (16.9)	.001	15.8 (16.8)	16.6 (21.0)	.572	15.0 (18.5)	16.0 (17.0)	16.1 (18.7)	17.2 (18.5)	.859
Arm symptoms	15.7 (20.3)	9.5 (15.0)	16.2 (20.3)	15.5 (20.8)	.157	15.0 (19.5)	18.2 (23.0)	.035	12.0 (18.0)	14.2 (19.4)	18.2 (21.0)	22.8 (24.8)	< .001
Upset hair loss	31.1 (33.7)	13.9 (30.0)	32.0 (33.4)	31.2 (34.3)	.192	29.4 (32.6)	36.9 (36.7)	.058	22.0 (26.8)	29.8 (33.4)	34.4 (34.5)	42.0 (40.5)	.066

^*^EORTC QLQ-BR-23 differences according to age, comorbidity and stage were tested with ANOVA

Multivariate models of EQ-5D-5L iIndex and EORTC QLQ-C30 summary score at diagnosis and at 2-year follow-up (Table 5) show the independent association of comorbidity and tumor stage with HRQoL. The standardized multivariate regression coefficient of EORTC QLQ-C30 summary score indicates that HRQoL was poorer for women with stage II and III than for those with stage 0 both at diagnosis (− 0.11 and − 0.07, *p* < 0.05) and at follow-up (− 0.15 and − 0.10, *p* < 0.01). The EQ-5D-5L index also indicated poorer HRQoL for women with Charlson comorbidity index ≥ 2 than comorbidity 0 at diagnosis (− 0.13, *p* < 0.001) and at follow-up (− 0.18, *p* < 0.001).

**Table 5:** Multivariate models of the EQ-5D-5L index and EORTC QLQ-C30 summary score as dependent variables

	At diagnosis				At 2-year follow-up			
	Coefficient (SE)	95% CI	Standardized Coefficient	*p* value	Coefficient (SE)	95% CI	Standardized Coefficient	*p* value
EQ-5D-5L index								
Intercept	0.90 (0.02)	[0.86, 0.93]		< .001	0.93 (0.03)	[0.86, 0.99]		< 0.001
Age								
< 40 years	Ref				Ref			
40–65 years	− 0.01 (0.02)	[− 0.04, 0.03]	− 0.02	.774	− 0.03 (0.02)	[− 0.07, 0.02]	− 0.07	0.231
> 65 years	− 0.03 (0.02)	[− 0.07, 0.00]	− 0.11	.055	− 0.06 (0.02)	[− 0.11, -0.02]	− 0.15	0.010
Comorbidity index								
0	Ref				Ref			
1	− 0.04 (0.01)	[− 0.06, -0.01]	− 0.09	.002	− 0.07 (0.02)	[− 0.10, -0.04]	− 0.13	< 0.001
≥ 2	− 0.07 (0.02)	[− 0.10, -0.04]	− 0.13	< .001	− 0.12 (0.02)	[− 0.16, -0.08]	− 0.18	< 0.001
Tumor stage								
0	Ref				Ref			
I	− 0.01 (0.01)	[− 0.04, 0.02]	− 0.04	.415	− 0.01 (0.02)	[− 0.05, 0.02]	− 0.03	0.513
II	− 0.02 (0.01)	[− 0.05, 0.01]	− 0.06	.186	− 0.04 (0.02)	[− 0.08, -0.00]	− 0.11	0.026
III	− 0.01 (0.02)	[− 0.05, 0.03]	− 0.02	.545	− 0.03 (0.03)	[− 0.08, 0.02]	− 0.04	0.232
EORTC QLQ-C30 summary score								
Intercept	88.12 (1.66)	[84.87, 91.36]		< .001	90.66 (2.69)	[85.38, 95.93]		< .001
Age								
< 40	Ref				Ref			
40–65	1.28 (1.39)	[− 1.44, 4.00]	0.05	.357	− 1.92 (2.45)	[− 6.71, 2.88]	− 0.06	.433
> 65	1.35 (1.49)	[− 1.58, 4.28]	0.05	.367	− 1.39 (2.53)	[− 6.35, 3.57]	− 0.40	.582
Comorbidity Index								
0	Ref				Ref			
1	− 1.36 (0.99)	[− 3.30, 0.58]	− 0.04	.171	− 4.76 (1.28)	[− 7.27, -2.25]	− 0.11	< .001
≥ 2	− 4.43 (1.27)	[− 6.92, -1.94]	− 0.10	< .001	− 9.31 (1.64)	[− 12.52, -6.11]	− 0.17	< .001
Tumor stage								
0	Ref				Ref			
I	− 2.23 (1.12)	[− 4.42, -0.04]	− 0.10	.047	− 2.06 (1.45)	[− 4.91, 0.78]	− 0.07	.155
II	− 2.65 (1.19)	[− 4.97, -0.32]	− 0.11	.026	− 4.77 (1.55)	[− 7.80, -1.74]	− 0.15	.002
III	− 3.22 (1.60)	[− 6.35, -0.08]	− 0.07	.045	− 5.88 (2.15)	[− 10.10, -1.66]	− 0.10	.006

The reference norms for EQ-5D-5L (dimensions, index, and EQ-VAS), EORTC QLQ-C30 and BR23 scores at diagnosis and at the 2-year follow-up are provided in Supplementary data, stratified by age, comorbidity index, and tumor stage (Supplementary materials, Tables 1.1.1 to 2.3.8).

## Discussion

This is the first study to obtain the Spanish reference norms of the EORTC QLQ-C30, the EORTC QLQ-BR23 and the EQ-5D-5L, based on women with non-metastatic breast cancer. The results obtained indicate that the HRQoL of these women differs according to age, comorbidity, and tumor stage at diagnosis and at 2 years of follow-up and, therefore, reference norms based on patient population stratified by these factors are recommended to interpret HRQoL results.

Our results showing HRQoL differences by age and comorbid conditions are consistent with the available evidence from studies on reference norms of the EQ-5D [15–19, 39] and the EORTC QLQ-C30 [21, 22], and confirm the need to stratify them by these variables. According to the magnitude of the HRQoL differences among stages, we decided to provide reference norms separately for stage 0-I and II-III. Previous EORTC QLQ-C30 reference norms also stratified by tumor stage, but with different aggregations (I-II and III-IV in the first one [20], and I-II-III and IV in the 2020 update [21]), both at diagnosis.

There are important differences in content between EQ-5D-5L and EORTC QLQ-C30, as they target different populations. EORTC QLQ-C30 is specifically focused on patients with cancer and, therefore, it includes domains that are especially relevant for this population, such as insomnia or fatigue. The lack of these domains in EQ-5D-5L has been highlighted in patients with multiple myeloma [40] and leukemia [41]. From a clinical perspective, EORTC instruments can be more useful to identify cancer-specific problems, though utilities cannot be directly obtained from EORTC QLQ-C30 and BR23 since they are psychometric instruments. Utilities can be calculated using algorithms developed through mapping models to EQ-5D [42–44]: two developed in patients with metastatic breast cancer (from the EORTC QLQ-C30 [42] or also from the BR-23 [43]) and one specifically developed for HER2-positive patients with recurrent, unresectable, or metastatic breast cancer [44]. These mapping algorithms allow economic evaluations, but administering the utility instrument itself would be the best option: either the EQ–5D–5L or the new breast cancer-specific utility instrument based on the EORTC QLQ-C30 and the BR-45 [45].

The distribution of the EQ–5D–5L index and its five health dimensions shows a marked aggregation of individuals in the best response option (no problems) at diagnosis, which is consistent with the reference norm from Spanish general population [15]. Worse HRQoL in young breast cancer patients than in young general population can be explained by a more drastic HRQoL impact from diagnosis, related to a more advanced stage at diagnosis and worse prognosis. In fact, among the patients aged under 40 years 50% were diagnosed in stage II or III and 6.6% presented metastasis, while among those aged 40–65 years only 33.6% were diagnosed in these stages and 1.7% presented metastasis. This higher HRQoL impact in young breast cancer patients has been previously described in other studies [42, 43].

Women presented worse EORTC QLQ-C30 results at the 2-year follow-up than at diagnosis for both functional and symptom dimensions, except for emotional function, insomnia and appetite loss. Probably these emotional aspects were already impacted just after discovering the cancer diagnosis. These results are consistent with long breast cancer survivors [46], who continue to experience limitations in a variety of HRQoL dimensions, with specific symptoms that persist or even increase over time and HRQoL restrictions not only associated with aging process but also with cancer and/or its treatment.

The women with breast cancer in our study presented similar HRQoL to the non-metastatic patients of the 2020 updated EORTC reference norms [21], which are also based on incident breast cancers cases at diagnosis. However, we obtained better results than the German reference values based on breast cancer patients in routine clinical practice without information about stages [26], and also better than the first EORTC reference norms from 2008 [20], which included patients at diagnosis (17% stage I-II, 14% stage III-IV, 29% not known stage) but also 41% patients recruited with recurrent/metastatic cancer. Our data at 2 years after diagnosis could not be compared with any of the abovementioned reference norms [20, 21, 26] because they did not report any follow-up.

On one hand, we constructed reference norms from a large sample of women with non-metastatic breast cancer consecutively recruited in ten Spanish hospitals, while the 2020 updated EORTC reference norms [21] were based on a polled analysis of randomized clinical trials, usually with restrictive inclusion criteria, which could not be representative of the population of women with breast cancer. On the other hand, our sample was composed mainly by participants from the Basque country, where the project was designed. Due to financial restrictions, the number of hospitals was limited in the other regions in order to recruit around a hundred participants in each one. The results of the comparison among regions followed the well-known geographic pattern of North–South inequalities on the socio-economic indicators, and also on HRQoL with EORTC QLQ-C30 summary means of: 87.0 at diagnosis and 85.5 at 2 years after in the Basque country, 87.3 and 83.6 in Catalonia, and 80.1 and 77.5 in Andalusia (p < 0.001). However, no differences on TNM stage of the breast tumor at diagnosis were found among regions, and the proportion of women diagnosed in the breast screening program in our study is consistent with the 49% reported in another Spanish region [47].

Therefore, as far as we know, this is the first study to provide reference norms based on breast cancer patients at 2 years after diagnosis in the EORTC and EQ–5D–5L, and also the first one specific for Spanish women. Furthermore, it provides values for the EORTC QLQ-BR-23, which was not included in the 2020 updated EORTC reference norms. Although the latter show relevant differences among European regions [21], no values at country level have been provided in these norms. Our study is one of the first, together with the German one [26], providing country-specific breast cancer patients-based reference norms.

As mentioned above, reference norms help to interpret results by comparing them to a control group, in this case Spanish women diagnosed with non-metastatic breast cancer. The difference between the observed score and the reference value provides the individual deviation from the population. For example, the EORCT QLQ-C30 global score of 88 at diagnosis and of 83 2 years later from a 60-year-old woman diagnosed in stage II indicated no deviation from the reference norms for women aged 40–65 years and diagnosed in stage II or III (means of 87 and 83), but these scores indicate a slightly worse HRQoL than expected when compared to the mean of 85, obtained by the whole group aged 40–65 years. This example illustrates why stratification by breast cancer stage could be suitable for the interpretation of HRQoL results.

The main limitation of the study is that CaMISS was not designed specifically to provide HRQoL reference norms, and therefore the recruitment strategy was not addressed to obtaining a sample that were equally representative of all Spanish areas, the North being overrepresented. Second, loss to follow-up is problematic in most cohort studies and often leads to bias. Although it is really small in our sample (13%), it is relevant to consider that women not answering HRQoL questionnaires at 2 years after diagnosis presented a higher rate of recurrences and received more aggressive treatments, which is consistent with results from German and Spanish cohorts of breast cancer patients [48, 49]. Thus, loss to follow-up could have produced and overestimation of HRQoL at the 2-year follow-up. Third, trained interviewers administered the HRQoL questionnaires mainly by telephone interviews before treatment, while almost all patients self-completed them at home, as recommended, 2 years after diagnosis. However, little difference between both administration methods has been reported [50]. Finally, no reference norms had been provided for the new EORTC BR-45 [51], an update of the BR-23 originally developed in 1996.

Having reference norms based on Spanish patients with non-metastatic breast cancer is fundamental, as they will facilitate the assessment of the impact of this disease, monitoring its evolution, comparing the impact of treatments, identifying populations that need special attention, and carrying out comparisons among different countries. These reference norms can be useful to interpret the scores obtained in women with non-metastatic breast cancer, who are the majority in Spain, by comparing them with country-specific reference values for this population.

### Supplementary Information

Below is the link to the electronic supplementary material.

Supplementary file1 (DOCX 656 kb)
