# Correction: Reference values of EORTC QLQ-C30, EORTC QLQ-BR23, and EQ-5D-5L for women with non-metastatic breast cancer at diagnosis and 2 years after

**DOI:** 10.1007/s11136-024-03628-w

**Published:** 2024-03-08

**Authors:** Carme Miret, Miren Orive, Maria Sala, Susana García-Gutiérrez, Cristina Sarasqueta, Maria Jose Legarreta, Maximino Redondo, Amado Rivero, Xavier Castells, José M. Quintana, Olatz Garin, Montse Ferrer, the REDISSEC-CaMISS Group, Mercè Comas, Laia Domingo, Francesc Macià, Marta Roman, Anabel Romero, Teresa Barata, Isabel Diez de la Lastra, Mariola de la Vega, Marisa Bare, Núria Torà, Joana Ferrer, Francesc Castanyer, Carmen Carmona, Susana García, Maximina Martín, Nerea Gonzalez, Maria Amparo Valverde, Alberto Saez, Inma Barredo, Manuel de Toro, Josefa Ferreiro, Jeanette Pérez, Cristina Valcárcel, María del Carmen Padilla, Teresa Téllez, Irene Zarcos, Cristina Churruca, Amaia Perales, Javier Recio, Irune Ruiz, Jose María Urraca, MªJesús Michelena, Julio Moreno, Gaizka Mallabiabarrena, Patricia Cobos, Borja Otero, Javier Gorostiaga, Itsaso Troya

**Affiliations:** 1 Department of Pediatrics, Obstetrics and Gynecology, Preventive Medicine and Public Health, Universitat Autònoma de Barcelona (UAB), 08193 Bellaterra, Barcelona, Spain; 2 Preventive Medicine and Public Health Training Unit PSMar-UPF-ASPB, Parc de Salut Mar, Agència de Salut Pública de Barcelona, i Universitat Pompeu Fabra, Barcelona, Spain; 3 IMIM (Hospital del Mar Medical Research Institute), Barcelona, Spain; 4 Departamento Psicología Social, Facultad Farmacia, UPV/EHU, Vitoria-Gasteiz, Araba Spain; 5 Osakidetza Basque Health Service, Research Unit, Galdakao-Usansolo University Hospital, Galdakao, Bizkai Spain; 6 KRONIKGUNE-Institute for Health Service Research, Barakaldo, Bizkaia Spain; 7 Health Services Research on Chronic Patients Network (REDISSEC), Galdakao, Bizkaia Spain; 8 Red de Investigación en Cronicidad, Atención Primaria y Promoción de la Salud (RICAPPS), Madrid, Spain; 9 Biodonostia Health Research Institute, Donostia University Hospital, Donostia, Gipuzkoa Spain; 10 Research and Innovation Unit, Hospital Costa del Sol, Marbella, Spain; 11 Servicio de Evaluación y Planificación del Servicio Canario de la Salud (SESCS), Tenerife, Spain; 12 Health Services Research Group, IMIM (Hospital del Mar Medical Research Institute), Doctor Aiguader 88, 08003 Barcelona, Spain; 13 CIBER en Epidemiología y Salud Pública (CIBERESP), Madrid, Spain; 14 Departament de Medicina i Ciències de la Vida, Universitat Pompeu Fabra (UPF), Barcelona, Spain

**Correction to: Quality of Life Research (2023) 32:989–1003** 10.1007/s11136-022-03327-4

In the original publication of the article, the authors realized that the scores of two dimensions of the EORTC QLQ BR23 (Sexual Function and Sexual Enjoyment) were wrongly reversed while preparing the tables. This mistake affects two lines (Sexual Function and Sexual Enjoyment) of Table 4 and Supplementary Table (1.3.2, 1.3.3, 2.3.2 and 2.3.3). The correct version of Table 4 and the supplementary information are provided in this correction.

**Table 4:** Mean scores and standard deviations of EORTC QLQ-BR-23 according to age, Charlson Comorbidity Index, and stage

	All	Age				Comorbidity index			Tumor stage				
		< 40	40–65	> 65	*p* value*	0	≥ 1	*p* value*	0	I	II	III	*p* value*
At diagnosis													
Body Image	92.3 (16.7)	87.5 (20.8)	92.0 (17.0)	94.5 (14.4)	0.003	92.4 (16.3)	92.1 (18.4)	0.779	94.2 (12.6)	92.6 (16.7)	91.9 (17.2)	89.8 (19.8)	0.258
Sexual Function	23.5 (26.1)	31.3 (27.1)	27.4 (26.6)	10.6 (19.2)	< 0.001	24.9 (25.8)	18.2 (26.5)	< 0.001	24.7 (26.5)	24.9 (26.1)	21.1 (25.4)	21.8 (28.3)	0.126
Sexual enjoy	54.6 (29.1)	59.4 (32.1)	56.2 (28.8)	42.5 (26.1)	< 0.001	55.3 (28.9)	50.5 (29.9)	0.140	54.5 (26.6)	54.8 (28.3)	53.6 (29.7)	57 (36.0)	0.913
Future perspective	45.9 (31.9)	36.4 (32.0)	45.7 (31.7)	48.8 (32.2)	0.009	45.8 (31.3)	46.3 (34.2)	0.822	50.3 (29.9)	45.8 (32.1)	46.4 (32.4)	39.2 (31.3)	0.090
Systemic therapy effects	12.5 (13.9)	11.5 (13.9)	12.7 (14.4)	12.4 (12.7)	0.764	12.2 (13.8)	13.6 (14.3)	0.169	12.6 (13.5)	11.8 (13.2)	13.2 (14.8)	14.4 (16.2)	0.235
Breast symptoms	13.2 (16.9)	19.2 (20.4)	13.4 (16.9)	11.1 (15.8)	0.001	13.7 (17.2)	11.0 (15.6)	0.021	13.4 (17.2)	12.5 (17.2)	14.4 (16.7)	12.9 (15.8)	0.342
Arm symptoms	8.7 (15.6)	6.4 (12.5)	8.4 (14.7)	10.0 (18.3)	0.127	8.2 (14.7)	11.0 (18.5)	0.011	7.8 (14.0)	8.4 (15.5)	9.6 (15.8)	8.6 (17.2)	0.592
Upset hair loss	24.0 (31.6)	12.3 (27.7)	25.6 (33.2)	23.6 (27.7)	0.223	23.5 (31.4)	26.7 (33.1)	0.564	11.1 (18.5)	22.9 (32.8)	28.6 (32.5)	33.3 (31.2)	0.053
At 2-year follow-up													
Body Image	84.6 (24.6)	79.7 (28.6)	82.9 (25.8)	88.8 (21.0)	0.001	84.6 (24.7)	84.7 (24.4)	0.956	90.1 (18.3)	86.7 (22.4)	81.4 (27.0)	73.5 (33.7)	< 0.001
Sexual Function	20.9 (23.5)	29.5 (21.0)	25 (24.1)	9.9 (17.9)	< 0.001	22.4 (24.0)	15.4 (21.0)	< 0.001	23.8 (24.1)	21.7 (24.3)	18.9 (22.5)	19 (19.8)	0.163
Sexual enjoy	50.3 (27.8)	62.8 (23.7)	53.2 (27.0)	34.3 (27.1)	< 0.001	51.3 (28.1)	45.6 (26.2)	0.070	52.8 (26.3)	51.3 (29.2)	48.2 (27.1)	47 (22.6)	0.518
Future perspective	59.5 (32.1)	55.9 (37.4)	57.1 (31.9)	65.0 (31.4)	0.001	59.2 (31.9)	60.6 (33.0)	0.557	66.4 (29.3)	59.6 (31.6)	59.1 (32.4)	48.4 (36.9)	0.003
Systemic therapy effects	18.3 (16.5)	12.2 (11.1)	18.9 (16.8)	17.9 (16.1)	0.056	17.7 (16.3)	20.7 (17.3)	0.014	16.6 (15.4)	17.8 (15.8)	19.3 (17.8)	21.1 (17.8)	0.171
Breast symptoms	16.0 (17.8)	17.4 (19.5)	17.4 (18.0)	13.0 (16.9)	0.001	15.8 (16.8)	16.6 (21.0)	0.572	15.0 (18.5)	16.0 (17.0)	16.1 (18.7)	17.2 (18.5)	0.859
Arm symptoms	15.7 (20.3)	9.5 (15.0)	16.2 (20.3)	15.5 (20.8)	0.157	15.0 (19.5)	18.2 (23.0)	0.035	12.0 (18.0)	14.2 (19.4)	18.2 (21.0)	22.8 (24.8)	< 0.001
Upset hair loss	31.1 (33.7)	13.9 (30.0)	32.0 (33.4)	31.2 (34.3)	0.192	29.4 (32.6)	36.9 (36.7)	0.058	22.0 (26.8)	29.8 (33.4)	34.4 (34.5)	42.0 (40.5)	0.066

*EORTC QLQ-BR-23 differences according to age, comorbidity and stage were tested with ANOVA

The original article has been corrected.

### Supplementary Information

Below is the link to the electronic supplementary material.

Supplementary file1 (DOCX 655 kb)

