# Supplementary material for: Reference values of EORTC QLQ-C30, EORTC QLQ-BR23, and EQ-5D-5L for women with non-metastatic breast cancer at diagnosis and 2 years after
Source: Qual Life Res. 2023 Jan 11;32(4):989–1003. doi: 10.1007/s11136-022-03327-4 (PMC10063520; doi:10.1007/s11136-022-03327-4)
Supplement: Supplementary file 1 — Supplementary file1 (DOCX 656 kb) [file 11136_2022_3327_MOESM1_ESM.docx]

Supplementary tables

[**1. REFERENCE NORMS AT DIAGNOSIS** 2](#_Toc104375886)

[Supplementary Table 1.1.1. Reference norms at diagnosis for the EQ-5D-5L Index. 2](#_Toc104375887)

[Supplementary Table 1.1.2. Reference norms at diagnosis for the EQ-VAS. 3](#_Toc104375888)

[Supplementary table 1.1.3. Reference norms at diagnosis for the dimensions of the EQ-5D-5L. 3](#_Toc104375889)

[Supplementary table 1.2.1. Reference norms at diagnosis for the Summary score of EORTC QLQ-C30. 6](#_Toc104375890)

[Supplementary table 1.2.2. Reference norms at diagnosis for the Physical function of EORTC QLQ-C30. 7](#_Toc104375891)

[Supplementary table 1.2.3. Reference norms at diagnosis for the Role function of EORTC QLQ-C30. 8](#_Toc104375892)

[Supplementary table 1.2.4. Reference norms at diagnosis for the Emotional function of EORTC QLQ-C30. 9](#_Toc104375893)

[Supplementary table 1.2.5. Reference norms at diagnosis for the Cognitive function of EORTC QLQ-C30. 10](#_Toc104375894)

[Supplementary table 1.2.6. Reference norms at diagnosis for the Social function of EORTC QLQ-C30. 11](#_Toc104375895)

[Supplementary table 1.2.7. Reference norms at diagnosis for the Global health status of EORTC QLQ-C30. 12](#_Toc104375896)

[Supplementary table 1.2.8. Reference norms at diagnosis for the Fatigue of EORTC QLQ-C30. 13](#_Toc104375897)

[Supplementary table 1.2.9. Reference norms at diagnosis for the Nausea of EORTC QLQ-C30. 14](#_Toc104375898)

[Supplementary table 1.2.10. Reference norms at diagnosis for the Pain of EORTC QLQ-C30. 15](#_Toc104375899)

[Supplementary table 1.2.11. Reference norms at diagnosis for the Dyspnea of EORTC QLQ-C30. 16](#_Toc104375900)

[Supplementary table 1.2.12. Reference norms at diagnosis for the Insomnia of EORTC QLQ-C30. 17](#_Toc104375901)

[Supplementary table 1.2.13. Reference norms at diagnosis for the Appetite loss of EORTC QLQ-C30. 18](#_Toc104375902)

[Supplementary table 1.2.14. Reference norms at diagnosis for the Constipation of EORTC QLQ-C30. 19](#_Toc104375903)

[Supplementary table 1.2.15. Reference norms at diagnosis for the Diarrhea of EORTC QLQ-C30. 20](#_Toc104375904)

[Supplementary table 1.2.16. Reference norms at diagnosis for the Financial difficulties of EORTC QLQ-C30. 21](#_Toc104375905)

[Supplementary table 1.3.1. Reference norms at diagnosis for the Body Image Scale of EORTC QLQ-BR23. 22](#_Toc104375906)

[Supplementary table 1.3.2. Reference norms at diagnosis for the Sexual Function Scale of EORTC QLQ-BR23. 23](#_Toc104375907)

[Supplementary table 1.3.3. Reference norms at diagnosis for the Sexual Enjoyment Scale of EORTC QLQ-BR23. 24](#_Toc104375908)

[Supplementary table 1.3.4. Reference norms at diagnosis for the Future perspective Scale of EORTC QLQ-BR23. 25](#_Toc104375909)

[Supplementary table 1.3.5. Reference norms at diagnosis for the Systemic Therapy Side Effects Scale of EORTC QLQ-BR23. 26](#_Toc104375910)

[Supplementary table 1.3.6. Reference norms at diagnosis for the Breast Symptoms Scale of EORTC QLQ-BR23. 27](#_Toc104375911)

[Supplementary table 1.3.7. Reference norms at diagnosis for the Arm Symptoms Scale of EORTC QLQ-BR23. 28](#_Toc104375912)

[Supplementary table 1.3.8. Reference norms at diagnosis for the Upset by Hair Loss Scale of EORTC QLQ-BR23. 29](#_Toc104375913)

[**2. REFERENCE NORMS AT 2 YEARS** 30](#_Toc104375914)

[Supplementary table 2.1.1. Reference norms at 2-year follow-up for the EQ-5D-5L Index. 30](#_Toc104375915)

[Supplementary table 2.1.2. Reference norms at 2-year follow-up for the EQ-VAS. 31](#_Toc104375916)

[Supplementary table 2.1.3. Reference norms at 2-year follow-up for the dimensions of the EQ-5D-5L. 32](#_Toc104375917)

[Supplementary table 2.2.1. Reference norms at 2-year follow-up for the Summary score of EORTC QLQ-C30. 34](#_Toc104375918)

[Supplementary table 2.2.2. Reference norms at 2-year follow-up for the Physical function of EORTC QLQ-C30. 35](#_Toc104375919)

[Supplementary table 2.2.3. Reference norms at 2-year follow-up for the Role function of EORTC QLQ-C30. 36](#_Toc104375920)

[Supplementary table 2.2.4. Reference norms at 2-year follow-up for the Emotional function of EORTC QLQ-C30. 37](#_Toc104375921)

[Supplementary table 2.2.5. Reference norms at 2-year follow-up for the Cognitive function of EORTC QLQ-C30. 38](#_Toc104375922)

[Supplementary table 2.2.6. Reference norms at 2-year follow-up for the Social function of EORTC QLQ-C30. 39](#_Toc104375923)

[Supplementary table 2.2.7. Reference norms at 2-year follow-up for the Global health status of EORTC QLQ-C30. 40](#_Toc104375924)

[Supplementary table 2.2.8. Reference norms at 2-year follow-up for the Fatigue of EORTC QLQ-C30. 41](#_Toc104375925)

[Supplementary table 2.2.9. Reference norms at 2-year follow-up for the Nausea of EORTC QLQ-C30. 42](#_Toc104375926)

[Supplementary table 2.2.10. Reference norms at 2-year follow-up for the Pain of EORTC QLQ-C30. 43](#_Toc104375927)

[Supplementary table 2.2.11. Reference norms at 2-year follow-up for the Dyspnea of EORTC QLQ-C30. 44](#_Toc104375928)

[Supplementary table 2.2.12. Reference norms at 2-year follow-up for the Insomnia of EORTC QLQ-C30. 45](#_Toc104375929)

[Supplementary table 2.2.13. Reference norms at 2-year follow-up for the Appetite loss of EORTC QLQ-C30. 46](#_Toc104375930)

[Supplementary table 2.2.14. Reference norms at 2-year follow-up for the Constipation of EORTC QLQ-C30. 47](#_Toc104375931)

[Supplementary table 2.2.15. Reference norms at 2-year follow-up for the Diarrhea of EORTC QLQ-C30. 48](#_Toc104375932)

[Supplementary table 2.2.16. Reference norms at 2-year follow-up for the Financial difficulties of EORTC QLQ-C30. 49](#_Toc104375933)

[Supplementary table 2.3.1. Reference norms at 2-year follow-up for the Body Image Scale of EORTC QLQ-BR23. 50](#_Toc104375934)

[Supplementary table 2.3.2. Reference norms at 2-year follow-up for the Sexual Function Scale of EORTC QLQ-BR23. 51](#_Toc104375935)

[Supplementary table 2.3.3. Reference norms for the Sexual Enjoyment Scale of EORTC QLQ-BR23. 52](#_Toc104375936)

[Supplementary table 2.3.4. Reference norms at 2-year follow-up for the Future perspective Scale of EORTC QLQ-BR23. 53](#_Toc104375937)

[Supplementary table 2.3.5. Reference norms at 2-year follow-up for the Systemic Therapy Side Effects Scale of EORTC QLQ-BR23. 54](#_Toc104375938)

[Supplementary table 2.3.6. Reference norms at 2-year follow-up for the Breast Symptoms Scale of EORTC QLQ-BR23. 55](#_Toc104375939)

[Supplementary table 2.3.7. Reference norms at 2-year follow-up for the Arm Symptoms Scale of EORTC QLQ-BR23. 56](#_Toc104375940)

[Supplementary table 2.3.8. Reference norms at 2-year follow-up for the Upset by Hair Loss Scale of EORTC QLQ-BR23. 57](#_Toc104375941)

# REFERENCE NORMS AT DIAGNOSIS

# Supplementary Table 1.1.1. Reference norms at diagnosis for the EQ-5D-5L Index.

|  |  | **EQ-5D-5L Index** | | | | |
| --- | --- | --- | --- | --- | --- | --- |
|  |  |  | **Charlson** | | **TNM stage** | |
|  |  | **All** | **0** | **≥ 1** | **0 – I** | **II - III** |
| **Age (years)**  **<40** | **n** | 75 | 71 | 4 | 38 | 37 |
|  | **Mean (SD)** | 0.880 (0.115) | 0.882 (0.114) | 0.838 (0.147) | 0.868 (0.125) | 0.892 (0.105) |
|  | **95%CI** | [ 0.854 - 0.906 ] | [ 0.855 - 0.909 ] | [ 0.693 - 0.982 ] | [ 0.828 - 0.908 ] | [ 0.858 - 0.926 ] |
|  | **P 5** | 0.621 | 0.636 |  | 0.605 | 0.619 |
|  | **P 10** | 0.725 | 0.728 |  | 0.667 | 0.726 |
|  | **P 20** | 0.794 | 0.794 |  | 0.767 | 0.841 |
|  | **P 25** | 0.841 | 0.841 |  | 0.794 | 0.841 |
|  | **P 30** | 0.841 | 0.841 |  | 0.811 | 0.854 |
|  | **P 40** | 0.872 | 0.872 |  | 0.872 | 0.872 |
|  | **Median** | 0.899 | 0.899 | 0.886 | 0.896 | 0.899 |
|  | **P 60** | 0.919 | 0.919 |  | 0.919 | 0.919 |
|  | **P 70** | 0.965 | 1.000 |  | 0.932 | 1.000 |
|  | **P 75** | 1.000 | 1.000 |  | 1.000 | 1.000 |
|  | **P 80** | 1.000 | 1.000 |  | 1.000 | 1.000 |
|  | **P 90** | 1.000 | 1.000 |  | 1.000 | 1.000 |
|  | **P 95** | 1.000 | 1.000 |  | 1.000 | 1.000 |
| **40-65** | **n** | 875 | 738 | 137 | 580 | 295 |
|  | **Mean (SD)** | 0.871 (0.132) | 0.878 (0.122) | 0.834 (0.172) | 0.871 (0.135) | 0.871 (0.128) |
|  | **95%CI** | [ 0.862 - 0.880 ] | [ 0.869 - 0.887 ] | [ 0.805 - 0.863 ] | [ 0.860 - 0.882 ] | [ 0.857 - 0.886 ] |
|  | **P 5** | 0.628 | 0.652 | 0.392 | 0.620 | 0.649 |
|  | **P 10** | 0.727 | 0.730 | 0.619 | 0.730 | 0.711 |
|  | **P 20** | 0.794 | 0.797 | 0.732 | 0.794 | 0.794 |
|  | **P 25** | 0.828 | 0.841 | 0.773 | 0.841 | 0.818 |
|  | **P 30** | 0.841 | 0.841 | 0.794 | 0.841 | 0.841 |
|  | **P 40** | 0.872 | 0.872 | 0.872 | 0.872 | 0.872 |
|  | **Median** | 0.919 | 0.919 | 0.872 | 0.919 | 0.899 |
|  | **P 60** | 0.919 | 0.919 | 0.919 | 0.919 | 0.919 |
|  | **P 70** | 0.922 | 0.922 | 0.919 | 0.922 | 0.922 |
|  | **P 75** | 1.000 | 1.000 | 0.922 | 1.000 | 1.000 |
|  | **P 80** | 1.000 | 1.000 | 1.000 | 1.000 | 1.000 |
|  | **P 90** | 1.000 | 1.000 | 1.000 | 1.000 | 1.000 |
|  | **P 95** | 1.000 | 1.000 | 1.000 | 1.000 | 1.000 |
| **>65** | **n** | 317 | 203 | 114 | 177 | 140 |
|  | **Mean (SD)** | 0.829 (0.167) | 0.851 (0.150) | 0.791 (0.190) | 0.845 (0.161) | 0.810 (0.173) |
|  | **95%CI** | [ 0.811 - 0.848 ] | [ 0.830 - 0.871 ] | [ 0.756 - 0.826 ] | [ 0.821 - 0.868 ] | [ 0.781 - 0.839 ] |
|  | **P 5** | 0.465 | 0.546 | 0.375 | 0.477 | 0.390 |
|  | **P 10** | 0.624 | 0.655 | 0.507 | 0.623 | 0.624 |
|  | **P 20** | 0.729 | 0.754 | 0.643 | 0.742 | 0.713 |
|  | **P 25** | 0.756 | 0.794 | 0.689 | 0.790 | 0.730 |
|  | **P 30** | 0.794 | 0.795 | 0.729 | 0.800 | 0.757 |
|  | **P 40** | 0.841 | 0.841 | 0.800 | 0.841 | 0.798 |
|  | **Median** | 0.872 | 0.878 | 0.841 | 0.899 | 0.856 |
|  | **P 60** | 0.919 | 0.919 | 0.872 | 0.919 | 0.878 |
|  | **P 70** | 0.919 | 0.919 | 0.919 | 0.919 | 0.919 |
|  | **P 75** | 0.919 | 0.956 | 0.919 | 1.000 | 0.919 |
|  | **P 80** | 1.000 | 1.000 | 0.919 | 1.000 | 0.919 |
|  | **P 90** | 1.000 | 1.000 | 1.000 | 1.000 | 1.000 |
|  | **P 95** | 1.000 | 1.000 | 1.000 | 1.000 | 1.000 |

**Abbreviations**. **SD**: Standard Deviation; **95% CI**: 95% Confidence Interval; **P**: Percentile.

# Supplementary Table 1.1.2. Reference norms at diagnosis for the EQ-VAS.

|  |  | **EQ-VAS** | | | | |
| --- | --- | --- | --- | --- | --- | --- |
|  |  |  | **Charlson** | | **TNM stage** | |
|  |  | **All** | **Charlson = 0** | **Charlson ≥ 1** | **0 – I** | **II - III** |
| **Age (years)**  **<40** | **n** | 74 | 70 | 4* | 38 | 36 |
|  | **Mean (SD)** | 74.2 (21.5) | 74.7 (21.0) | 65.0 (31.1) | 78.2 (17.9) | 70.0 (24.2) |
|  | **95%CI** | [ 69.3 - 79.1 ] | [ 69.8 - 79.6 ] | [ 34.5 - 95.5 ] | [ 72.5 - 83.9 ] | [ 62.1 - 77.9 ] |
|  | **P 5** | 33.8 | 37.8 |  | 40.0 | 27.0 |
|  | **P 10** | 40.0 | 40.5 |  | 49.5 | 33.5 |
|  | **P 20** | 50.0 | 50.0 |  | 62.0 | 47.0 |
|  | **P 25** | 57.5 | 60.0 |  | 70.0 | 50.0 |
|  | **P 30** | 70.0 | 70.0 |  | 70.0 | 51.0 |
|  | **P 40** | 70.0 | 72.0 |  | 80.0 | 70.0 |
|  | **Median** | 80.0 | 80.0 | 65.0 | 80.0 | 75.0 |
|  | **P 60** | 85.0 | 85.0 |  | 90.0 | 80.0 |
|  | **P 70** | 90.0 | 90.0 |  | 90.0 | 90.0 |
|  | **P 75** | 90.0 | 90.0 |  | 90.0 | 90.0 |
|  | **P 80** | 90.0 | 90.0 |  | 91.0 | 90.0 |
|  | **P 90** | 100.0 | 100.0 |  | 100.0 | 100.0 |
|  | **P 95** | 100.0 | 100.0 |  | 100.0 | 100.0 |
| **40-65** | **n** | 871 | 734 | 137 | 579 | 292 |
|  | **Mean (SD)** | 73.5 (19.9) | 74.5 (19.0) | 68.2 (23.3) | 74.2 (19.1) | 72.2 (21.3) |
|  | **95%CI** | [ 72.2 - 74.8 ] | [ 73.1 - 75.9 ] | [ 64.3 - 72.1 ] | [ 72.6 - 75.7 ] | [ 69.7 - 74.6 ] |
|  | **P 5** | 40.0 | 40.0 | 20.0 | 40.0 | 30.0 |
|  | **P 10** | 50.0 | 50.0 | 40.0 | 50.0 | 50.0 |
|  | **P 20** | 60.0 | 60.0 | 50.0 | 60.0 | 50.0 |
|  | **P 25** | 60.0 | 65.0 | 50.0 | 60.0 | 60.0 |
|  | **P 30** | 70.0 | 70.0 | 60.0 | 70.0 | 70.0 |
|  | **P 40** | 70.0 | 70.0 | 70.0 | 70.0 | 70.0 |
|  | **Median** | 80.0 | 80.0 | 70.0 | 80.0 | 80.0 |
|  | **P 60** | 80.0 | 80.0 | 80.0 | 80.0 | 80.0 |
|  | **P 70** | 85.0 | 87.5 | 80.0 | 85.0 | 85.0 |
|  | **P 75** | 90.0 | 90.0 | 85.0 | 90.0 | 90.0 |
|  | **P 80** | 90.0 | 90.0 | 90.0 | 90.0 | 90.0 |
|  | **P 90** | 95.0 | 95.0 | 95.0 | 98.0 | 95.0 |
|  | **P 95** | 100.0 | 100.0 | 100.0 | 100.0 | 100.0 |
| **>65** | **n** | 315 | 201 | 114 | 176 | 139 |
|  | **Mean (SD)** | 71.9 (19.5) | 74.9 (17.9) | 66.6 (21.3) | 72.9 (18.2) | 70.7 (21.2) |
|  | **95%CI** | [ 69.7 - 74.1 ] | [ 72.5 - 77.4 ] | [ 62.7 - 70.5 ] | [ 70.2 - 75.5 ] | [ 67.2 - 74.2 ] |
|  | **P 5** | 40.0 | 40.0 | 30.0 | 40.0 | 30.0 |
|  | **P 10** | 45.0 | 50.0 | 40.0 | 50.0 | 40.0 |
|  | **P 20** | 51.0 | 60.0 | 50.0 | 55.0 | 50.0 |
|  | **P 25** | 60.0 | 61.0 | 50.0 | 60.0 | 60.0 |
|  | **P 30** | 60.0 | 70.0 | 50.0 | 60.2 | 60.0 |
|  | **P 40** | 70.0 | 75.0 | 60.0 | 70.0 | 70.0 |
|  | **Median** | 80.0 | 80.0 | 70.0 | 80.0 | 75.0 |
|  | **P 60** | 80.0 | 80.0 | 80.0 | 80.0 | 80.0 |
|  | **P 70** | 81.0 | 85.0 | 80.0 | 80.0 | 85.0 |
|  | **P 75** | 85.0 | 90.0 | 80.0 | 85.0 | 85.0 |
|  | **P 80** | 90.0 | 90.0 | 85.0 | 90.0 | 90.0 |
|  | **P 90** | 95.0 | 95.0 | 90.0 | 95.0 | 95.0 |
|  | **P 95** | 100.0 | 100.0 | 100.0 | 100.0 | 100.0 |

* Deciles were not calculated for subgroups lower than 20 participants.

**Abbreviations. SD:** Standard Deviation; **95% CI:** 95% Confidence Interval; **P:** Percentile.

# Supplementary table 1.1.3. Reference norms at diagnosis for the dimensions of the EQ-5D-5L.

|  |  | **All** | | **Charlson** | | | | **TNM** | | | |
| --- | --- | --- | --- | --- | --- | --- | --- | --- | --- | --- | --- |
|  |  | **All** | | **0** | | **≥ 1** | | **0 – I** | | **II - III** | |
| **Age (years)** |  | **%** | **SE** | **%** | **SE** | **%** | **SE** | **%** | **SE** | **%** | **SE** |
| **Mobility** |  |  |  |  |  |  |  |  |  |  |  |
| **<40**  **(n = 76)** | **No problems** | 96.1% | 0.02 | 97.2% | 0.02 | * | | 97.4% | 0.02 | 94.7% | 0.03 |
|  | **Slight problems** | 2.6% | 0.02 | 2.8% | 0.02 |  |  | 2.6% | 0.02 | 2.6% | 0.02 |
|  | **Moderate problems** | 1.3% | 0.01 | 0.0% | 0.00 |  |  | 0.0% | 0.00 | 2.6% | 0.02 |
|  | **Severe problems** | 0.0% | 0.00 | 0.0% | 0.00 |  |  | 0.0% | 0.00 | 0.0% | 0.00 |
|  | **Unable** | 0.0% | 0.00 | 0.0% | 0.00 |  |  | 0.0% | 0.00 | 0.0% | 0.00 |
| **40-65**  **(n = 878)** | **No problems** | 93.4% | 0.01 | 95.7% | 0.01 | 81.3% | 0.01 | 94.3% | 0.01 | 91.5% | 0.01 |
|  | **Slight problems** | 3.9% | 0.01 | 2.6% | 0.01 | 10.8% | 0.01 | 2.9% | 0.01 | 5.8% | 0.01 |
|  | **Moderate problems** | 1.9% | 0.00 | 1.4% | 0.00 | 5.0% | 0.01 | 1.9% | 0.00 | 2.0% | 0.00 |
|  | **Severe problems** | 0.6% | 0.00 | 0.3% | 0.00 | 2.2% | 0.00 | 0.7% | 0.00 | 0.3% | 0.00 |
|  | **Unable** | 0.2% | 0.00 | 0.1% | 0.00 | 0.7% | 0.00 | 0.2% | 0.00 | 0.3% | 0.00 |
| **>65**  **(n = 320)** | **No problems** | 73.4% | 0.02 | 81.0% | 0.02 | 60.0% | 0.03 | 75.1% | 0.02 | 71.3% | 0.03 |
|  | **Slight problems** | 11.9% | 0.02 | 9.3% | 0.02 | 16.5% | 0.02 | 10.7% | 0.02 | 13.3% | 0.02 |
|  | **Moderate problems** | 12.2% | 0.02 | 7.8% | 0.01 | 20.0% | 0.02 | 13.0% | 0.02 | 11.2% | 0.02 |
|  | **Severe problems** | 2.5% | 0.01 | 2.0% | 0.01 | 3.5% | 0.01 | 1.1% | 0.01 | 4.2% | 0.01 |
|  | **Unable** | 0.0% | 0.00 | 0.0% | 0.00 | 0.0% | 0.00 | 0.0% | 0.00 | 0.0% | 0.00 |
| **Self-Care** |  |  |  |  |  |  |  |  |  |  |  |
| **<40**  **(n = 76)** | **No problems** | 96.1% | 0.02 | 95.8% | 0.02 |  |  | 92.1% | 0.03 | 100.0% | 0.00 |
|  | **Slight problems** | 2.6% | 0.02 | 2.8% | 0.02 |  |  | 5.3% | 0.03 | 0.0% | 0.00 |
|  | **Moderate problems** | 1.3% | 0.01 | 1.4% | 0.01 |  |  | 2.6% | 0.02 | 0.0% | 0.00 |
|  | **Severe problems** | 0.0% | 0.00 | 0.0% | 0.00 |  |  | 0.0% | 0.00 | 0.0% | 0.00 |
|  | **Unable** | 0.0% | 0.00 | 0.0% | 0.00 |  |  | 0.0% | 0.00 | 0.0% | 0.00 |
| **40-65**  **(n = 876)** | **No problems** | 96.7% | 0.01 | 97.0% | 0.01 | 94.9% | 0.01 | 97.2% | 0.01 | 95.6% | 0.01 |
|  | **Slight problems** | 2.2% | 0.00 | 2.3% | 0.01 | 1.5% | 0.00 | 1.9% | 0.00 | 2.7% | 0.01 |
|  | **Moderate problems** | 0.8% | 0.00 | 0.5% | 0.00 | 2.2% | 0.00 | 0.3% | 0.00 | 1.7% | 0.00 |
|  | **Severe problems** | 0.3% | 0.00 | 0.1% | 0.00 | 1.5% | 0.00 | 0.5% | 0.00 | 0.0% | 0.00 |
|  | **Unable** | 0.0% | 0.00 | 0.0% | 0.00 | 0.0% | 0.00 | 0.0% | 0.00 | 0.0% | 0.00 |
| **>65**  **(n = 320)** | **No problems** | 91.6% | 0.02 | 95.1% | 0.01 | 85.2% | 0.02 | 91.0% | 0.02 | 92.3% | 0.01 |
|  | **Slight problems** | 3.8% | 0.01 | 2.9% | 0.01 | 5.2% | 0.01 | 4.5% | 0.01 | 2.8% | 0.01 |
|  | **Moderate problems** | 4.1% | 0.01 | 2.0% | 0.01 | 7.8% | 0.02 | 4.0% | 0.01 | 4.2% | 0.01 |
|  | **Severe problems** | 0.6% | 0.00 | 0.0% | 0.00 | 1.7% | 0.01 | 0.6% | 0.00 | 0.7% | 0.00 |
|  | **Unable** | 0.0% | 0.00 | 0.0% | 0.00 | 0.0% | 0.00 | 0.0% | 0.00 | 0.0% | 0.00 |
| **Usual Activities** | |  |  |  |  |  |  |  |  |  |  |
| **<40**  **(n = 76)** | **No problems** | 82.9% | 0.04 | 84.7% | 0.04 |  |  | 86.8% | 0.04 | 78.9% | 0.05 |
|  | **Slight problems** | 10.5% | 0.04 | 9.7% | 0.03 |  |  | 7.9% | 0.03 | 13.2% | 0.04 |
|  | **Moderate problems** | 5.3% | 0.03 | 4.2% | 0.02 |  |  | 2.6% | 0.02 | 7.9% | 0.03 |
|  | **Severe problems** | 0.0% | 0.00 | 0.0% | 0.00 |  |  | 0.0% | 0.00 | 0.0% | 0.00 |
|  | **Unable** | 1.3% | 0.01 | 1.4% | 0.01 |  |  | 2.6% | 0.02 | 0.0% | 0.00 |
| **40-65**  **(n = 877)** | **No problems** | 90.3% | 0.01 | 91.1% | 0.01 | 86.3% | 0.01 | 91.6% | 0.01 | 87.8% | 0.01 |
|  | **Slight problems** | 5.7% | 0.01 | 5.4% | 0.01 | 7.2% | 0.01 | 5.2% | 0.01 | 6.8% | 0.01 |
|  | **Moderate problems** | 3.0% | 0.01 | 2.6% | 0.01 | 5.0% | 0.01 | 2.7% | 0.01 | 3.4% | 0.01 |
|  | **Severe problems** | 0.8% | 0.00 | 0.8% | 0.00 | 0.7% | 0.00 | 0.3% | 0.00 | 1.7% | 0.00 |
|  | **Unable** | 0.2% | 0.00 | 0.1% | 0.00 | 0.7% | 0.00 | 0.2% | 0.00 | 0.3% | 0.00 |
| **>65**  **(n = 320)** | **No problems** | 80.0% | 0.02 | 82.9% | 0.02 | 74.8% | 0.02 | 82.5% | 0.02 | 76.9% | 0.02 |
|  | **Slight problems** | 10.6% | 0.02 | 10.7% | 0.02 | 10.4% | 0.02 | 9.0% | 0.02 | 12.6% | 0.02 |
|  | **Moderate problems** | 6.3% | 0.01 | 5.4% | 0.01 | 7.8% | 0.02 | 5.1% | 0.01 | 7.7% | 0.01 |
|  | **Severe problems** | 2.5% | 0.01 | 1.0% | 0.01 | 5.2% | 0.01 | 2.3% | 0.01 | 2.8% | 0.01 |
|  | **Unable** | 0.6% | 0.00 | 0.0% | 0.00 | 1.7% | 0.01 | 1.1% | 0.01 | 0.0% | 0.00 |
| **Pain** |  |  |  |  |  |  |  |  |  |  |  |
| **<40**  **(n = 75)** | **No pain** | 60.0% | 0.06 | 60.6% | 0.06 |  |  | 63.2% | 0.06 | 56.8% | 0.06 |
|  | **Slight pain** | 25.3% | 0.05 | 26.8% | 0.05 |  |  | 23.7% | 0.05 | 27.0% | 0.05 |
|  | **Moderate pain** | 13.3% | 0.04 | 11.3% | 0.04 |  |  | 10.5% | 0.04 | 16.2% | 0.04 |
|  | **Severe pain** | 1.3% | 0.01 | 1.4% | 0.01 |  |  | 2.6% | 0.02 | 0.0% | 0.00 |
|  | **Extreme pain** | 0.0% | 0.00 | 0.0% | 0.00 |  |  | 0.0% | 0.00 | 0.0% | 0.00 |
| **40-65**  **(n = 878)** | **No pain** | 65.1% | 0.02 | 66.2% | 0.02 | 59.7% | 0.02 | 67.8% | 0.02 | 60.0% | 0.02 |
|  | **Slight pain** | 24.1% | 0.01 | 24.4% | 0.01 | 23.0% | 0.01 | 21.6% | 0.01 | 29.2% | 0.02 |
|  | **Moderate pain** | 7.6% | 0.01 | 7.2% | 0.01 | 10.1% | 0.01 | 7.0% | 0.01 | 8.8% | 0.01 |
|  | **Severe pain** | 2.8% | 0.01 | 2.3% | 0.01 | 5.8% | 0.01 | 3.3% | 0.01 | 2.0% | 0.00 |
|  | **Extreme pain** | 0.2% | 0.00 | 0.0% | 0.00 | 1.4% | 0.00 | 0.3% | 0.00 | 0.0% | 0.00 |
| **>65**  **(n = 320)** | **No pain** | 58.8% | 0.03 | 62.0% | 0.03 | 53.0% | 0.03 | 59.3% | 0.03 | 58.0% | 0.03 |
|  | **Slight pain** | 20.9% | 0.02 | 21.0% | 0.02 | 20.9% | 0.02 | 20.9% | 0.02 | 21.0% | 0.02 |
|  | **Moderate pain** | 14.1% | 0.02 | 11.7% | 0.02 | 18.3% | 0.02 | 15.8% | 0.02 | 11.9% | 0.02 |
|  | **Severe pain** | 6.3% | 0.01 | 5.4% | 0.01 | 7.8% | 0.02 | 4.0% | 0.01 | 9.1% | 0.02 |
|  | **Extreme pain** | 0.0% | 0.00 | 0.0% | 0.00 | 0.0% | 0.00 | 0.0% | 0.00 | 0.0% | 0.00 |
| **Anxiety/Depression** | |  |  |  |  |  |  |  |  |  |  |
| **<40**  **(n = 75)** | **Not anxious** | 42.7% | 0.06 | 42.3% | 0.06 |  |  | 34.2% | 0.05 | 51.4% | 0.06 |
|  | **Slightly anxious** | 25.3% | 0.05 | 26.8% | 0.05 |  |  | 28.9% | 0.05 | 21.6% | 0.05 |
|  | **Moderately anxious** | 26.7% | 0.05 | 25.4% | 0.05 |  |  | 28.9% | 0.05 | 24.3% | 0.05 |
|  | **Severely anxious** | 5.3% | 0.03 | 5.6% | 0.03 |  |  | 7.9% | 0.03 | 2.7% | 0.02 |
|  | **Extremely anxious** | 0.0% | 0.00 | 0.0% | 0.00 |  |  | 0.0% | 0.00 | 0.0% | 0.00 |
| **40-65**  **(n = 878)** | **Not anxious** | 36.6% | 0.02 | 36.8% | 0.02 | 35.3% | 0.02 | 34.6% | 0.02 | 40.3% | 0.02 |
|  | **Slightly anxious** | 33.0% | 0.02 | 33.6% | 0.02 | 30.2% | 0.02 | 33.8% | 0.02 | 31.5% | 0.02 |
|  | **Moderately anxious** | 21.3% | 0.01 | 20.6% | 0.01 | 25.2% | 0.01 | 22.0% | 0.01 | 20.0% | 0.01 |
|  | **Severely anxious** | 8.2% | 0.01 | 8.1% | 0.01 | 8.6% | 0.01 | 8.6% | 0.01 | 7.5% | 0.01 |
|  | **Extremely anxious** | 0.9% | 0.00 | 0.9% | 0.00 | 0.7% | 0.00 | 1.0% | 0.00 | 0.7% | 0.00 |
| **>65**  **(n = 319)** | **Not anxious** | 33.9% | 0.03 | 36.3% | 0.03 | 29.6% | 0.03 | 38.4% | 0.03 | 28.2% | 0.03 |
|  | **Slightly anxious** | 37.9% | 0.03 | 35.3% | 0.03 | 42.6% | 0.03 | 39.0% | 0.03 | 36.6% | 0.03 |
|  | **Moderately anxious** | 19.4% | 0.02 | 20.6% | 0.02 | 17.4% | 0.02 | 14.7% | 0.02 | 25.4% | 0.02 |
|  | **Severely anxious** | 8.5% | 0.02 | 7.4% | 0.01 | 10.4% | 0.02 | 7.3% | 0.01 | 9.9% | 0.02 |
|  | **Extremely anxious** | 0.3% | 0.00 | 0.5% | 0.00 | 0.0% | 0.00 | 0.6% | 0.00 | 0.0% | 0.00 |

* Percentages were not calculated for subgroups lower than 20 participants.

**Abbreviations. SE**: Standard Error.

# Supplementary table 1.2.1. Reference norms at diagnosis for the Summary score of EORTC QLQ-C30.

|  |  | **EORTC QLQ-C30: Summary score** | | | | |
| --- | --- | --- | --- | --- | --- | --- |
|  |  |  | **Charlson** | | **TNM stage** | |
|  |  | **All** | **0** | **≥ 1** | **0 – I** | **II - III** |
| **Age (years)**  **<40** | **n** | 75 | 71 | 4* | 37 | 38 |
|  | **Mean (SD)** | 85.7 (13.0) | 86.7 (11.6) | 67.1 (22.6) | 86.0 (11.6) | 85.4 (14.3) |
|  | **95%CI** | [ 82.8 - 88.6 ] | [ 84.0 - 89.4 ] | [ 44.9 - 89.2 ] | [ 82.3 - 89.8 ] | [ 80.8 - 89.9 ] |
|  | **P 5** | 56.3 | 58.8 |  | 57.6 | 53.1 |
|  | **P 10** | 68.2 | 69.1 |  | 69.1 | 58.2 |
|  | **P 20** | 78.2 | 78.8 |  | 79.0 | 76.3 |
|  | **P 25** | 79.9 | 81.0 |  | 82.2 | 78.0 |
|  | **P 30** | 83.3 | 83.7 |  | 83.3 | 82.7 |
|  | **P 40** | 85.4 | 85.6 |  | 85.3 | 86.2 |
|  | **Median** | 89.7 | 90.4 | 69.8 | 89.2 | 90.1 |
|  | **P 60** | 91.9 | 92.1 |  | 91.4 | 92.3 |
|  | **P 70** | 93.9 | 94.5 |  | 92.4 | 95.1 |
|  | **P 75** | 95.1 | 95.3 |  | 93.4 | 95.7 |
|  | **P 80** | 96.8 | 96.8 |  | 95.3 | 97.1 |
|  | **P 90** | 98.6 | 98.7 |  | 97.7 | 98.7 |
|  | **P 95** | 98.8 | 99.0 |  | 98.8 | 99.4 |
| **40-65** | **n** | 857 | 719 | 138 | 568 | 289 |
|  | **Mean (SD)** | 86.9 (11.2) | 87.1 (10.9) | 85.5 (12.5) | 87.1 (11.1) | 86.5 (11.4) |
|  | **95%CI** | [ 86.1 - 87.6 ] | [ 86.3 - 87.9 ] | [ 83.4 - 87.6 ] | [ 86.2 - 88.0 ] | [ 85.1 - 87.8 ] |
|  | **P 5** | 66.1 | 67.5 | 57.9 | 66.0 | 64.5 |
|  | **P 10** | 72.9 | 73.6 | 71.2 | 72.8 | 73.9 |
|  | **P 20** | 79.4 | 79.4 | 79.3 | 79.7 | 78.8 |
|  | **P 25** | 81.4 | 81.8 | 80.9 | 81.8 | 80.5 |
|  | **P 30** | 83.3 | 83.6 | 81.7 | 84.0 | 82.5 |
|  | **P 40** | 87.2 | 87.4 | 85.8 | 87.4 | 85.9 |
|  | **Median** | 89.7 | 89.7 | 88.8 | 89.8 | 89.3 |
|  | **P 60** | 91.7 | 92.1 | 91.2 | 91.8 | 91.7 |
|  | **P 70** | 94.2 | 94.2 | 93.6 | 94.2 | 93.6 |
|  | **P 75** | 94.9 | 95.2 | 94.3 | 94.9 | 94.9 |
|  | **P 80** | 95.8 | 96.2 | 95.0 | 95.7 | 96.2 |
|  | **P 90** | 98.1 | 98.1 | 97.5 | 98.1 | 98.1 |
|  | **P 95** | 98.7 | 98.7 | 99.4 | 98.7 | 99.3 |
| **>65** | **n** | 312 | 201 | 111 | 172 | 140 |
|  | **Mean (SD)** | 86.2 (12.2) | 87.4 (10.8) | 84.1 (14.3) | 86.8 (12.7) | 85.5 (11.5) |
|  | **95%CI** | [ 84.9 - 87.6 ] | [ 85.9 - 88.9 ] | [ 81.5 - 86.8 ] | [ 84.9 - 88.7 ] | [ 83.6 - 87.4 ] |
|  | **P 5** | 62.2 | 65.1 | 50.7 | 62.7 | 61.3 |
|  | **P 10** | 70.0 | 72.2 | 65.7 | 69.5 | 69.8 |
|  | **P 20** | 78.8 | 80.6 | 77.1 | 80.2 | 77.9 |
|  | **P 25** | 82.0 | 83.4 | 79.0 | 83.5 | 80.4 |
|  | **P 30** | 83.8 | 84.8 | 80.8 | 84.6 | 82.0 |
|  | **P 40** | 86.6 | 87.5 | 84.9 | 87.2 | 85.1 |
|  | **Median** | 89.3 | 90.2 | 87.2 | 90.6 | 87.7 |
|  | **P 60** | 91.6 | 91.8 | 90.7 | 92.3 | 90.5 |
|  | **P 70** | 93.8 | 94.0 | 93.0 | 93.9 | 92.9 |
|  | **P 75** | 94.9 | 95.0 | 94.4 | 94.9 | 94.8 |
|  | **P 80** | 95.7 | 95.9 | 95.3 | 96.2 | 95.5 |
|  | **P 90** | 98.2 | 98.7 | 97.6 | 98.7 | 97.4 |
|  | **P 95** | 99.4 | 99.5 | 98.8 | 99.6 | 98.7 |

* Deciles were not calculated for subgroups lower than 20 participants.

**Abbreviations**. **SD**: Standard Deviation; **95% CI**: 95% Confidence Interval; **P**: Percentile.

# Supplementary table 1.2.2. Reference norms at diagnosis for the Physical function of EORTC QLQ-C30.

|  |  | **EORTC QLQ-C30: Physical function** | | | | |
| --- | --- | --- | --- | --- | --- | --- |
|  |  |  | **Charlson** | | **TNM stage** | |
|  |  | **All** | **0** | **≥ 1** | **0 – I** | **II - III** |
| **Age (years)**  **<40** | **n** | 76 | 72 | 4* | 38 | 38 |
|  | **Mean (SD)** | 96.9 (7.0) | 97.4 (6.3) | 88.3 (13.7) | 97.9 (5.8) | 96.0 (8.0) |
|  | **95%CI** | [ 95.4 - 98.5 ] | [ 96.0 - 98.9 ] | [ 74.9 - 101.8 ] | [ 96.0 - 99.7 ] | [ 93.4 - 98.5 ] |
|  | **P 5** | 79.0 | 80.0 |  | 79.7 | 73.3 |
|  | **P 10** | 84.7 | 88.7 |  | 92.7 | 80.0 |
|  | **P 20** | 93.3 | 97.3 |  | 100.0 | 93.3 |
|  | **P 25** | 100.0 | 100.0 |  | 100.0 | 93.3 |
|  | **P 30** | 100.0 | 100.0 |  | 100.0 | 100.0 |
|  | **P 40** | 100.0 | 100.0 |  | 100.0 | 100.0 |
|  | **Median** | 100.0 | 100.0 | 90.0 | 100.0 | 100.0 |
|  | **P 60** | 100.0 | 100.0 |  | 100.0 | 100.0 |
|  | **P 70** | 100.0 | 100.0 |  | 100.0 | 100.0 |
|  | **P 75** | 100.0 | 100.0 |  | 100.0 | 100.0 |
|  | **P 80** | 100.0 | 100.0 |  | 100.0 | 100.0 |
|  | **P 90** | 100.0 | 100.0 |  | 100.0 | 100.0 |
|  | **P 95** | 100.0 | 100.0 |  | 100.0 | 100.0 |
| **40-65** | **n** | 872 | 733 | 139 | 578 | 294 |
|  | **Mean (SD)** | 94.5 (11.4) | 95.2 (10.1) | 90.6 (15.9) | 94.7 (11.1) | 94.0 (11.9) |
|  | **95%CI** | [ 93.7 - 95.2 ] | [ 94.5 - 95.9 ] | [ 88.0 - 93.3 ] | [ 93.8 - 95.6 ] | [ 92.7 - 95.4 ] |
|  | **P 5** | 73.3 | 80.0 | 60.0 | 73.3 | 71.7 |
|  | **P 10** | 80.0 | 86.7 | 73.3 | 86.7 | 80.0 |
|  | **P 20** | 89.7 | 93.3 | 86.7 | 93.3 | 86.7 |
|  | **P 25** | 93.3 | 93.3 | 86.7 | 93.3 | 93.3 |
|  | **P 30** | 93.3 | 93.3 | 86.7 | 93.3 | 93.3 |
|  | **P 40** | 100.0 | 100.0 | 93.3 | 100.0 | 100.0 |
|  | **Median** | 100.0 | 100.0 | 100.0 | 100.0 | 100.0 |
|  | **P 60** | 100.0 | 100.0 | 100.0 | 100.0 | 100.0 |
|  | **P 70** | 100.0 | 100.0 | 100.0 | 100.0 | 100.0 |
|  | **P 75** | 100.0 | 100.0 | 100.0 | 100.0 | 100.0 |
|  | **P 80** | 100.0 | 100.0 | 100.0 | 100.0 | 100.0 |
|  | **P 90** | 100.0 | 100.0 | 100.0 | 100.0 | 100.0 |
|  | **P 95** | 100.0 | 100.0 | 100.0 | 100.0 | 100.0 |
| **>65** | **n** | 321 | 205 | 116 | 177 | 144 |
|  | **Mean (SD)** | 86.6 (17.8) | 90.2 (14.6) | 80.3 (21.0) | 88.4 (16.5) | 84.5 (19.1) |
|  | **95%CI** | [ 84.7 - 88.6 ] | [ 88.2 - 92.2 ] | [ 76.5 - 84.2 ] | [ 86.0 - 90.8 ] | [ 81.3 - 87.6 ] |
|  | **P 5** | 53.3 | 55.3 | 26.7 | 53.3 | 35.0 |
|  | **P 10** | 61.3 | 66.7 | 53.3 | 66.7 | 56.7 |
|  | **P 20** | 76.0 | 80.7 | 66.7 | 80.0 | 73.3 |
|  | **P 25** | 80.0 | 86.7 | 66.7 | 80.0 | 80.0 |
|  | **P 30** | 86.7 | 86.7 | 73.3 | 86.7 | 80.0 |
|  | **P 40** | 86.7 | 93.3 | 80.0 | 93.3 | 86.7 |
|  | **Median** | 93.3 | 93.3 | 86.7 | 93.3 | 93.3 |
|  | **P 60** | 100.0 | 100.0 | 93.3 | 100.0 | 93.3 |
|  | **P 70** | 100.0 | 100.0 | 93.3 | 100.0 | 100.0 |
|  | **P 75** | 100.0 | 100.0 | 98.3 | 100.0 | 100.0 |
|  | **P 80** | 100.0 | 100.0 | 100.0 | 100.0 | 100.0 |
|  | **P 90** | 100.0 | 100.0 | 100.0 | 100.0 | 100.0 |
|  | **P 95** | 100.0 | 100.0 | 100.0 | 100.0 | 100.0 |

* Deciles were not calculated for subgroups lower than 20 participants.

**Abbreviations**. **SD**: Standard Deviation; **95% CI**: 95% Confidence Interval; **P**: Percentile.

# Supplementary table 1.2.3. Reference norms at diagnosis for the Role function of EORTC QLQ-C30.

|  |  | **EORTC QLQ-C30: Role function** | | | | |
| --- | --- | --- | --- | --- | --- | --- |
|  |  |  | **Charlson** | | **TNM stage** | |
|  |  | **All** | **0** | **≥ 1** | **0 – I** | **II - III** |
| **Age (years)**  **<40** | **n** | 76 | 72 | 4* | 38 | 38 |
|  | **Mean (SD)** | 89.5 (21.7) | 89.8 (21.2) | 83.3 (33.3) | 91.2 (20.4) | 87.7 (23.1) |
|  | **95%CI** | [ 84.6 - 94.4 ] | [ 84.9 - 94.7 ] | [ 50.7 - 116.0 ] | [ 84.7 - 97.7 ] | [ 80.4 - 95.1 ] |
|  | **P 5** | 33.3 | 33.3 |  | 47.5 | 32.5 |
|  | **P 10** | 66.7 | 66.7 |  | 66.7 | 33.3 |
|  | **P 20** | 73.3 | 76.7 |  | 80.0 | 66.7 |
|  | **P 25** | 87.5 | 87.5 |  | 100.0 | 83.3 |
|  | **P 30** | 100.0 | 100.0 |  | 100.0 | 95.0 |
|  | **P 40** | 100.0 | 100.0 |  | 100.0 | 100.0 |
|  | **Median** | 100.0 | 100.0 | 100.0 | 100.0 | 100.0 |
|  | **P 60** | 100.0 | 100.0 |  | 100.0 | 100.0 |
|  | **P 70** | 100.0 | 100.0 |  | 100.0 | 100.0 |
|  | **P 75** | 100.0 | 100.0 |  | 100.0 | 100.0 |
|  | **P 80** | 100.0 | 100.0 |  | 100.0 | 100.0 |
|  | **P 90** | 100.0 | 100.0 |  | 100.0 | 100.0 |
|  | **P 95** | 100.0 | 100.0 |  | 100.0 | 100.0 |
| **40-65** | **n** | 874 | 735 | 139 | 579 | 295 |
|  | **Mean (SD)** | 91.7 (19.1) | 91.7 (18.8) | 91.8 (20.4) | 92.2 (18.2) | 90.8 (20.6) |
|  | **95%CI** | [ 90.4 - 93.0 ] | [ 90.3 - 93.0 ] | [ 88.5 - 95.2 ] | [ 90.7 - 93.7 ] | [ 88.4 - 93.1 ] |
|  | **P 5** | 50.0 | 50.0 | 33.3 | 50.0 | 33.3 |
|  | **P 10** | 66.7 | 66.7 | 66.7 | 66.7 | 66.7 |
|  | **P 20** | 83.3 | 83.3 | 83.3 | 83.3 | 83.3 |
|  | **P 25** | 100.0 | 100.0 | 100.0 | 100.0 | 100.0 |
|  | **P 30** | 100.0 | 100.0 | 100.0 | 100.0 | 100.0 |
|  | **P 40** | 100.0 | 100.0 | 100.0 | 100.0 | 100.0 |
|  | **Median** | 100.0 | 100.0 | 100.0 | 100.0 | 100.0 |
|  | **P 60** | 100.0 | 100.0 | 100.0 | 100.0 | 100.0 |
|  | **P 70** | 100.0 | 100.0 | 100.0 | 100.0 | 100.0 |
|  | **P 75** | 100.0 | 100.0 | 100.0 | 100.0 | 100.0 |
|  | **P 80** | 100.0 | 100.0 | 100.0 | 100.0 | 100.0 |
|  | **P 90** | 100.0 | 100.0 | 100.0 | 100.0 | 100.0 |
|  | **P 95** | 100.0 | 100.0 | 100.0 | 100.0 | 100.0 |
| **>65** | **n** | 319 | 205 | 114 | 176 | 143 |
|  | **Mean (SD)** | 91.0 (20.9) | 92.2 (18.2) | 88.9 (25.0) | 91.2 (20.8) | 90.8 (21.1) |
|  | **95%CI** | [ 88.7 - 93.3 ] | [ 89.7 - 94.7 ] | [ 84.3 - 93.5 ] | [ 88.1 - 94.3 ] | [ 87.3 - 94.2 ] |
|  | **P 5** | 33.3 | 50.0 | 25.0 | 33.3 | 33.3 |
|  | **P 10** | 66.7 | 66.7 | 66.7 | 66.7 | 66.7 |
|  | **P 20** | 83.3 | 83.3 | 83.3 | 83.3 | 83.3 |
|  | **P 25** | 100.0 | 100.0 | 100.0 | 100.0 | 100.0 |
|  | **P 30** | 100.0 | 100.0 | 100.0 | 100.0 | 100.0 |
|  | **P 40** | 100.0 | 100.0 | 100.0 | 100.0 | 100.0 |
|  | **Median** | 100.0 | 100.0 | 100.0 | 100.0 | 100.0 |
|  | **P 60** | 100.0 | 100.0 | 100.0 | 100.0 | 100.0 |
|  | **P 70** | 100.0 | 100.0 | 100.0 | 100.0 | 100.0 |
|  | **P 75** | 100.0 | 100.0 | 100.0 | 100.0 | 100.0 |
|  | **P 80** | 100.0 | 100.0 | 100.0 | 100.0 | 100.0 |
|  | **P 90** | 100.0 | 100.0 | 100.0 | 100.0 | 100.0 |
|  | **P 95** | 100.0 | 100.0 | 100.0 | 100.0 | 100.0 |

* Deciles were not calculated for subgroups lower than 20 participants.

**Abbreviations**. **SD**: Standard Deviation; **95% CI**: 95% Confidence Interval; **P**: Percentile.

# Supplementary table 1.2.4. Reference norms at diagnosis for the Emotional function of EORTC QLQ-C30.

|  |  | **EORTC QLQ-C30: Emotional function** | | | | |
| --- | --- | --- | --- | --- | --- | --- |
|  |  |  | **Charlson** | | **TNM stage** | |
|  |  | **All** | **0** | **≥ 1** | **0 – I** | **II - III** |
| **Age (years)**  **<40** | **n** | 76 | 72 | 4* | 38 | 38 |
|  | **Mean (SD)** | 60.0 (22.6) | 61.0 (22.3) | 41.7 (22.6) | 57.9 (20.3) | 62.1 (24.7) |
|  | **95%CI** | [ 54.9 - 65.1 ] | [ 55.8 - 66.1 ] | [ 19.6 - 63.8 ] | [ 51.4 - 64.4 ] | [ 54.2 - 69.9 ] |
|  | **P 5** | 23.8 | 22.1 |  | 23.8 | 15.8 |
|  | **P 10** | 33.3 | 35.8 |  | 33.3 | 25.0 |
|  | **P 20** | 41.7 | 41.7 |  | 41.7 | 41.7 |
|  | **P 25** | 43.8 | 50.0 |  | 41.7 | 47.9 |
|  | **P 30** | 50.0 | 50.0 |  | 50.0 | 50.0 |
|  | **P 40** | 56.7 | 58.3 |  | 55.0 | 55.0 |
|  | **Median** | 58.3 | 58.3 | 33.3 | 58.3 | 58.3 |
|  | **P 60** | 66.7 | 66.7 |  | 58.3 | 75.0 |
|  | **P 70** | 75.0 | 75.0 |  | 66.7 | 77.5 |
|  | **P 75** | 75.0 | 75.0 |  | 68.8 | 83.3 |
|  | **P 80** | 83.3 | 83.3 |  | 75.0 | 85.0 |
|  | **P 90** | 91.7 | 91.7 |  | 83.3 | 92.5 |
|  | **P 95** | 100.0 | 100.0 |  | 100.0 | 100.0 |
| **40-65** | **n** | 876 | 737 | 139 | 582 | 294 |
|  | **Mean (SD)** | 65.5 (23.3) | 65.9 (22.7) | 63.6 (25.8) | 65.2 (23.6) | 66.1 (22.5) |
|  | **95%CI** | [ 64.0 - 67.1 ] | [ 64.2 - 67.5 ] | [ 59.3 - 67.9 ] | [ 63.3 - 67.1 ] | [ 63.5 - 68.7 ] |
|  | **P 5** | 16.7 | 25.0 | 8.3 | 16.7 | 25.0 |
|  | **P 10** | 33.3 | 33.3 | 16.7 | 33.3 | 33.3 |
|  | **P 20** | 50.0 | 50.0 | 50.0 | 50.0 | 50.0 |
|  | **P 25** | 50.0 | 50.0 | 50.0 | 50.0 | 56.3 |
|  | **P 30** | 58.3 | 58.3 | 58.3 | 58.3 | 58.3 |
|  | **P 40** | 66.7 | 66.7 | 66.7 | 66.7 | 66.7 |
|  | **Median** | 66.7 | 66.7 | 66.7 | 66.7 | 66.7 |
|  | **P 60** | 75.0 | 75.0 | 66.7 | 75.0 | 75.0 |
|  | **P 70** | 83.3 | 83.3 | 83.3 | 83.3 | 83.3 |
|  | **P 75** | 83.3 | 83.3 | 83.3 | 83.3 | 83.3 |
|  | **P 80** | 83.3 | 83.3 | 83.3 | 83.3 | 83.3 |
|  | **P 90** | 91.7 | 91.7 | 100.0 | 91.7 | 91.7 |
|  | **P 95** | 100.0 | 100.0 | 100.0 | 100.0 | 100.0 |
| **>65** | **n** | 320 | 205 | 115 | 177 | 143 |
|  | **Mean (SD)** | 67.4 (23.5) | 67.8 (23.8) | 66.7 (23.0) | 69.4 (23.4) | 64.9 (23.4) |
|  | **95%CI** | [ 64.8 - 70.0 ] | [ 64.5 - 71.0 ] | [ 62.5 - 70.9 ] | [ 65.9 - 72.8 ] | [ 61.1 - 68.7 ] |
|  | **P 5** | 25.0 | 19.2 | 25.0 | 24.2 | 25.0 |
|  | **P 10** | 33.3 | 33.3 | 33.3 | 33.3 | 33.3 |
|  | **P 20** | 50.0 | 50.0 | 50.0 | 50.0 | 50.0 |
|  | **P 25** | 50.0 | 50.0 | 58.3 | 58.3 | 50.0 |
|  | **P 30** | 58.3 | 58.3 | 58.3 | 58.3 | 50.0 |
|  | **P 40** | 66.7 | 66.7 | 66.7 | 66.7 | 58.3 |
|  | **Median** | 75.0 | 75.0 | 75.0 | 75.0 | 66.7 |
|  | **P 60** | 75.0 | 75.0 | 75.0 | 75.0 | 75.0 |
|  | **P 70** | 83.3 | 83.3 | 75.0 | 83.3 | 75.0 |
|  | **P 75** | 83.3 | 83.3 | 83.3 | 83.3 | 83.3 |
|  | **P 80** | 91.7 | 91.7 | 83.3 | 91.7 | 83.3 |
|  | **P 90** | 100.0 | 100.0 | 100.0 | 100.0 | 100.0 |
|  | **P 95** | 100.0 | 100.0 | 100.0 | 100.0 | 100.0 |

* Deciles were not calculated for subgroups lower than 20 participants.

**Abbreviations**. **SD**: Standard Deviation; **95% CI**: 95% Confidence Interval; **P**: Percentile.

# Supplementary table 1.2.5. Reference norms at diagnosis for the Cognitive function of EORTC QLQ-C30.

|  |  | **EORTC QLQ-C30: Cognitive function** | | | | |
| --- | --- | --- | --- | --- | --- | --- |
|  |  |  | **Charlson** | | **TNM stage** | |
|  |  | **All** | **0** | **≥ 1** | **0 – I** | **II - III** |
| **Age (years)**  **<40** | **n** | 76 | 72 | 4* | 38 | 38 |
|  | **Mean (SD)** | 86.0 (17.4) | 86.3 (17.1) | 79.2 (25.0) | 86.4 (17.7) | 85.5 (17.4) |
|  | **95%CI** | [ 82.0 - 89.9 ] | [ 82.4 - 90.3 ] | [ 54.7 - 103.7 ] | [ 80.8 - 92.0 ] | [ 80.0 - 91.1 ] |
|  | **P 5** | 50.0 | 50.0 |  | 50.0 | 50.0 |
|  | **P 10** | 50.0 | 55.0 |  | 50.0 | 50.0 |
|  | **P 20** | 66.7 | 66.7 |  | 66.7 | 66.7 |
|  | **P 25** | 66.7 | 70.8 |  | 66.7 | 66.7 |
|  | **P 30** | 83.3 | 83.3 |  | 83.3 | 83.3 |
|  | **P 40** | 83.3 | 83.3 |  | 83.3 | 83.3 |
|  | **Median** | 100.0 | 100.0 | 83.3 | 100.0 | 91.7 |
|  | **P 60** | 100.0 | 100.0 |  | 100.0 | 100.0 |
|  | **P 70** | 100.0 | 100.0 |  | 100.0 | 100.0 |
|  | **P 75** | 100.0 | 100.0 |  | 100.0 | 100.0 |
|  | **P 80** | 100.0 | 100.0 |  | 100.0 | 100.0 |
|  | **P 90** | 100.0 | 100.0 |  | 100.0 | 100.0 |
|  | **P 95** | 100.0 | 100.0 |  | 100.0 | 100.0 |
| **40-65** | **n** | 875 | 736 | 139 | 582 | 293 |
|  | **Mean (SD)** | 85.3 (20.7) | 85.4 (20.6) | 84.9 (21.3) | 85.1 (20.7) | 85.7 (20.7) |
|  | **95%CI** | [ 83.9 - 86.7 ] | [ 83.9 - 86.9 ] | [ 81.3 - 88.4 ] | [ 83.4 - 86.8 ] | [ 83.3 - 88.1 ] |
|  | **P 5** | 33.3 | 47.5 | 33.3 | 33.3 | 45.0 |
|  | **P 10** | 66.7 | 66.7 | 50.0 | 50.0 | 66.7 |
|  | **P 20** | 66.7 | 66.7 | 66.7 | 66.7 | 66.7 |
|  | **P 25** | 83.3 | 83.3 | 83.3 | 83.3 | 83.3 |
|  | **P 30** | 83.3 | 83.3 | 83.3 | 83.3 | 83.3 |
|  | **P 40** | 83.3 | 83.3 | 83.3 | 83.3 | 83.3 |
|  | **Median** | 100.0 | 100.0 | 100.0 | 100.0 | 100.0 |
|  | **P 60** | 100.0 | 100.0 | 100.0 | 100.0 | 100.0 |
|  | **P 70** | 100.0 | 100.0 | 100.0 | 100.0 | 100.0 |
|  | **P 75** | 100.0 | 100.0 | 100.0 | 100.0 | 100.0 |
|  | **P 80** | 100.0 | 100.0 | 100.0 | 100.0 | 100.0 |
|  | **P 90** | 100.0 | 100.0 | 100.0 | 100.0 | 100.0 |
|  | **P 95** | 100.0 | 100.0 | 100.0 | 100.0 | 100.0 |
| **>65** | **n** | 320 | 205 | 115 | 177 | 143 |
|  | **Mean (SD)** | 86.2 (20.0) | 87.6 (18.3) | 83.8 (22.6) | 86.3 (19.0) | 86.0 (21.3) |
|  | **95%CI** | [ 84.0 - 88.4 ] | [ 85.1 - 90.1 ] | [ 79.6 - 87.9 ] | [ 83.6 - 89.1 ] | [ 82.5 - 89.5 ] |
|  | **P 5** | 50.0 | 50.0 | 33.3 | 33.3 | 50.0 |
|  | **P 10** | 66.7 | 66.7 | 50.0 | 66.7 | 50.0 |
|  | **P 20** | 66.7 | 66.7 | 66.7 | 66.7 | 66.7 |
|  | **P 25** | 83.3 | 83.3 | 66.7 | 83.3 | 83.3 |
|  | **P 30** | 83.3 | 83.3 | 83.3 | 83.3 | 83.3 |
|  | **P 40** | 83.3 | 83.3 | 83.3 | 83.3 | 83.3 |
|  | **Median** | 100.0 | 100.0 | 100.0 | 100.0 | 100.0 |
|  | **P 60** | 100.0 | 100.0 | 100.0 | 100.0 | 100.0 |
|  | **P 70** | 100.0 | 100.0 | 100.0 | 100.0 | 100.0 |
|  | **P 75** | 100.0 | 100.0 | 100.0 | 100.0 | 100.0 |
|  | **P 80** | 100.0 | 100.0 | 100.0 | 100.0 | 100.0 |
|  | **P 90** | 100.0 | 100.0 | 100.0 | 100.0 | 100.0 |
|  | **P 95** | 100.0 | 100.0 | 100.0 | 100.0 | 100.0 |

* Deciles were not calculated for subgroups lower than 20 participants.

**Abbreviations**. **SD**: Standard Deviation; **95% CI**: 95% Confidence Interval; **P**: Percentile.

# Supplementary table 1.2.6. Reference norms at diagnosis for the Social function of EORTC QLQ-C30.

|  |  | **EORTC QLQ-C30: Social function** | | | | |
| --- | --- | --- | --- | --- | --- | --- |
|  |  |  | **Charlson** | | **TNM stage** | |
|  |  | **All** | **0** | **≥ 1** | **0 – I** | **II - III** |
| **Age (years)**  **<40** | **n** | 76 | 72 | 4* | 38 | 38 |
|  | **Mean (SD)** | 81.8 (27.4) | 82.6 (27.2) | 66.7 (30.4) | 82.9 (25.3) | 80.7 (29.6) |
|  | **95%CI** | [ 75.6 - 88.0 ] | [ 76.4 - 88.9 ] | [ 36.8 - 96.5 ] | [ 74.9 - 90.9 ] | [ 71.3 - 90.1 ] |
|  | **P 5** | 28.3 | 21.7 |  | 31.7 | 0.0 |
|  | **P 10** | 33.3 | 33.3 |  | 33.3 | 33.3 |
|  | **P 20** | 66.7 | 66.7 |  | 66.7 | 50.0 |
|  | **P 25** | 66.7 | 70.8 |  | 66.7 | 66.7 |
|  | **P 30** | 83.3 | 83.3 |  | 83.3 | 83.3 |
|  | **P 40** | 83.3 | 83.3 |  | 83.3 | 83.3 |
|  | **Median** | 100.0 | 100.0 | 66.7 | 100.0 | 100.0 |
|  | **P 60** | 100.0 | 100.0 |  | 100.0 | 100.0 |
|  | **P 70** | 100.0 | 100.0 |  | 100.0 | 100.0 |
|  | **P 75** | 100.0 | 100.0 |  | 100.0 | 100.0 |
|  | **P 80** | 100.0 | 100.0 |  | 100.0 | 100.0 |
|  | **P 90** | 100.0 | 100.0 |  | 100.0 | 100.0 |
|  | **P 95** | 100.0 | 100.0 |  | 100.0 | 100.0 |
| **40-65** | **n** | 872 | 733 | 139 | 579 | 293 |
|  | **Mean (SD)** | 88.4 (21.2) | 88.4 (21.1) | 88.1 (21.9) | 89.4 (20.1) | 86.2 (23.0) |
|  | **95%CI** | [ 87.0 - 89.8 ] | [ 86.9 - 89.9 ] | [ 84.5 - 91.8 ] | [ 87.8 - 91.1 ] | [ 83.6 - 88.9 ] |
|  | **P 5** | 50.0 | 50.0 | 33.3 | 50.0 | 33.3 |
|  | **P 10** | 66.7 | 66.7 | 50.0 | 66.7 | 50.0 |
|  | **P 20** | 66.7 | 80.0 | 66.7 | 83.3 | 66.7 |
|  | **P 25** | 83.3 | 83.3 | 83.3 | 83.3 | 83.3 |
|  | **P 30** | 83.3 | 83.3 | 83.3 | 100.0 | 83.3 |
|  | **P 40** | 100.0 | 100.0 | 100.0 | 100.0 | 100.0 |
|  | **Median** | 100.0 | 100.0 | 100.0 | 100.0 | 100.0 |
|  | **P 60** | 100.0 | 100.0 | 100.0 | 100.0 | 100.0 |
|  | **P 70** | 100.0 | 100.0 | 100.0 | 100.0 | 100.0 |
|  | **P 75** | 100.0 | 100.0 | 100.0 | 100.0 | 100.0 |
|  | **P 80** | 100.0 | 100.0 | 100.0 | 100.0 | 100.0 |
|  | **P 90** | 100.0 | 100.0 | 100.0 | 100.0 | 100.0 |
|  | **P 95** | 100.0 | 100.0 | 100.0 | 100.0 | 100.0 |
| **>65** | **n** | 318 | 203 | 115 | 176 | 142 |
|  | **Mean (SD)** | 92.4 (17.7) | 94.0 (15.7) | 89.6 (20.6) | 92.6 (17.2) | 92.1 (18.4) |
|  | **95%CI** | [ 90.5 - 94.3 ] | [ 91.8 - 96.2 ] | [ 85.8 - 93.3 ] | [ 90.1 - 95.2 ] | [ 89.1 - 95.2 ] |
|  | **P 5** | 50.0 | 66.7 | 33.3 | 50.0 | 52.5 |
|  | **P 10** | 66.7 | 66.7 | 60.0 | 66.7 | 66.7 |
|  | **P 20** | 83.3 | 100.0 | 83.3 | 83.3 | 83.3 |
|  | **P 25** | 100.0 | 100.0 | 83.3 | 100.0 | 100.0 |
|  | **P 30** | 100.0 | 100.0 | 100.0 | 100.0 | 100.0 |
|  | **P 40** | 100.0 | 100.0 | 100.0 | 100.0 | 100.0 |
|  | **Median** | 100.0 | 100.0 | 100.0 | 100.0 | 100.0 |
|  | **P 60** | 100.0 | 100.0 | 100.0 | 100.0 | 100.0 |
|  | **P 70** | 100.0 | 100.0 | 100.0 | 100.0 | 100.0 |
|  | **P 75** | 100.0 | 100.0 | 100.0 | 100.0 | 100.0 |
|  | **P 80** | 100.0 | 100.0 | 100.0 | 100.0 | 100.0 |
|  | **P 90** | 100.0 | 100.0 | 100.0 | 100.0 | 100.0 |
|  | **P 95** | 100.0 | 100.0 | 100.0 | 100.0 | 100.0 |

* Deciles were not calculated for subgroups lower than 20 participants.

**Abbreviations**. **SD**: Standard Deviation; **95% CI**: 95% Confidence Interval; **P**: Percentile.

# Supplementary table 1.2.7. Reference norms at diagnosis for the Global health status of EORTC QLQ-C30.

|  |  | **EORTC QLQ-C30: Global health status** | | | | |
| --- | --- | --- | --- | --- | --- | --- |
|  |  |  | **Charlson** | | **TNM stage** | |
|  |  | **All** | **0** | **≥ 1** | **0 – I** | **II - III** |
| **Age (years)**  **<40** | **n** | 76 | 72 | 4* | 38 | 38 |
|  | **Mean (SD)** | 72.8 (18.9) | 73.3 (18.5) | 64.6 (27.5) | 74.6 (18.5) | 71.1 (19.4) |
|  | **95%CI** | [ 68.6 - 77.1 ] | [ 69.0 - 77.5 ] | [ 37.6 - 91.6 ] | [ 68.7 - 80.4 ] | [ 64.9 - 77.2 ] |
|  | **P 5** | 33.3 | 38.8 |  | 33.3 | 33.3 |
|  | **P 10** | 47.5 | 50.0 |  | 50.0 | 41.7 |
|  | **P 20** | 53.3 | 58.3 |  | 56.7 | 50.0 |
|  | **P 25** | 58.3 | 58.3 |  | 64.6 | 58.3 |
|  | **P 30** | 66.7 | 66.7 |  | 66.7 | 58.3 |
|  | **P 40** | 66.7 | 66.7 |  | 71.7 | 66.7 |
|  | **Median** | 75.0 | 75.0 | 66.7 | 79.2 | 75.0 |
|  | **P 60** | 83.3 | 83.3 |  | 83.3 | 83.3 |
|  | **P 70** | 83.3 | 83.3 |  | 83.3 | 83.3 |
|  | **P 75** | 83.3 | 83.3 |  | 83.3 | 83.3 |
|  | **P 80** | 83.3 | 83.3 |  | 91.7 | 83.3 |
|  | **P 90** | 100.0 | 100.0 |  | 100.0 | 100.0 |
|  | **P 95** | 100.0 | 100.0 |  | 100.0 | 100.0 |
| **40-65** | **n** | 877 | 738 | 139 | 582 | 295 |
|  | **Mean (SD)** | 73.9 (19.3) | 74.9 (18.4) | 68.6 (22.8) | 74.5 (19.2) | 72.6 (19.4) |
|  | **95%CI** | [ 72.6 - 75.2 ] | [ 73.6 - 76.2 ] | [ 64.8 - 72.4 ] | [ 73.0 - 76.1 ] | [ 70.4 - 74.8 ] |
|  | **P 5** | 41.7 | 41.7 | 25.0 | 41.7 | 41.7 |
|  | **P 10** | 50.0 | 50.0 | 33.3 | 50.0 | 50.0 |
|  | **P 20** | 58.3 | 58.3 | 50.0 | 58.3 | 58.3 |
|  | **P 25** | 66.7 | 66.7 | 58.3 | 66.7 | 66.7 |
|  | **P 30** | 66.7 | 66.7 | 58.3 | 66.7 | 66.7 |
|  | **P 40** | 66.7 | 75.0 | 66.7 | 66.7 | 66.7 |
|  | **Median** | 75.0 | 83.3 | 66.7 | 83.3 | 75.0 |
|  | **P 60** | 83.3 | 83.3 | 75.0 | 83.3 | 83.3 |
|  | **P 70** | 83.3 | 83.3 | 83.3 | 83.3 | 83.3 |
|  | **P 75** | 83.3 | 83.3 | 83.3 | 83.3 | 83.3 |
|  | **P 80** | 91.7 | 91.7 | 83.3 | 91.7 | 83.3 |
|  | **P 90** | 100.0 | 100.0 | 100.0 | 100.0 | 100.0 |
|  | **P 95** | 100.0 | 100.0 | 100.0 | 100.0 | 100.0 |
| **>65** | **n** | 321 | 205 | 116 | 177 | 144 |
|  | **Mean (SD)** | 72.6 (20.8) | 74.6 (19.9) | 69.0 (21.8) | 73.5 (19.5) | 71.4 (22.2) |
|  | **95%CI** | [ 70.3 - 74.8 ] | [ 71.8 - 77.3 ] | [ 65.1 - 73.0 ] | [ 70.7 - 76.4 ] | [ 67.7 - 75.0 ] |
|  | **P 5** | 33.3 | 33.3 | 23.8 | 33.3 | 33.3 |
|  | **P 10** | 50.0 | 50.0 | 47.5 | 50.0 | 37.5 |
|  | **P 20** | 58.3 | 60.0 | 50.0 | 63.3 | 50.0 |
|  | **P 25** | 66.7 | 66.7 | 50.0 | 66.7 | 58.3 |
|  | **P 30** | 66.7 | 66.7 | 58.3 | 66.7 | 62.5 |
|  | **P 40** | 66.7 | 75.0 | 66.7 | 66.7 | 66.7 |
|  | **Median** | 75.0 | 83.3 | 66.7 | 75.0 | 75.0 |
|  | **P 60** | 83.3 | 83.3 | 76.7 | 83.3 | 83.3 |
|  | **P 70** | 83.3 | 83.3 | 83.3 | 83.3 | 83.3 |
|  | **P 75** | 83.3 | 83.3 | 83.3 | 83.3 | 83.3 |
|  | **P 80** | 88.3 | 91.7 | 83.3 | 86.7 | 91.7 |
|  | **P 90** | 100.0 | 100.0 | 100.0 | 100.0 | 100.0 |
|  | **P 95** | 100.0 | 100.0 | 100.0 | 100.0 | 100.0 |

* Deciles were not calculated for subgroups lower than 20 participants.

**Abbreviations**. **SD**: Standard Deviation; **95% CI**: 95% Confidence Interval; **P**: Percentile.

# Supplementary table 1.2.8. Reference norms at diagnosis for the Fatigue of EORTC QLQ-C30.

|  |  | **EORTC QLQ-C30: Fatigue** | | | | |
| --- | --- | --- | --- | --- | --- | --- |
|  |  |  | **Charlson** | | **TNM stage** | |
|  |  | **All** | **0** | **≥ 1** | **0 – I** | **II - III** |
| **Age (years)**  **<40** | **n** | 76 | 72 | 4* | 38 | 38 |
|  | **Mean (SD)** | 16.1 (21.3) | 14.7 (18.3) | 41.7 (50.0) | 11.7 (17.3) | 20.5 (24.0) |
|  | **95%CI** | [ 11.3 - 20.9 ] | [ 10.4 - 18.9 ] | [ -7.3 - 90.7 ] | [ 6.2 - 17.2 ] | [ 12.8 - 28.1 ] |
|  | **P 5** | 0.0 | 0.0 |  | 0.0 | 0.0 |
|  | **P 10** | 0.0 | 0.0 |  | 0.0 | 0.0 |
|  | **P 20** | 0.0 | 0.0 |  | 0.0 | 0.0 |
|  | **P 25** | 0.0 | 0.0 |  | 0.0 | 0.0 |
|  | **P 30** | 0.0 | 0.0 |  | 0.0 | 0.0 |
|  | **P 40** | 0.0 | 0.0 |  | 0.0 | 11.1 |
|  | **Median** | 11.1 | 11.1 | 33.3 | 0.0 | 11.1 |
|  | **P 60** | 11.1 | 11.1 |  | 11.1 | 22.2 |
|  | **P 70** | 22.2 | 22.2 |  | 14.4 | 25.6 |
|  | **P 75** | 22.2 | 22.2 |  | 22.2 | 33.3 |
|  | **P 80** | 33.3 | 33.3 |  | 24.4 | 33.3 |
|  | **P 90** | 47.8 | 41.1 |  | 35.6 | 56.7 |
|  | **P 95** | 57.2 | 55.6 |  | 55.6 | 78.9 |
| **40-65** | **n** | 873 | 734 | 139 | 579 | 294 |
|  | **Mean (SD)** | 15.0 (19.3) | 14.6 (18.9) | 17.1 (21.5) | 14.4 (18.6) | 16.1 (20.7) |
|  | **95%CI** | [ 13.7 - 16.2 ] | [ 13.2 - 15.9 ] | [ 13.5 - 20.7 ] | [ 12.9 - 15.9 ] | [ 13.8 - 18.5 ] |
|  | **P 5** | 0.0 | 0.0 | 0.0 | 0.0 | 0.0 |
|  | **P 10** | 0.0 | 0.0 | 0.0 | 0.0 | 0.0 |
|  | **P 20** | 0.0 | 0.0 | 0.0 | 0.0 | 0.0 |
|  | **P 25** | 0.0 | 0.0 | 0.0 | 0.0 | 0.0 |
|  | **P 30** | 0.0 | 0.0 | 0.0 | 0.0 | 0.0 |
|  | **P 40** | 0.0 | 0.0 | 0.0 | 0.0 | 0.0 |
|  | **Median** | 11.1 | 11.1 | 11.1 | 11.1 | 11.1 |
|  | **P 60** | 11.1 | 11.1 | 11.1 | 11.1 | 11.1 |
|  | **P 70** | 22.2 | 22.2 | 22.2 | 22.2 | 22.2 |
|  | **P 75** | 22.2 | 22.2 | 33.3 | 22.2 | 33.3 |
|  | **P 80** | 33.3 | 33.3 | 33.3 | 33.3 | 33.3 |
|  | **P 90** | 44.4 | 44.4 | 44.4 | 44.4 | 44.4 |
|  | **P 95** | 55.6 | 55.6 | 66.7 | 55.6 | 58.3 |
| **>65** | **n** | 320 | 205 | 115 | 177 | 143 |
|  | **Mean (SD)** | 18.2 (20.5) | 15.9 (17.5) | 22.4 (24.5) | 16.8 (20.3) | 20.0 (20.7) |
|  | **95%CI** | [ 16.0 - 20.5 ] | [ 13.5 - 18.3 ] | [ 17.9 - 26.9 ] | [ 13.8 - 19.8 ] | [ 16.6 - 23.4 ] |
|  | **P 5** | 0.0 | 0.0 | 0.0 | 0.0 | 0.0 |
|  | **P 10** | 0.0 | 0.0 | 0.0 | 0.0 | 0.0 |
|  | **P 20** | 0.0 | 0.0 | 0.0 | 0.0 | 0.0 |
|  | **P 25** | 0.0 | 0.0 | 0.0 | 0.0 | 0.0 |
|  | **P 30** | 0.0 | 0.0 | 0.0 | 0.0 | 0.0 |
|  | **P 40** | 11.1 | 11.1 | 11.1 | 0.0 | 11.1 |
|  | **Median** | 11.1 | 11.1 | 16.7 | 11.1 | 11.1 |
|  | **P 60** | 22.2 | 22.2 | 22.2 | 16.7 | 22.2 |
|  | **P 70** | 22.2 | 22.2 | 33.3 | 22.2 | 33.3 |
|  | **P 75** | 33.3 | 22.2 | 33.3 | 22.2 | 33.3 |
|  | **P 80** | 33.3 | 33.3 | 42.2 | 33.3 | 33.3 |
|  | **P 90** | 44.4 | 33.3 | 66.7 | 44.4 | 44.4 |
|  | **P 95** | 66.7 | 52.2 | 68.9 | 66.7 | 66.7 |

* Deciles were not calculated for subgroups lower than 20 participants.

**Abbreviations**. **SD**: Standard Deviation; **95% CI**: 95% Confidence Interval; **P**: Percentile.

# Supplementary table 1.2.9. Reference norms at diagnosis for the Nausea of EORTC QLQ-C30.

|  |  | **EORTC QLQ-C30: Nausea** | | | | |
| --- | --- | --- | --- | --- | --- | --- |
|  |  |  | **Charlson** | | **TNM stage** | |
|  |  | **All** | **0** | **≥ 1** | **0 – I** | **II - III** |
| **Age (years)**  **<40** | **n** | 76 | 72 | 4* | 38 | 38 |
|  | **Mean (SD)** | 2.9 (9.6) | 3.0 (9.8) | 0.0 (0.0) | 3.5 (11.7) | 2.2 (6.9) |
|  | **95%CI** | [ 0.7 - 5.0 ] | [ 0.7 - 5.3 ] | [ 0.0 - 0.0 ] | [ -0.2 - 7.2 ] | [ 0.0 - 4.4 ] |
|  | **P 5** | 0.0 | 0.0 |  | 0.0 | 0.0 |
|  | **P 10** | 0.0 | 0.0 |  | 0.0 | 0.0 |
|  | **P 20** | 0.0 | 0.0 |  | 0.0 | 0.0 |
|  | **P 25** | 0.0 | 0.0 |  | 0.0 | 0.0 |
|  | **P 30** | 0.0 | 0.0 |  | 0.0 | 0.0 |
|  | **P 40** | 0.0 | 0.0 |  | 0.0 | 0.0 |
|  | **Median** | 0.0 | 0.0 | 0.0 | 0.0 | 0.0 |
|  | **P 60** | 0.0 | 0.0 |  | 0.0 | 0.0 |
|  | **P 70** | 0.0 | 0.0 |  | 0.0 | 0.0 |
|  | **P 75** | 0.0 | 0.0 |  | 0.0 | 0.0 |
|  | **P 80** | 0.0 | 0.0 |  | 0.0 | 0.0 |
|  | **P 90** | 16.7 | 16.7 |  | 16.7 | 16.7 |
|  | **P 95** | 16.7 | 16.7 |  | 19.2 | 17.5 |
| **40-65** | **n** | 873 | 735 | 138 | 579 | 294 |
|  | **Mean (SD)** | 2.9 (10.2) | 2.8 (9.7) | 3.5 (12.6) | 2.5 (9.0) | 3.9 (12.3) |
|  | **95%CI** | [ 2.3 - 3.6 ] | [ 2.1 - 3.5 ] | [ 1.4 - 5.6 ] | [ 1.7 - 3.2 ] | [ 2.4 - 5.3 ] |
|  | **P 5** | 0.0 | 0.0 | 0.0 | 0.0 | 0.0 |
|  | **P 10** | 0.0 | 0.0 | 0.0 | 0.0 | 0.0 |
|  | **P 20** | 0.0 | 0.0 | 0.0 | 0.0 | 0.0 |
|  | **P 25** | 0.0 | 0.0 | 0.0 | 0.0 | 0.0 |
|  | **P 30** | 0.0 | 0.0 | 0.0 | 0.0 | 0.0 |
|  | **P 40** | 0.0 | 0.0 | 0.0 | 0.0 | 0.0 |
|  | **Median** | 0.0 | 0.0 | 0.0 | 0.0 | 0.0 |
|  | **P 60** | 0.0 | 0.0 | 0.0 | 0.0 | 0.0 |
|  | **P 70** | 0.0 | 0.0 | 0.0 | 0.0 | 0.0 |
|  | **P 75** | 0.0 | 0.0 | 0.0 | 0.0 | 0.0 |
|  | **P 80** | 0.0 | 0.0 | 0.0 | 0.0 | 0.0 |
|  | **P 90** | 16.7 | 16.7 | 16.7 | 0.0 | 16.7 |
|  | **P 95** | 16.7 | 33.3 | 16.7 | 16.7 | 33.3 |
| **>65** | **n** | 319 | 205 | 114 | 176 | 143 |
|  | **Mean (SD)** | 1.9 (8.9) | 1.7 (8.0) | 2.2 (10.3) | 2.5 (9.8) | 1.2 (7.6) |
|  | **95%CI** | [ 0.9 - 2.9 ] | [ 0.6 - 2.8 ] | [ 0.3 - 4.1 ] | [ 1.0 - 3.9 ] | [ -0.1 - 2.4 ] |
|  | **P 5** | 0.0 | 0.0 | 0.0 | 0.0 | 0.0 |
|  | **P 10** | 0.0 | 0.0 | 0.0 | 0.0 | 0.0 |
|  | **P 20** | 0.0 | 0.0 | 0.0 | 0.0 | 0.0 |
|  | **P 25** | 0.0 | 0.0 | 0.0 | 0.0 | 0.0 |
|  | **P 30** | 0.0 | 0.0 | 0.0 | 0.0 | 0.0 |
|  | **P 40** | 0.0 | 0.0 | 0.0 | 0.0 | 0.0 |
|  | **Median** | 0.0 | 0.0 | 0.0 | 0.0 | 0.0 |
|  | **P 60** | 0.0 | 0.0 | 0.0 | 0.0 | 0.0 |
|  | **P 70** | 0.0 | 0.0 | 0.0 | 0.0 | 0.0 |
|  | **P 75** | 0.0 | 0.0 | 0.0 | 0.0 | 0.0 |
|  | **P 80** | 0.0 | 0.0 | 0.0 | 0.0 | 0.0 |
|  | **P 90** | 0.0 | 0.0 | 0.0 | 0.0 | 0.0 |
|  | **P 95** | 16.7 | 16.7 | 20.8 | 33.3 | 0.0 |

* Deciles were not calculated for subgroups lower than 20 participants.

**Abbreviations**. **SD**: Standard Deviation; **95% CI**: 95% Confidence Interval; **P**: Percentile.

# Supplementary table 1.2.10. Reference norms at diagnosis for the Pain of EORTC QLQ-C30.

|  |  | **EORTC QLQ-C30: Pain** | | | | |
| --- | --- | --- | --- | --- | --- | --- |
|  |  |  | **Charlson** | | **TNM stage** | |
|  |  | **All** | **0** | **≥ 1** | **0 – I** | **II - III** |
| **Age (years)**  **<40** | **n** | 76 | 72 | 4* | 38 | 38 |
|  | **Mean (SD)** | 13.6 (20.7) | 12.3 (19.2) | 37.5 (34.4) | 11.8 (20.5) | 15.4 (21.0) |
|  | **95%CI** | [ 8.9 - 18.2 ] | [ 7.8 - 16.7 ] | [ 3.8 - 71.2 ] | [ 5.3 - 18.4 ] | [ 8.7 - 22.0 ] |
|  | **P 5** | 0.0 | 0.0 |  | 0.0 | 0.0 |
|  | **P 10** | 0.0 | 0.0 |  | 0.0 | 0.0 |
|  | **P 20** | 0.0 | 0.0 |  | 0.0 | 0.0 |
|  | **P 25** | 0.0 | 0.0 |  | 0.0 | 0.0 |
|  | **P 30** | 0.0 | 0.0 |  | 0.0 | 0.0 |
|  | **P 40** | 0.0 | 0.0 |  | 0.0 | 0.0 |
|  | **Median** | 0.0 | 0.0 | 41.7 | 0.0 | 8.3 |
|  | **P 60** | 16.7 | 16.7 |  | 0.0 | 16.7 |
|  | **P 70** | 16.7 | 16.7 |  | 16.7 | 16.7 |
|  | **P 75** | 16.7 | 16.7 |  | 16.7 | 16.7 |
|  | **P 80** | 26.7 | 16.7 |  | 33.3 | 20.0 |
|  | **P 90** | 50.0 | 33.3 |  | 33.3 | 51.7 |
|  | **P 95** | 66.7 | 50.0 |  | 52.5 | 66.7 |
| **40-65** | **n** | 876 | 737 | 139 | 582 | 294 |
|  | **Mean (SD)** | 11.7 (19.4) | 11.4 (19.1) | 13.1 (20.8) | 11.1 (19.3) | 12.8 (19.7) |
|  | **95%CI** | [ 10.4 - 13.0 ] | [ 10.1 - 12.8 ] | [ 9.6 - 16.5 ] | [ 9.6 - 12.7 ] | [ 10.6 - 15.1 ] |
|  | **P 5** | 0.0 | 0.0 | 0.0 | 0.0 | 0.0 |
|  | **P 10** | 0.0 | 0.0 | 0.0 | 0.0 | 0.0 |
|  | **P 20** | 0.0 | 0.0 | 0.0 | 0.0 | 0.0 |
|  | **P 25** | 0.0 | 0.0 | 0.0 | 0.0 | 0.0 |
|  | **P 30** | 0.0 | 0.0 | 0.0 | 0.0 | 0.0 |
|  | **P 40** | 0.0 | 0.0 | 0.0 | 0.0 | 0.0 |
|  | **Median** | 0.0 | 0.0 | 0.0 | 0.0 | 0.0 |
|  | **P 60** | 0.0 | 0.0 | 0.0 | 0.0 | 0.0 |
|  | **P 70** | 16.7 | 16.7 | 16.7 | 16.7 | 16.7 |
|  | **P 75** | 16.7 | 16.7 | 16.7 | 16.7 | 16.7 |
|  | **P 80** | 16.7 | 16.7 | 33.3 | 16.7 | 33.3 |
|  | **P 90** | 33.3 | 33.3 | 50.0 | 33.3 | 33.3 |
|  | **P 95** | 50.0 | 50.0 | 66.7 | 50.0 | 50.0 |
| **>65** | **n** | 319 | 205 | 114 | 176 | 143 |
|  | **Mean (SD)** | 16.2 (23.3) | 15.3 (21.7) | 18.0 (26.0) | 15.7 (21.4) | 16.9 (25.5) |
|  | **95%CI** | [ 13.7 - 18.8 ] | [ 12.3 - 18.3 ] | [ 13.2 - 22.7 ] | [ 12.6 - 18.9 ] | [ 12.7 - 21.1 ] |
|  | **P 5** | 0.0 | 0.0 | 0.0 | 0.0 | 0.0 |
|  | **P 10** | 0.0 | 0.0 | 0.0 | 0.0 | 0.0 |
|  | **P 20** | 0.0 | 0.0 | 0.0 | 0.0 | 0.0 |
|  | **P 25** | 0.0 | 0.0 | 0.0 | 0.0 | 0.0 |
|  | **P 30** | 0.0 | 0.0 | 0.0 | 0.0 | 0.0 |
|  | **P 40** | 0.0 | 0.0 | 0.0 | 0.0 | 0.0 |
|  | **Median** | 0.0 | 0.0 | 0.0 | 0.0 | 0.0 |
|  | **P 60** | 16.7 | 16.7 | 16.7 | 16.7 | 16.7 |
|  | **P 70** | 16.7 | 16.7 | 16.7 | 16.7 | 16.7 |
|  | **P 75** | 33.3 | 25.0 | 33.3 | 33.3 | 33.3 |
|  | **P 80** | 33.3 | 33.3 | 33.3 | 33.3 | 33.3 |
|  | **P 90** | 50.0 | 50.0 | 58.3 | 50.0 | 50.0 |
|  | **P 95** | 66.7 | 66.7 | 70.8 | 66.7 | 66.7 |

* Deciles were not calculated for subgroups lower than 20 participants.

**Abbreviations**. **SD**: Standard Deviation; **95% CI**: 95% Confidence Interval; **P**: Percentile.

# Supplementary table 1.2.11. Reference norms at diagnosis for the Dyspnea of EORTC QLQ-C30.

|  |  | **EORTC QLQ-C30: Dyspnea** | | | | |
| --- | --- | --- | --- | --- | --- | --- |
|  |  |  | **Charlson** | | **TNM stage** | |
|  |  | **All** | **0** | **≥ 1** | **0 – I** | **II - III** |
| **Age (years)**  **<40** | **n** | 76 | 72 | 4* | 38 | 38 |
|  | **Mean (SD)** | 6.1 (14.1) | 5.6 (12.5) | 16.7 (33.3) | 6.1 (13.1) | 6.1 (15.2) |
|  | **95%CI** | [ 3.0 - 9.3 ] | [ 2.7 - 8.4 ] | [ -16.0 - 49.3 ] | [ 2.0 - 10.3 ] | [ 1.3 - 11.0 ] |
|  | **P 5** | 0.0 | 0.0 |  | 0.0 | 0.0 |
|  | **P 10** | 0.0 | 0.0 |  | 0.0 | 0.0 |
|  | **P 20** | 0.0 | 0.0 |  | 0.0 | 0.0 |
|  | **P 25** | 0.0 | 0.0 |  | 0.0 | 0.0 |
|  | **P 30** | 0.0 | 0.0 |  | 0.0 | 0.0 |
|  | **P 40** | 0.0 | 0.0 |  | 0.0 | 0.0 |
|  | **Median** | 0.0 | 0.0 | 0.0 | 0.0 | 0.0 |
|  | **P 60** | 0.0 | 0.0 |  | 0.0 | 0.0 |
|  | **P 70** | 0.0 | 0.0 |  | 0.0 | 0.0 |
|  | **P 75** | 0.0 | 0.0 |  | 0.0 | 0.0 |
|  | **P 80** | 0.0 | 0.0 |  | 6.7 | 0.0 |
|  | **P 90** | 33.3 | 33.3 |  | 33.3 | 33.3 |
|  | **P 95** | 33.3 | 33.3 |  | 33.3 | 35.0 |
| **40-65** | **n** | 871 | 732 | 139 | 578 | 293 |
|  | **Mean (SD)** | 9.5 (20.1) | 9.5 (20.1) | 9.6 (19.8) | 9.8 (20.6) | 8.9 (19.0) |
|  | **95%CI** | [ 8.2 - 10.8 ] | [ 8.0 - 10.9 ] | [ 6.3 - 12.9 ] | [ 8.1 - 11.5 ] | [ 6.7 - 11.1 ] |
|  | **P 5** | 0.0 | 0.0 | 0.0 | 0.0 | 0.0 |
|  | **P 10** | 0.0 | 0.0 | 0.0 | 0.0 | 0.0 |
|  | **P 20** | 0.0 | 0.0 | 0.0 | 0.0 | 0.0 |
|  | **P 25** | 0.0 | 0.0 | 0.0 | 0.0 | 0.0 |
|  | **P 30** | 0.0 | 0.0 | 0.0 | 0.0 | 0.0 |
|  | **P 40** | 0.0 | 0.0 | 0.0 | 0.0 | 0.0 |
|  | **Median** | 0.0 | 0.0 | 0.0 | 0.0 | 0.0 |
|  | **P 60** | 0.0 | 0.0 | 0.0 | 0.0 | 0.0 |
|  | **P 70** | 0.0 | 0.0 | 0.0 | 0.0 | 0.0 |
|  | **P 75** | 0.0 | 0.0 | 0.0 | 0.0 | 0.0 |
|  | **P 80** | 33.3 | 33.3 | 33.3 | 33.3 | 33.3 |
|  | **P 90** | 33.3 | 33.3 | 33.3 | 33.3 | 33.3 |
|  | **P 95** | 66.7 | 66.7 | 66.7 | 66.7 | 33.3 |
| **>65** | **n** | 320 | 206 | 114 | 176 | 144 |
|  | **Mean (SD)** | 10.0 (20.7) | 9.9 (19.6) | 10.2 (22.7) | 9.1 (19.0) | 11.1 (22.6) |
|  | **95%CI** | [ 7.7 - 12.3 ] | [ 7.2 - 12.5 ] | [ 6.1 - 14.4 ] | [ 6.3 - 11.9 ] | [ 7.4 - 14.8 ] |
|  | **P 5** | 0.0 | 0.0 | 0.0 | 0.0 | 0.0 |
|  | **P 10** | 0.0 | 0.0 | 0.0 | 0.0 | 0.0 |
|  | **P 20** | 0.0 | 0.0 | 0.0 | 0.0 | 0.0 |
|  | **P 25** | 0.0 | 0.0 | 0.0 | 0.0 | 0.0 |
|  | **P 30** | 0.0 | 0.0 | 0.0 | 0.0 | 0.0 |
|  | **P 40** | 0.0 | 0.0 | 0.0 | 0.0 | 0.0 |
|  | **Median** | 0.0 | 0.0 | 0.0 | 0.0 | 0.0 |
|  | **P 60** | 0.0 | 0.0 | 0.0 | 0.0 | 0.0 |
|  | **P 70** | 0.0 | 0.0 | 0.0 | 0.0 | 0.0 |
|  | **P 75** | 0.0 | 0.0 | 0.0 | 0.0 | 0.0 |
|  | **P 80** | 33.3 | 33.3 | 33.3 | 33.3 | 33.3 |
|  | **P 90** | 33.3 | 33.3 | 33.3 | 33.3 | 33.3 |
|  | **P 95** | 33.3 | 33.3 | 66.7 | 33.3 | 66.7 |

* Deciles were not calculated for subgroups lower than 20 participants.

**Abbreviations**. **SD**: Standard Deviation; **95% CI**: 95% Confidence Interval; **P**: Percentile.

# Supplementary table 1.2.12. Reference norms at diagnosis for the Insomnia of EORTC QLQ-C30.

|  |  | **EORTC QLQ-C30: Insomnia** | | | | |
| --- | --- | --- | --- | --- | --- | --- |
|  |  |  | **Charlson** | | **TNM stage** | |
|  |  | **All** | **0** | **≥ 1** | **0 – I** | **II - III** |
| **Age (years)**  **<40** | **n** | 76 | 72 | 4* | 38 | 38 |
|  | **Mean (SD)** | 29.4 (31.7) | 27.8 (30.6) | 58.3 (41.9) | 33.3 (32.0) | 25.4 (31.4) |
|  | **95%CI** | [ 22.3 - 36.5 ] | [ 20.7 - 34.9 ] | [ 17.2 - 99.4 ] | [ 23.2 - 43.5 ] | [ 15.4 - 35.4 ] |
|  | **P 5** | 0.0 | 0.0 |  | 0.0 | 0.0 |
|  | **P 10** | 0.0 | 0.0 |  | 0.0 | 0.0 |
|  | **P 20** | 0.0 | 0.0 |  | 0.0 | 0.0 |
|  | **P 25** | 0.0 | 0.0 |  | 0.0 | 0.0 |
|  | **P 30** | 0.0 | 0.0 |  | 0.0 | 0.0 |
|  | **P 40** | 0.0 | 0.0 |  | 33.3 | 0.0 |
|  | **Median** | 33.3 | 33.3 | 66.7 | 33.3 | 16.7 |
|  | **P 60** | 33.3 | 33.3 |  | 33.3 | 33.3 |
|  | **P 70** | 33.3 | 33.3 |  | 43.3 | 33.3 |
|  | **P 75** | 33.3 | 33.3 |  | 66.7 | 33.3 |
|  | **P 80** | 66.7 | 66.7 |  | 66.7 | 40.0 |
|  | **P 90** | 66.7 | 66.7 |  | 70.0 | 70.0 |
|  | **P 95** | 100.0 | 100.0 |  | 100.0 | 100.0 |
| **40-65** | **n** | 874 | 735 | 139 | 580 | 294 |
|  | **Mean (SD)** | 32.5 (30.9) | 32.1 (30.2) | 34.8 (34.3) | 33.1 (31.4) | 31.3 (29.8) |
|  | **95%CI** | [ 30.4 - 34.5 ] | [ 29.9 - 34.2 ] | [ 29.1 - 40.5 ] | [ 30.5 - 35.7 ] | [ 27.9 - 34.7 ] |
|  | **P 5** | 0.0 | 0.0 | 0.0 | 0.0 | 0.0 |
|  | **P 10** | 0.0 | 0.0 | 0.0 | 0.0 | 0.0 |
|  | **P 20** | 0.0 | 0.0 | 0.0 | 0.0 | 0.0 |
|  | **P 25** | 0.0 | 0.0 | 0.0 | 0.0 | 0.0 |
|  | **P 30** | 0.0 | 0.0 | 0.0 | 0.0 | 0.0 |
|  | **P 40** | 33.3 | 33.3 | 0.0 | 33.3 | 33.3 |
|  | **Median** | 33.3 | 33.3 | 33.3 | 33.3 | 33.3 |
|  | **P 60** | 33.3 | 33.3 | 33.3 | 33.3 | 33.3 |
|  | **P 70** | 33.3 | 33.3 | 66.7 | 66.7 | 33.3 |
|  | **P 75** | 66.7 | 66.7 | 66.7 | 66.7 | 66.7 |
|  | **P 80** | 66.7 | 66.7 | 66.7 | 66.7 | 66.7 |
|  | **P 90** | 66.7 | 66.7 | 100.0 | 66.7 | 66.7 |
|  | **P 95** | 100.0 | 100.0 | 100.0 | 100.0 | 75.0 |
| **>65** | **n** | 321 | 206 | 115 | 177 | 144 |
|  | **Mean (SD)** | 31.2 (31.5) | 31.6 (31.4) | 30.4 (31.7) | 30.7 (30.2) | 31.7 (33.1) |
|  | **95%CI** | [ 27.7 - 34.6 ] | [ 27.3 - 35.8 ] | [ 24.6 - 36.2 ] | [ 26.2 - 35.2 ] | [ 26.3 - 37.1 ] |
|  | **P 5** | 0.0 | 0.0 | 0.0 | 0.0 | 0.0 |
|  | **P 10** | 0.0 | 0.0 | 0.0 | 0.0 | 0.0 |
|  | **P 20** | 0.0 | 0.0 | 0.0 | 0.0 | 0.0 |
|  | **P 25** | 0.0 | 0.0 | 0.0 | 0.0 | 0.0 |
|  | **P 30** | 0.0 | 0.0 | 0.0 | 0.0 | 0.0 |
|  | **P 40** | 0.0 | 33.3 | 0.0 | 33.3 | 0.0 |
|  | **Median** | 33.3 | 33.3 | 33.3 | 33.3 | 33.3 |
|  | **P 60** | 33.3 | 33.3 | 33.3 | 33.3 | 33.3 |
|  | **P 70** | 33.3 | 33.3 | 66.7 | 33.3 | 50.0 |
|  | **P 75** | 66.7 | 66.7 | 66.7 | 66.7 | 66.7 |
|  | **P 80** | 66.7 | 66.7 | 66.7 | 66.7 | 66.7 |
|  | **P 90** | 66.7 | 66.7 | 66.7 | 66.7 | 66.7 |
|  | **P 95** | 100.0 | 100.0 | 100.0 | 100.0 | 100.0 |

* Deciles were not calculated for subgroups lower than 20 participants.

**Abbreviations**. **SD**: Standard Deviation; **95% CI**: 95% Confidence Interval; **P**: Percentile.

# Supplementary table 1.2.13. Reference norms at diagnosis for the Appetite loss of EORTC QLQ-C30.

|  |  | **EORTC QLQ-C30: Appetite loss** | | | | |
| --- | --- | --- | --- | --- | --- | --- |
|  |  |  | **Charlson** | | **TNM stage** | |
|  |  | **All** | **0** | **≥ 1** | **0 – I** | **II - III** |
| **Age (years)**  **<40** | **n** | 75 | 71 | 4* | 37 | 38 |
|  | **Mean (SD)** | 17.8 (25.3) | 15.5 (23.1) | 58.3 (31.9) | 19.8 (24.2) | 15.8 (26.6) |
|  | **95%CI** | [ 12.0 - 23.5 ] | [ 10.1 - 20.9 ] | [ 27.1 - 89.6 ] | [ 12.0 - 27.6 ] | [ 7.3 - 24.2 ] |
|  | **P 5** | 0.0 | 0.0 |  | 0.0 | 0.0 |
|  | **P 10** | 0.0 | 0.0 |  | 0.0 | 0.0 |
|  | **P 20** | 0.0 | 0.0 |  | 0.0 | 0.0 |
|  | **P 25** | 0.0 | 0.0 |  | 0.0 | 0.0 |
|  | **P 30** | 0.0 | 0.0 |  | 0.0 | 0.0 |
|  | **P 40** | 0.0 | 0.0 |  | 0.0 | 0.0 |
|  | **Median** | 0.0 | 0.0 | 50.0 | 0.0 | 0.0 |
|  | **P 60** | 0.0 | 0.0 |  | 33.3 | 0.0 |
|  | **P 70** | 33.3 | 33.3 |  | 33.3 | 33.3 |
|  | **P 75** | 33.3 | 33.3 |  | 33.3 | 33.3 |
|  | **P 80** | 33.3 | 33.3 |  | 33.3 | 33.3 |
|  | **P 90** | 66.7 | 66.7 |  | 66.7 | 66.7 |
|  | **P 95** | 66.7 | 66.7 |  | 66.7 | 68.3 |
| **40-65** | **n** | 873 | 734 | 139 | 580 | 293 |
|  | **Mean (SD)** | 12.9 (22.7) | 12.9 (22.6) | 12.5 (22.8) | 12.0 (21.7) | 14.6 (24.4) |
|  | **95%CI** | [ 11.4 - 14.4 ] | [ 11.3 - 14.6 ] | [ 8.7 - 16.3 ] | [ 10.2 - 13.8 ] | [ 11.8 - 17.4 ] |
|  | **P 5** | 0.0 | 0.0 | 0.0 | 0.0 | 0.0 |
|  | **P 10** | 0.0 | 0.0 | 0.0 | 0.0 | 0.0 |
|  | **P 20** | 0.0 | 0.0 | 0.0 | 0.0 | 0.0 |
|  | **P 25** | 0.0 | 0.0 | 0.0 | 0.0 | 0.0 |
|  | **P 30** | 0.0 | 0.0 | 0.0 | 0.0 | 0.0 |
|  | **P 40** | 0.0 | 0.0 | 0.0 | 0.0 | 0.0 |
|  | **Median** | 0.0 | 0.0 | 0.0 | 0.0 | 0.0 |
|  | **P 60** | 0.0 | 0.0 | 0.0 | 0.0 | 0.0 |
|  | **P 70** | 0.0 | 0.0 | 0.0 | 0.0 | 33.3 |
|  | **P 75** | 33.3 | 33.3 | 33.3 | 33.3 | 33.3 |
|  | **P 80** | 33.3 | 33.3 | 33.3 | 33.3 | 33.3 |
|  | **P 90** | 33.3 | 33.3 | 33.3 | 33.3 | 66.7 |
|  | **P 95** | 66.7 | 66.7 | 66.7 | 66.7 | 66.7 |
| **>65** | **n** | 321 | 205 | 116 | 177 | 144 |
|  | **Mean (SD)** | 11.1 (21.2) | 9.1 (17.9) | 14.7 (25.7) | 10.0 (20.9) | 12.5 (21.5) |
|  | **95%CI** | [ 8.8 - 13.4 ] | [ 6.7 - 11.6 ] | [ 10.0 - 19.3 ] | [ 6.9 - 13.1 ] | [ 9.0 - 16.0 ] |
|  | **P 5** | 0.0 | 0.0 | 0.0 | 0.0 | 0.0 |
|  | **P 10** | 0.0 | 0.0 | 0.0 | 0.0 | 0.0 |
|  | **P 20** | 0.0 | 0.0 | 0.0 | 0.0 | 0.0 |
|  | **P 25** | 0.0 | 0.0 | 0.0 | 0.0 | 0.0 |
|  | **P 30** | 0.0 | 0.0 | 0.0 | 0.0 | 0.0 |
|  | **P 40** | 0.0 | 0.0 | 0.0 | 0.0 | 0.0 |
|  | **Median** | 0.0 | 0.0 | 0.0 | 0.0 | 0.0 |
|  | **P 60** | 0.0 | 0.0 | 0.0 | 0.0 | 0.0 |
|  | **P 70** | 0.0 | 0.0 | 30.0 | 0.0 | 16.7 |
|  | **P 75** | 33.3 | 0.0 | 33.3 | 0.0 | 33.3 |
|  | **P 80** | 33.3 | 33.3 | 33.3 | 33.3 | 33.3 |
|  | **P 90** | 33.3 | 33.3 | 66.7 | 33.3 | 33.3 |
|  | **P 95** | 66.7 | 33.3 | 66.7 | 66.7 | 66.7 |

* Deciles were not calculated for subgroups lower than 20 participants.

**Abbreviations**. **SD**: Standard Deviation; **95% CI**: 95% Confidence Interval; **P**: Percentile.

# Supplementary table 1.2.14. Reference norms at diagnosis for the Constipation of EORTC QLQ-C30.

|  |  | **EORTC QLQ-C30: Constipation** | | | | |
| --- | --- | --- | --- | --- | --- | --- |
|  |  |  | **Charlson** | | **TNM stage** | |
|  |  | **All** | **0** | **≥ 1** | **0 – I** | **II - III** |
| **Age (years)**  **<40** | **n** | 76 | 72 | 4* | 38 | 38 |
|  | **Mean (SD)** | 7.5 (18.5) | 5.6 (13.7) | 41.7 (50.0) | 4.4 (15.8) | 10.5 (20.7) |
|  | **95%CI** | [ 3.3 - 11.6 ] | [ 2.4 - 8.7 ] | [ -7.3 - 90.7 ] | [ -0.6 - 9.4 ] | [ 4.0 - 17.1 ] |
|  | **P 5** | 0.0 | 0.0 |  | 0.0 | 0.0 |
|  | **P 10** | 0.0 | 0.0 |  | 0.0 | 0.0 |
|  | **P 20** | 0.0 | 0.0 |  | 0.0 | 0.0 |
|  | **P 25** | 0.0 | 0.0 |  | 0.0 | 0.0 |
|  | **P 30** | 0.0 | 0.0 |  | 0.0 | 0.0 |
|  | **P 40** | 0.0 | 0.0 |  | 0.0 | 0.0 |
|  | **Median** | 0.0 | 0.0 | 33.3 | 0.0 | 0.0 |
|  | **P 60** | 0.0 | 0.0 |  | 0.0 | 0.0 |
|  | **P 70** | 0.0 | 0.0 |  | 0.0 | 0.0 |
|  | **P 75** | 0.0 | 0.0 |  | 0.0 | 33.3 |
|  | **P 80** | 0.0 | 0.0 |  | 0.0 | 33.3 |
|  | **P 90** | 33.3 | 33.3 |  | 3.3 | 33.3 |
|  | **P 95** | 38.3 | 33.3 |  | 66.7 | 36.7 |
| **40-65** | **n** | 872 | 733 | 139 | 579 | 293 |
|  | **Mean (SD)** | 9.0 (20.3) | 8.8 (19.8) | 10.3 (23.0) | 8.9 (20.4) | 9.2 (20.3) |
|  | **95%CI** | [ 7.7 - 10.4 ] | [ 7.3 - 10.2 ] | [ 6.5 - 14.1 ] | [ 7.3 - 10.6 ] | [ 6.9 - 11.5 ] |
|  | **P 5** | 0.0 | 0.0 | 0.0 | 0.0 | 0.0 |
|  | **P 10** | 0.0 | 0.0 | 0.0 | 0.0 | 0.0 |
|  | **P 20** | 0.0 | 0.0 | 0.0 | 0.0 | 0.0 |
|  | **P 25** | 0.0 | 0.0 | 0.0 | 0.0 | 0.0 |
|  | **P 30** | 0.0 | 0.0 | 0.0 | 0.0 | 0.0 |
|  | **P 40** | 0.0 | 0.0 | 0.0 | 0.0 | 0.0 |
|  | **Median** | 0.0 | 0.0 | 0.0 | 0.0 | 0.0 |
|  | **P 60** | 0.0 | 0.0 | 0.0 | 0.0 | 0.0 |
|  | **P 70** | 0.0 | 0.0 | 0.0 | 0.0 | 0.0 |
|  | **P 75** | 0.0 | 0.0 | 0.0 | 0.0 | 0.0 |
|  | **P 80** | 0.0 | 0.0 | 33.3 | 0.0 | 33.3 |
|  | **P 90** | 33.3 | 33.3 | 33.3 | 33.3 | 33.3 |
|  | **P 95** | 66.7 | 66.7 | 66.7 | 66.7 | 66.7 |
| **>65** | **n** | 318 | 204 | 114 | 176 | 142 |
|  | **Mean (SD)** | 12.6 (23.7) | 11.6 (22.5) | 14.3 (25.8) | 12.5 (23.5) | 12.7 (24.1) |
|  | **95%CI** | [ 10.0 - 15.2 ] | [ 8.5 - 14.7 ] | [ 9.6 - 19.1 ] | [ 9.0 - 16.0 ] | [ 8.7 - 16.6 ] |
|  | **P 5** | 0.0 | 0.0 | 0.0 | 0.0 | 0.0 |
|  | **P 10** | 0.0 | 0.0 | 0.0 | 0.0 | 0.0 |
|  | **P 20** | 0.0 | 0.0 | 0.0 | 0.0 | 0.0 |
|  | **P 25** | 0.0 | 0.0 | 0.0 | 0.0 | 0.0 |
|  | **P 30** | 0.0 | 0.0 | 0.0 | 0.0 | 0.0 |
|  | **P 40** | 0.0 | 0.0 | 0.0 | 0.0 | 0.0 |
|  | **Median** | 0.0 | 0.0 | 0.0 | 0.0 | 0.0 |
|  | **P 60** | 0.0 | 0.0 | 0.0 | 0.0 | 0.0 |
|  | **P 70** | 0.0 | 0.0 | 0.0 | 0.0 | 0.0 |
|  | **P 75** | 33.3 | 0.0 | 33.3 | 33.3 | 33.3 |
|  | **P 80** | 33.3 | 33.3 | 33.3 | 33.3 | 33.3 |
|  | **P 90** | 36.7 | 33.3 | 66.7 | 33.3 | 66.7 |
|  | **P 95** | 66.7 | 66.7 | 66.7 | 66.7 | 66.7 |

* Deciles were not calculated for subgroups lower than 20 participants.

**Abbreviations**. **SD**: Standard Deviation; **95% CI**: 95% Confidence Interval; **P**: Percentile.

# Supplementary table 1.2.15. Reference norms at diagnosis for the Diarrhea of EORTC QLQ-C30.

|  |  | **EORTC QLQ-C30: Diarrhea** | | | | |
| --- | --- | --- | --- | --- | --- | --- |
|  |  |  | **Charlson** | | **TNM stage** | |
|  |  | **All** | **0** | **≥ 1** | **0 – I** | **II - III** |
| **Age (years)**  **<40** | **n** | 76 | 72 | 4* | 38 | 38 |
|  | **Mean (SD)** | 4.8 (16.1) | 3.2 (11.4) | 33.3 (47.1) | 3.5 (12.9) | 6.1 (18.8) |
|  | **95%CI** | [ 1.2 - 8.4 ] | [ 0.6 - 5.9 ] | [ -12.9 - 79.5 ] | [ -0.6 - 7.6 ] | [ 0.2 - 12.1 ] |
|  | **P 5** | 0.0 | 0.0 |  | 0.0 | 0.0 |
|  | **P 10** | 0.0 | 0.0 |  | 0.0 | 0.0 |
|  | **P 20** | 0.0 | 0.0 |  | 0.0 | 0.0 |
|  | **P 25** | 0.0 | 0.0 |  | 0.0 | 0.0 |
|  | **P 30** | 0.0 | 0.0 |  | 0.0 | 0.0 |
|  | **P 40** | 0.0 | 0.0 |  | 0.0 | 0.0 |
|  | **Median** | 0.0 | 0.0 | 16.7 | 0.0 | 0.0 |
|  | **P 60** | 0.0 | 0.0 |  | 0.0 | 0.0 |
|  | **P 70** | 0.0 | 0.0 |  | 0.0 | 0.0 |
|  | **P 75** | 0.0 | 0.0 |  | 0.0 | 0.0 |
|  | **P 80** | 0.0 | 0.0 |  | 0.0 | 0.0 |
|  | **P 90** | 33.3 | 0.0 |  | 3.3 | 33.3 |
|  | **P 95** | 33.3 | 33.3 |  | 35.0 | 36.7 |
| **40-65** | **n** | 871 | 732 | 139 | 579 | 292 |
|  | **Mean (SD)** | 5.2 (14.6) | 5.1 (14.0) | 5.8 (17.5) | 5.1 (14.6) | 5.4 (14.5) |
|  | **95%CI** | [ 4.2 - 6.1 ] | [ 4.0 - 6.1 ] | [ 2.9 - 8.7 ] | [ 3.9 - 6.3 ] | [ 3.7 - 7.0 ] |
|  | **P 5** | 0.0 | 0.0 | 0.0 | 0.0 | 0.0 |
|  | **P 10** | 0.0 | 0.0 | 0.0 | 0.0 | 0.0 |
|  | **P 20** | 0.0 | 0.0 | 0.0 | 0.0 | 0.0 |
|  | **P 25** | 0.0 | 0.0 | 0.0 | 0.0 | 0.0 |
|  | **P 30** | 0.0 | 0.0 | 0.0 | 0.0 | 0.0 |
|  | **P 40** | 0.0 | 0.0 | 0.0 | 0.0 | 0.0 |
|  | **Median** | 0.0 | 0.0 | 0.0 | 0.0 | 0.0 |
|  | **P 60** | 0.0 | 0.0 | 0.0 | 0.0 | 0.0 |
|  | **P 70** | 0.0 | 0.0 | 0.0 | 0.0 | 0.0 |
|  | **P 75** | 0.0 | 0.0 | 0.0 | 0.0 | 0.0 |
|  | **P 80** | 0.0 | 0.0 | 0.0 | 0.0 | 0.0 |
|  | **P 90** | 33.3 | 33.3 | 33.3 | 33.3 | 33.3 |
|  | **P 95** | 33.3 | 33.3 | 33.3 | 33.3 | 33.3 |
| **>65** | **n** | 315 | 204 | 111 | 173 | 142 |
|  | **Mean (SD)** | 4.4 (14.6) | 3.4 (12.6) | 6.3 (17.7) | 4.0 (13.6) | 4.9 (15.9) |
|  | **95%CI** | [ 2.8 - 6.1 ] | [ 1.7 - 5.2 ] | [ 3.0 - 9.6 ] | [ 2.0 - 6.1 ] | [ 2.3 - 7.5 ] |
|  | **P 5** | 0.0 | 0.0 | 0.0 | 0.0 | 0.0 |
|  | **P 10** | 0.0 | 0.0 | 0.0 | 0.0 | 0.0 |
|  | **P 20** | 0.0 | 0.0 | 0.0 | 0.0 | 0.0 |
|  | **P 25** | 0.0 | 0.0 | 0.0 | 0.0 | 0.0 |
|  | **P 30** | 0.0 | 0.0 | 0.0 | 0.0 | 0.0 |
|  | **P 40** | 0.0 | 0.0 | 0.0 | 0.0 | 0.0 |
|  | **Median** | 0.0 | 0.0 | 0.0 | 0.0 | 0.0 |
|  | **P 60** | 0.0 | 0.0 | 0.0 | 0.0 | 0.0 |
|  | **P 70** | 0.0 | 0.0 | 0.0 | 0.0 | 0.0 |
|  | **P 75** | 0.0 | 0.0 | 0.0 | 0.0 | 0.0 |
|  | **P 80** | 0.0 | 0.0 | 0.0 | 0.0 | 0.0 |
|  | **P 90** | 33.3 | 0.0 | 33.3 | 20.0 | 33.3 |
|  | **P 95** | 33.3 | 33.3 | 33.3 | 33.3 | 33.3 |

* Deciles were not calculated for subgroups lower than 20 participants.

**Abbreviations**. **SD**: Standard Deviation; **95% CI**: 95% Confidence Interval; **P**: Percentile.

# Supplementary table 1.2.16. Reference norms at diagnosis for the Financial difficulties of EORTC QLQ-C30.

|  |  | **EORTC QLQ-C30: Financial difficulties** | | | | |
| --- | --- | --- | --- | --- | --- | --- |
|  |  |  | **Charlson** | | **TNM stage** | |
|  |  | **All** | **0** | **≥ 1** | **0 – I** | **II - III** |
| **Age (years)**  **<40** | **n** | 76 | 72 | 4* | 38 | 38 |
|  | **Mean (SD)** | 10.1 (24.4) | 10.6 (24.9) | 0.0 (0.0) | 8.8 (22.8) | 11.4 (26.0) |
|  | **95%CI** | [ 4.6 - 15.6 ] | [ 4.9 - 16.4 ] | [ 0.0 - 0.0 ] | [ 1.5 - 16.0 ] | [ 3.1 - 19.7 ] |
|  | **P 5** | 0.0 | 0.0 |  | 0.0 | 0.0 |
|  | **P 10** | 0.0 | 0.0 |  | 0.0 | 0.0 |
|  | **P 20** | 0.0 | 0.0 |  | 0.0 | 0.0 |
|  | **P 25** | 0.0 | 0.0 |  | 0.0 | 0.0 |
|  | **P 30** | 0.0 | 0.0 |  | 0.0 | 0.0 |
|  | **P 40** | 0.0 | 0.0 |  | 0.0 | 0.0 |
|  | **Median** | 0.0 | 0.0 | 0.0 | 0.0 | 0.0 |
|  | **P 60** | 0.0 | 0.0 |  | 0.0 | 0.0 |
|  | **P 70** | 0.0 | 0.0 |  | 0.0 | 0.0 |
|  | **P 75** | 0.0 | 0.0 |  | 0.0 | 0.0 |
|  | **P 80** | 0.0 | 13.3 |  | 0.0 | 33.3 |
|  | **P 90** | 33.3 | 33.3 |  | 36.7 | 36.7 |
|  | **P 95** | 71.7 | 78.3 |  | 68.3 | 100.0 |
| **40-65** | **n** | 870 | 731 | 139 | 580 | 290 |
|  | **Mean (SD)** | 6.4 (18.1) | 5.8 (16.8) | 9.8 (23.6) | 6.6 (18.4) | 6.2 (17.5) |
|  | **95%CI** | [ 5.2 - 7.6 ] | [ 4.6 - 7.0 ] | [ 5.9 - 13.7 ] | [ 5.1 - 8.0 ] | [ 4.2 - 8.2 ] |
|  | **P 5** | 0.0 | 0.0 | 0.0 | 0.0 | 0.0 |
|  | **P 10** | 0.0 | 0.0 | 0.0 | 0.0 | 0.0 |
|  | **P 20** | 0.0 | 0.0 | 0.0 | 0.0 | 0.0 |
|  | **P 25** | 0.0 | 0.0 | 0.0 | 0.0 | 0.0 |
|  | **P 30** | 0.0 | 0.0 | 0.0 | 0.0 | 0.0 |
|  | **P 40** | 0.0 | 0.0 | 0.0 | 0.0 | 0.0 |
|  | **Median** | 0.0 | 0.0 | 0.0 | 0.0 | 0.0 |
|  | **P 60** | 0.0 | 0.0 | 0.0 | 0.0 | 0.0 |
|  | **P 70** | 0.0 | 0.0 | 0.0 | 0.0 | 0.0 |
|  | **P 75** | 0.0 | 0.0 | 0.0 | 0.0 | 0.0 |
|  | **P 80** | 0.0 | 0.0 | 0.0 | 0.0 | 0.0 |
|  | **P 90** | 33.3 | 33.3 | 66.7 | 33.3 | 33.3 |
|  | **P 95** | 33.3 | 33.3 | 66.7 | 33.3 | 33.3 |
| **>65** | **n** | 318 | 203 | 115 | 176 | 142 |
|  | **Mean (SD)** | 4.5 (16.0) | 4.3 (15.7) | 4.9 (16.7) | 5.5 (17.5) | 3.3 (13.9) |
|  | **95%CI** | [ 2.7 - 6.3 ] | [ 2.1 - 6.4 ] | [ 1.9 - 8.0 ] | [ 2.9 - 8.1 ] | [ 1.0 - 5.6 ] |
|  | **P 5** | 0.0 | 0.0 | 0.0 | 0.0 | 0.0 |
|  | **P 10** | 0.0 | 0.0 | 0.0 | 0.0 | 0.0 |
|  | **P 20** | 0.0 | 0.0 | 0.0 | 0.0 | 0.0 |
|  | **P 25** | 0.0 | 0.0 | 0.0 | 0.0 | 0.0 |
|  | **P 30** | 0.0 | 0.0 | 0.0 | 0.0 | 0.0 |
|  | **P 40** | 0.0 | 0.0 | 0.0 | 0.0 | 0.0 |
|  | **Median** | 0.0 | 0.0 | 0.0 | 0.0 | 0.0 |
|  | **P 60** | 0.0 | 0.0 | 0.0 | 0.0 | 0.0 |
|  | **P 70** | 0.0 | 0.0 | 0.0 | 0.0 | 0.0 |
|  | **P 75** | 0.0 | 0.0 | 0.0 | 0.0 | 0.0 |
|  | **P 80** | 0.0 | 0.0 | 0.0 | 0.0 | 0.0 |
|  | **P 90** | 0.0 | 0.0 | 33.3 | 33.3 | 0.0 |
|  | **P 95** | 33.3 | 33.3 | 33.3 | 33.3 | 33.3 |

* Deciles were not calculated for subgroups lower than 20 participants.

**Abbreviations**. **SD**: Standard Deviation; **95% CI**: 95% Confidence Interval; **P**: Percentile.

# Supplementary table 1.3.1. Reference norms at diagnosis for the Body Image Scale of EORTC QLQ-BR23.

|  |  | **EORTC QLQ-BR23: Body Image Scale** | | | | |
| --- | --- | --- | --- | --- | --- | --- |
|  |  |  | **Charlson** | | **TNM stage** | |
|  |  | **All** | **0** | **≥ 1** | **0 – I** | **II - III** |
| **Age (years)**  **<40** | **n** | 76 | 72 | 4* | 38 | 38 |
|  | **Mean (SD)** | 87.5 (20.8) | 88.7 (19.0) | 66.7 (40.8) | 90.6 (17.7) | 84.4 (23.4) |
|  | **95%CI** | [ 82.8 - 92.2 ] | [ 84.3 - 93.1 ] | [ 26.7 - 106.7 ] | [ 85.0 - 96.2 ] | [ 77.0 - 91.9 ] |
|  | **P 5** | 32.1 | 33.3 |  | 32.9 | 16.7 |
|  | **P 10** | 50.0 | 66.7 |  | 66.7 | 48.3 |
|  | **P 20** | 83.3 | 83.3 |  | 83.3 | 75.0 |
|  | **P 25** | 83.3 | 83.3 |  | 83.3 | 83.3 |
|  | **P 30** | 83.3 | 83.3 |  | 91.7 | 83.3 |
|  | **P 40** | 91.7 | 91.7 |  | 100.0 | 83.3 |
|  | **Median** | 100.0 | 100.0 | 75.0 | 100.0 | 95.8 |
|  | **P 60** | 100.0 | 100.0 |  | 100.0 | 100.0 |
|  | **P 70** | 100.0 | 100.0 |  | 100.0 | 100.0 |
|  | **P 75** | 100.0 | 100.0 |  | 100.0 | 100.0 |
|  | **P 80** | 100.0 | 100.0 |  | 100.0 | 100.0 |
|  | **P 90** | 100.0 | 100.0 |  | 100.0 | 100.0 |
|  | **P 95** | 100.0 | 100.0 |  | 100.0 | 100.0 |
| **40-65** | **n** | 864 | 728 | 136 | 574 | 290 |
|  | **Mean (SD)** | 92.0 (17.0) | 92.2 (16.5) | 90.8 (19.5) | 92.2 (17.0) | 91.5 (17.1) |
|  | **95%CI** | [ 90.9 - 93.1 ] | [ 91.0 - 93.4 ] | [ 87.5 - 94.1 ] | [ 90.9 - 93.6 ] | [ 89.5 - 93.5 ] |
|  | **P 5** | 50.0 | 50.0 | 41.7 | 50.0 | 50.0 |
|  | **P 10** | 66.7 | 66.7 | 64.2 | 75.0 | 66.7 |
|  | **P 20** | 91.7 | 91.7 | 83.3 | 91.7 | 83.3 |
|  | **P 25** | 91.7 | 91.7 | 91.7 | 91.7 | 91.7 |
|  | **P 30** | 91.7 | 91.7 | 91.7 | 91.7 | 91.7 |
|  | **P 40** | 100.0 | 100.0 | 100.0 | 100.0 | 100.0 |
|  | **Median** | 100.0 | 100.0 | 100.0 | 100.0 | 100.0 |
|  | **P 60** | 100.0 | 100.0 | 100.0 | 100.0 | 100.0 |
|  | **P 70** | 100.0 | 100.0 | 100.0 | 100.0 | 100.0 |
|  | **P 75** | 100.0 | 100.0 | 100.0 | 100.0 | 100.0 |
|  | **P 80** | 100.0 | 100.0 | 100.0 | 100.0 | 100.0 |
|  | **P 90** | 100.0 | 100.0 | 100.0 | 100.0 | 100.0 |
|  | **P 95** | 100.0 | 100.0 | 100.0 | 100.0 | 100.0 |
| **>65** | **n** | 315 | 202 | 113 | 175 | 140 |
|  | **Mean (SD)** | 94.5 (14.4) | 94.5 (14.1) | 94.5 (14.9) | 95.3 (12.2) | 93.5 (16.7) |
|  | **95%CI** | [ 92.9 - 96.1 ] | [ 92.5 - 96.4 ] | [ 91.8 - 97.3 ] | [ 93.5 - 97.1 ] | [ 90.7 - 96.2 ] |
|  | **P 5** | 66.7 | 66.7 | 64.2 | 66.7 | 58.3 |
|  | **P 10** | 83.3 | 83.3 | 78.3 | 83.3 | 67.5 |
|  | **P 20** | 91.7 | 91.7 | 91.7 | 91.7 | 91.7 |
|  | **P 25** | 100.0 | 100.0 | 100.0 | 100.0 | 100.0 |
|  | **P 30** | 100.0 | 100.0 | 100.0 | 100.0 | 100.0 |
|  | **P 40** | 100.0 | 100.0 | 100.0 | 100.0 | 100.0 |
|  | **Median** | 100.0 | 100.0 | 100.0 | 100.0 | 100.0 |
|  | **P 60** | 100.0 | 100.0 | 100.0 | 100.0 | 100.0 |
|  | **P 70** | 100.0 | 100.0 | 100.0 | 100.0 | 100.0 |
|  | **P 75** | 100.0 | 100.0 | 100.0 | 100.0 | 100.0 |
|  | **P 80** | 100.0 | 100.0 | 100.0 | 100.0 | 100.0 |
|  | **P 90** | 100.0 | 100.0 | 100.0 | 100.0 | 100.0 |
|  | **P 95** | 100.0 | 100.0 | 100.0 | 100.0 | 100.0 |

* Deciles were not calculated for subgroups lower than 20 participants.

**Abbreviations**. **SD**: Standard Deviation; **95% CI**: 95% Confidence Interval; **P**: Percentile.

# Supplementary table 1.3.2. Reference norms at diagnosis for the Sexual Function Scale of EORTC QLQ-BR23.

|  |  | **EORTC QLQ-BR23: Sexual Function Scale** | | | | |
| --- | --- | --- | --- | --- | --- | --- |
|  |  |  | **Charlson** | | **TNM stage** | |
|  |  | **All** | **0** | **≥ 1** | **0 – I** | **II - III** |
| **Age (years)**  **<40** | **n** | 73 | 69 | 4* | 36 | 37 |
|  | **Mean (SD)** | 31.3 (27.1) | 32.1 (27.3) | 16.7 (19.2) | 32.4 (24.2) | 30.2 (29.9) |
|  | **95%CI** | [ 25.1 - 37.5 ] | [ 25.7 - 38.6 ] | [ -2.2 - 35.5 ] | [ 24.5 - 40.3 ] | [ 20.6 - 39.8 ] |
|  | **P 5** | 0.0 | 0.0 |  | 0.0 | 0.0 |
|  | **P 10** | 0.0 | 0.0 |  | 0.0 | 0.0 |
|  | **P 20** | 0.0 | 0.0 |  | 0.0 | 0.0 |
|  | **P 25** | 0.0 | 0.0 |  | 0.0 | 0.0 |
|  | **P 30** | 3.3 | 16.7 |  | 18.3 | 0.0 |
|  | **P 40** | 26.7 | 33.3 |  | 33.3 | 16.7 |
|  | **Median** | 33.3 | 33.3 | 16.7 | 33.3 | 33.3 |
|  | **P 60** | 33.3 | 33.3 |  | 33.3 | 33.3 |
|  | **P 70** | 46.7 | 50.0 |  | 50.0 | 33.3 |
|  | **P 75** | 50.0 | 50.0 |  | 50.0 | 50.0 |
|  | **P 80** | 66.7 | 66.7 |  | 60.0 | 66.7 |
|  | **P 90** | 66.7 | 66.7 |  | 66.7 | 70.0 |
|  | **P 95** | 71.7 | 75.0 |  | 66.7 | 100.0 |
| **40-65** | **n** | 846 | 713 | 133 | 558 | 288 |
|  | **Mean (SD)** | 27.4 (26.6) | 27.5 (26.0) | 26.6 (30.0) | 28.0 (26.6) | 26.2 (26.7) |
|  | **95%CI** | [ 25.6 - 29.2 ] | [ 25.6 - 29.4 ] | [ 21.5 - 31.7 ] | [ 25.8 - 30.2 ] | [ 23.1 - 29.2 ] |
|  | **P 5** | 0.0 | 0.0 | 0.0 | 0.0 | 0.0 |
|  | **P 10** | 0.0 | 0.0 | 0.0 | 0.0 | 0.0 |
|  | **P 20** | 0.0 | 0.0 | 0.0 | 0.0 | 0.0 |
|  | **P 25** | 0.0 | 0.0 | 0.0 | 0.0 | 0.0 |
|  | **P 30** | 0.0 | 0.0 | 0.0 | 0.0 | 0.0 |
|  | **P 40** | 16.7 | 16.7 | 0.0 | 16.7 | 10.0 |
|  | **Median** | 33.3 | 33.3 | 16.7 | 33.3 | 33.3 |
|  | **P 60** | 33.3 | 33.3 | 33.3 | 33.3 | 33.3 |
|  | **P 70** | 33.3 | 33.3 | 33.3 | 33.3 | 33.3 |
|  | **P 75** | 50.0 | 50.0 | 50.0 | 50.0 | 33.3 |
|  | **P 80** | 50.0 | 50.0 | 53.3 | 50.0 | 50.0 |
|  | **P 90** | 66.7 | 66.7 | 66.7 | 66.7 | 66.7 |
|  | **P 95** | 66.7 | 66.7 | 100.0 | 66.7 | 66.7 |
| **>65** | **n** | 297 | 189 | 108 | 165 | 132 |
|  | **Mean (SD)** | 10.6 (19.2) | 12.2 (20.3) | 7.9 (16.8) | 12.7 (20.7) | 8.0 (16.9) |
|  | **95%CI** | [ 8.4 - 12.8 ] | [ 9.3 - 15.1 ] | [ 4.7 - 11.0 ] | [ 9.6 - 15.9 ] | [ 5.1 - 10.8 ] |
|  | **P 5** | 0.0 | 0.0 | 0.0 | 0.0 | 0.0 |
|  | **P 10** | 0.0 | 0.0 | 0.0 | 0.0 | 0.0 |
|  | **P 20** | 0.0 | 0.0 | 0.0 | 0.0 | 0.0 |
|  | **P 25** | 0.0 | 0.0 | 0.0 | 0.0 | 0.0 |
|  | **P 30** | 0.0 | 0.0 | 0.0 | 0.0 | 0.0 |
|  | **P 40** | 0.0 | 0.0 | 0.0 | 0.0 | 0.0 |
|  | **Median** | 0.0 | 0.0 | 0.0 | 0.0 | 0.0 |
|  | **P 60** | 0.0 | 0.0 | 0.0 | 0.0 | 0.0 |
|  | **P 70** | 0.0 | 16.7 | 0.0 | 16.7 | 0.0 |
|  | **P 75** | 16.7 | 16.7 | 0.0 | 33.3 | 0.0 |
|  | **P 80** | 33.3 | 33.3 | 16.7 | 33.3 | 16.7 |
|  | **P 90** | 33.3 | 50.0 | 33.3 | 50.0 | 33.3 |
|  | **P 95** | 50.0 | 58.3 | 33.3 | 61.7 | 39.2 |

* Deciles were not calculated for subgroups lower than 20 participants.

**Abbreviations**. **SD**: Standard Deviation; **95% CI**: 95% Confidence Interval; **P**: Percentile.

# Supplementary table 1.3.3. Reference norms at diagnosis for the Sexual enjoyment Scale of EORTC QLQ-BR23.

|  |  | **EORTC QLQ-BR23: Sexual enjoyment Scale** | | | | |
| --- | --- | --- | --- | --- | --- | --- |
|  |  |  | **Charlson** | | **TNM stage** | |
|  |  | **All** | **0** | **≥ 1** | **0 – I** | **II - III** |
| **Age (years)**  **<40** | **n** | 46 | 44 | 2* | 25 | 21 |
|  | **Mean (SD)** | 59.4 (32.1) | 60.6 (32.4) | 33.3 (0.0) | 64.0 (31.8) | 54.0 (32.4) |
|  | **95%CI** | [ 50.1 - 68.7 ] | [ 51.0 - 70.2 ] | [ 33.3 - 33.3 ] | [ 51.5 - 76.5 ] | [ 40.1 - 67.8 ] |
|  | **P 5** | 0.0 | 0.0 |  | 0.0 | 0.0 |
|  | **P 10** | 0.0 | 0.0 |  | 20.0 | 0.0 |
|  | **P 20** | 33.3 | 33.3 |  | 33.3 | 33.3 |
|  | **P 25** | 33.3 | 33.3 |  | 33.3 | 33.3 |
|  | **P 30** | 33.3 | 33.3 |  | 33.3 | 33.3 |
|  | **P 40** | 66.7 | 66.7 |  | 66.7 | 33.3 |
|  | **Median** | 66.7 | 66.7 | 33.3 | 66.7 | 66.7 |
|  | **P 60** | 66.7 | 66.7 |  | 66.7 | 66.7 |
|  | **P 70** | 66.7 | 66.7 |  | 100.0 | 66.7 |
|  | **P 75** | 100.0 | 100.0 |  | 100.0 | 66.7 |
|  | **P 80** | 100.0 | 100.0 |  | 100.0 | 86.7 |
|  | **P 90** | 100.0 | 100.0 |  | 100.0 | 100.0 |
|  | **P 95** | 100.0 | 100.0 |  | 100.0 | 100.0 |
| **40-65** | **n** | 502 | 432 | 70 | 337 | 165 |
|  | **Mean (SD)** | 56.2 (28.8) | 56.3 (28.7) | 55.2 (29.4) | 56.2 (27.5) | 56.2 (31.4) |
|  | **95%CI** | [ 53.7 - 58.7 ] | [ 53.6 - 59.0 ] | [ 48.3 - 62.1 ] | [ 53.2 - 59.1 ] | [ 51.4 - 61.0 ] |
|  | **P 5** | 0.0 | 0.0 | 0.0 | 0.0 | 0.0 |
|  | **P 10** | 10.0 | 10.0 | 3.3 | 33.3 | 0.0 |
|  | **P 20** | 33.3 | 33.3 | 33.3 | 33.3 | 33.3 |
|  | **P 25** | 33.3 | 33.3 | 33.3 | 33.3 | 33.3 |
|  | **P 30** | 33.3 | 33.3 | 33.3 | 33.3 | 33.3 |
|  | **P 40** | 66.7 | 66.7 | 33.3 | 66.7 | 66.7 |
|  | **Median** | 66.7 | 66.7 | 66.7 | 66.7 | 66.7 |
|  | **P 60** | 66.7 | 66.7 | 66.7 | 66.7 | 66.7 |
|  | **P 70** | 66.7 | 66.7 | 66.7 | 66.7 | 66.7 |
|  | **P 75** | 66.7 | 66.7 | 66.7 | 66.7 | 66.7 |
|  | **P 80** | 66.7 | 66.7 | 66.7 | 66.7 | 100.0 |
|  | **P 90** | 100.0 | 100.0 | 100.0 | 100.0 | 100.0 |
|  | **P 95** | 100.0 | 100.0 | 100.0 | 100.0 | 100.0 |
| **>65** | **n** | 84 | 61 | 23 | 54 | 30 |
|  | **Mean (SD)** | 42.5 (26.1) | 44.3 (24.9) | 37.7 (29.0) | 41.4 (25.8) | 44.4 (26.7) |
|  | **95%CI** | [ 36.9 - 48.0 ] | [ 38.0 - 50.5 ] | [ 25.8 - 49.5 ] | [ 34.5 - 48.3 ] | [ 34.9 - 54.0 ] |
|  | **P 5** | 0.0 | 0.0 | 0.0 | 0.0 | 0.0 |
|  | **P 10** | 0.0 | 0.0 | 0.0 | 0.0 | 0.0 |
|  | **P 20** | 33.3 | 33.3 | 0.0 | 33.3 | 33.3 |
|  | **P 25** | 33.3 | 33.3 | 0.0 | 33.3 | 33.3 |
|  | **P 30** | 33.3 | 33.3 | 33.3 | 33.3 | 33.3 |
|  | **P 40** | 33.3 | 33.3 | 33.3 | 33.3 | 33.3 |
|  | **Median** | 33.3 | 33.3 | 33.3 | 33.3 | 33.3 |
|  | **P 60** | 66.7 | 66.7 | 33.3 | 66.7 | 66.7 |
|  | **P 70** | 66.7 | 66.7 | 66.7 | 66.7 | 66.7 |
|  | **P 75** | 66.7 | 66.7 | 66.7 | 66.7 | 66.7 |
|  | **P 80** | 66.7 | 66.7 | 66.7 | 66.7 | 66.7 |
|  | **P 90** | 66.7 | 66.7 | 66.7 | 66.7 | 66.7 |
|  | **P 95** | 66.7 | 66.7 | 93.3 | 66.7 | 81.7 |

* Deciles were not calculated for subgroups lower than 20 participants.

**Abbreviations**. **SD**: Standard Deviation; **95% CI**: 95% Confidence Interval; **P**: Percentile.

# Supplementary table 1.3.4. Reference norms at diagnosis for the Future perspective Scale of EORTC QLQ-BR23.

|  |  | **EORTC QLQ-BR23: Future perspective Scale** | | | | |
| --- | --- | --- | --- | --- | --- | --- |
|  |  |  | **Charlson** | | **TNM stage** | |
|  |  | **All** | **0** | **≥ 1** | **0 – I** | **II - III** |
| **Age (years)**  **<40** | **n** | 75 | 71 | 4* | 37 | 38 |
|  | **Mean (SD)** | 36.4 (32.0) | 37.6 (32.3) | 16.7 (19.2) | 32.4 (29.9) | 40.4 (33.9) |
|  | **95%CI** | [ 29.2 - 43.7 ] | [ 30.0 - 45.1 ] | [ -2.2 - 35.5 ] | [ 22.8 - 42.1 ] | [ 29.6 - 51.1 ] |
|  | **P 5** | 0.0 | 0.0 |  | 0.0 | 0.0 |
|  | **P 10** | 0.0 | 0.0 |  | 0.0 | 0.0 |
|  | **P 20** | 0.0 | 0.0 |  | 0.0 | 0.0 |
|  | **P 25** | 0.0 | 0.0 |  | 0.0 | 0.0 |
|  | **P 30** | 0.0 | 20.0 |  | 0.0 | 33.3 |
|  | **P 40** | 33.3 | 33.3 |  | 33.3 | 33.3 |
|  | **Median** | 33.3 | 33.3 | 16.7 | 33.3 | 33.3 |
|  | **P 60** | 33.3 | 33.3 |  | 33.3 | 33.3 |
|  | **P 70** | 40.0 | 66.7 |  | 33.3 | 66.7 |
|  | **P 75** | 66.7 | 66.7 |  | 66.7 | 66.7 |
|  | **P 80** | 66.7 | 66.7 |  | 66.7 | 66.7 |
|  | **P 90** | 100.0 | 100.0 |  | 66.7 | 100.0 |
|  | **P 95** | 100.0 | 100.0 |  | 100.0 | 100.0 |
| **40-65** | **n** | 864 | 728 | 136 | 574 | 290 |
|  | **Mean (SD)** | 45.7 (31.7) | 46.2 (31.4) | 43.1 (33.0) | 46.5 (31.7) | 44.1 (31.6) |
|  | **95%CI** | [ 43.6 - 47.8 ] | [ 43.9 - 48.4 ] | [ 37.6 - 48.7 ] | [ 43.9 - 49.1 ] | [ 40.5 - 47.8 ] |
|  | **P 5** | 0.0 | 0.0 | 0.0 | 0.0 | 0.0 |
|  | **P 10** | 0.0 | 0.0 | 0.0 | 0.0 | 0.0 |
|  | **P 20** | 33.3 | 33.3 | 0.0 | 33.3 | 6.7 |
|  | **P 25** | 33.3 | 33.3 | 33.3 | 33.3 | 33.3 |
|  | **P 30** | 33.3 | 33.3 | 33.3 | 33.3 | 33.3 |
|  | **P 40** | 33.3 | 33.3 | 33.3 | 33.3 | 33.3 |
|  | **Median** | 33.3 | 33.3 | 33.3 | 33.3 | 33.3 |
|  | **P 60** | 66.7 | 66.7 | 33.3 | 66.7 | 33.3 |
|  | **P 70** | 66.7 | 66.7 | 66.7 | 66.7 | 66.7 |
|  | **P 75** | 66.7 | 66.7 | 66.7 | 66.7 | 66.7 |
|  | **P 80** | 66.7 | 66.7 | 66.7 | 66.7 | 66.7 |
|  | **P 90** | 100.0 | 100.0 | 100.0 | 100.0 | 100.0 |
|  | **P 95** | 100.0 | 100.0 | 100.0 | 100.0 | 100.0 |
| **>65** | **n** | 316 | 202 | 114 | 174 | 142 |
|  | **Mean (SD)** | 48.8 (32.2) | 47.5 (30.4) | 51.2 (35.2) | 49.6 (31.6) | 47.9 (33.1) |
|  | **95%CI** | [ 45.3 - 52.4 ] | [ 43.3 - 51.7 ] | [ 44.7 - 57.6 ] | [ 44.9 - 54.3 ] | [ 42.4 - 53.3 ] |
|  | **P 5** | 0.0 | 0.0 | 0.0 | 0.0 | 0.0 |
|  | **P 10** | 0.0 | 0.0 | 0.0 | 0.0 | 0.0 |
|  | **P 20** | 33.3 | 33.3 | 0.0 | 33.3 | 20.0 |
|  | **P 25** | 33.3 | 33.3 | 33.3 | 33.3 | 33.3 |
|  | **P 30** | 33.3 | 33.3 | 33.3 | 33.3 | 33.3 |
|  | **P 40** | 33.3 | 33.3 | 33.3 | 33.3 | 33.3 |
|  | **Median** | 33.3 | 33.3 | 66.7 | 66.7 | 33.3 |
|  | **P 60** | 66.7 | 66.7 | 66.7 | 66.7 | 66.7 |
|  | **P 70** | 66.7 | 66.7 | 66.7 | 66.7 | 66.7 |
|  | **P 75** | 66.7 | 66.7 | 66.7 | 66.7 | 66.7 |
|  | **P 80** | 66.7 | 66.7 | 100.0 | 66.7 | 66.7 |
|  | **P 90** | 100.0 | 100.0 | 100.0 | 100.0 | 100.0 |
|  | **P 95** | 100.0 | 100.0 | 100.0 | 100.0 | 100.0 |

* Deciles were not calculated for subgroups lower than 20 participants.

**Abbreviations**. **SD**: Standard Deviation; **95% CI**: 95% Confidence Interval; **P**: Percentile.

# Supplementary table 1.3.5. Reference norms at diagnosis for the Systemic Therapy Side Effects Scale of EORTC QLQ-BR23.

|  |  | **EORTC QLQ-BR23: Systemic Therapy Side Effects** | | | | |
| --- | --- | --- | --- | --- | --- | --- |
|  |  |  | **Charlson** | | **TNM stage** | |
|  |  | **All** | **0** | **≥ 1** | **0 – I** | **II - III** |
| **Age (years)**  **<40** | **n** | 76 | 72 | 4* | 38 | 38 |
|  | **Mean (SD)** | 11.5 (13.9) | 11.4 (13.6) | 14.3 (22.3) | 10.0 (12.2) | 13.0 (15.5) |
|  | **95%CI** | [ 8.4 - 14.6 ] | [ 8.2 - 14.5 ] | [ -7.6 - 36.2 ] | [ 6.1 - 13.9 ] | [ 8.1 - 18.0 ] |
|  | **P 5** | 0.0 | 0.0 |  | 0.0 | 0.0 |
|  | **P 10** | 0.0 | 0.0 |  | 0.0 | 0.0 |
|  | **P 20** | 0.0 | 0.0 |  | 0.0 | 0.0 |
|  | **P 25** | 0.0 | 0.0 |  | 0.0 | 0.0 |
|  | **P 30** | 0.0 | 0.0 |  | 0.0 | 0.0 |
|  | **P 40** | 4.8 | 4.8 |  | 4.8 | 4.8 |
|  | **Median** | 7.1 | 9.5 | 4.8 | 7.1 | 7.1 |
|  | **P 60** | 9.5 | 9.5 |  | 9.5 | 9.5 |
|  | **P 70** | 14.3 | 14.3 |  | 14.3 | 15.7 |
|  | **P 75** | 14.3 | 14.3 |  | 14.3 | 19.0 |
|  | **P 80** | 19.0 | 19.0 |  | 15.2 | 24.8 |
|  | **P 90** | 33.3 | 31.9 |  | 23.8 | 42.9 |
|  | **P 95** | 43.6 | 42.9 |  | 34.8 | 47.9 |
| **40-65** | **n** | 868 | 731 | 137 | 576 | 292 |
|  | **Mean (SD)** | 12.7 (14.4) | 12.5 (14.3) | 13.8 (14.4) | 12.0 (13.0) | 14.0 (16.6) |
|  | **95%CI** | [ 11.7 - 13.6 ] | [ 11.4 - 13.5 ] | [ 11.4 - 16.2 ] | [ 10.9 - 13.0 ] | [ 12.1 - 15.9 ] |
|  | **P 5** | 0.0 | 0.0 | 0.0 | 0.0 | 0.0 |
|  | **P 10** | 0.0 | 0.0 | 0.0 | 0.0 | 0.0 |
|  | **P 20** | 0.0 | 0.0 | 0.0 | 0.0 | 0.0 |
|  | **P 25** | 0.0 | 0.0 | 4.8 | 1.2 | 0.0 |
|  | **P 30** | 4.8 | 4.8 | 4.8 | 4.8 | 4.8 |
|  | **P 40** | 4.8 | 4.8 | 4.9 | 4.8 | 4.8 |
|  | **Median** | 9.5 | 9.5 | 9.5 | 9.5 | 9.5 |
|  | **P 60** | 9.5 | 9.5 | 14.3 | 9.5 | 14.3 |
|  | **P 70** | 14.3 | 14.3 | 19.0 | 14.3 | 14.3 |
|  | **P 75** | 19.0 | 19.0 | 19.0 | 19.0 | 19.0 |
|  | **P 80** | 19.0 | 19.0 | 23.8 | 19.0 | 23.8 |
|  | **P 90** | 28.6 | 28.6 | 33.3 | 28.6 | 33.3 |
|  | **P 95** | 38.1 | 38.1 | 43.3 | 38.1 | 44.5 |
| **>65** | **n** | 318 | 203 | 115 | 176 | 142 |
|  | **Mean (SD)** | 12.4 (12.7) | 11.8 (11.9) | 13.3 (14.0) | 12.3 (14.0) | 12.4 (11.0) |
|  | **95%CI** | [ 11.0 - 13.8 ] | [ 10.2 - 13.4 ] | [ 10.8 - 15.9 ] | [ 10.3 - 14.4 ] | [ 10.6 - 14.2 ] |
|  | **P 5** | 0.0 | 0.0 | 0.0 | 0.0 | 0.0 |
|  | **P 10** | 0.0 | 0.0 | 0.0 | 0.0 | 0.0 |
|  | **P 20** | 0.0 | 0.0 | 0.0 | 0.0 | 4.8 |
|  | **P 25** | 4.8 | 4.8 | 4.8 | 0.0 | 4.8 |
|  | **P 30** | 4.8 | 4.8 | 4.8 | 4.8 | 4.8 |
|  | **P 40** | 4.8 | 4.8 | 4.8 | 4.8 | 9.5 |
|  | **Median** | 9.5 | 9.5 | 9.5 | 9.5 | 9.5 |
|  | **P 60** | 14.3 | 14.3 | 14.3 | 9.5 | 14.3 |
|  | **P 70** | 14.3 | 14.3 | 19.0 | 14.3 | 14.3 |
|  | **P 75** | 19.0 | 19.0 | 19.0 | 19.0 | 19.0 |
|  | **P 80** | 19.0 | 19.0 | 23.8 | 21.9 | 19.0 |
|  | **P 90** | 28.6 | 28.6 | 33.3 | 33.3 | 28.6 |
|  | **P 95** | 38.1 | 33.3 | 44.3 | 42.9 | 33.3 |

* Deciles were not calculated for subgroups lower than 20 participants.

**Abbreviations**. **SD**: Standard Deviation; **95% CI**: 95% Confidence Interval; **P**: Percentile.

# Supplementary table 1.3.6. Reference norms at diagnosis for the Breast Symptoms Scale of EORTC QLQ-BR23.

|  |  | **EORTC QLQ-BR23: Breast Symptoms Scale** | | | | |
| --- | --- | --- | --- | --- | --- | --- |
|  |  |  | **Charlson** | | **TNM stage** | |
|  |  | **All** | **0** | **≥ 1** | **0 – I** | **II - III** |
| **Age (years)**  **<40** | **n** | 73 | 69 | 4* | 36 | 37 |
|  | **Mean (SD)** | 19.2 (20.4) | 19.0 (20.7) | 22.9 (17.2) | 19.9 (23.1) | 18.5 (17.7) |
|  | **95%CI** | [ 14.5 - 23.9 ] | [ 14.1 - 23.8 ] | [ 6.1 - 39.8 ] | [ 12.4 - 27.4 ] | [ 12.8 - 24.2 ] |
|  | **P 5** | 0.0 | 0.0 |  | 0.0 | 0.0 |
|  | **P 10** | 0.0 | 0.0 |  | 0.0 | 0.0 |
|  | **P 20** | 0.0 | 0.0 |  | 0.0 | 0.0 |
|  | **P 25** | 0.0 | 0.0 |  | 0.0 | 0.0 |
|  | **P 30** | 0.0 | 0.0 |  | 0.0 | 3.3 |
|  | **P 40** | 8.3 | 8.3 |  | 8.3 | 16.7 |
|  | **Median** | 16.7 | 16.7 | 25.0 | 12.5 | 16.7 |
|  | **P 60** | 16.7 | 16.7 |  | 18.3 | 16.7 |
|  | **P 70** | 25.0 | 25.0 |  | 25.0 | 25.0 |
|  | **P 75** | 25.0 | 25.0 |  | 33.3 | 25.0 |
|  | **P 80** | 33.3 | 33.3 |  | 41.7 | 28.3 |
|  | **P 90** | 50.0 | 50.0 |  | 52.5 | 50.0 |
|  | **P 95** | 60.8 | 62.5 |  | 83.3 | 59.2 |
| **40-65** | **n** | 866 | 731 | 135 | 576 | 290 |
|  | **Mean (SD)** | 13.4 (16.9) | 14.1 (17.4) | 9.8 (13.7) | 13.0 (17.1) | 14.3 (16.6) |
|  | **95%CI** | [ 12.3 - 14.5 ] | [ 12.8 - 15.3 ] | [ 7.5 - 12.1 ] | [ 11.6 - 14.4 ] | [ 12.3 - 16.2 ] |
|  | **P 5** | 0.0 | 0.0 | 0.0 | 0.0 | 0.0 |
|  | **P 10** | 0.0 | 0.0 | 0.0 | 0.0 | 0.0 |
|  | **P 20** | 0.0 | 0.0 | 0.0 | 0.0 | 0.0 |
|  | **P 25** | 0.0 | 0.0 | 0.0 | 0.0 | 0.0 |
|  | **P 30** | 0.0 | 0.0 | 0.0 | 0.0 | 0.0 |
|  | **P 40** | 0.0 | 0.0 | 0.0 | 0.0 | 8.3 |
|  | **Median** | 8.3 | 8.3 | 8.3 | 8.3 | 8.3 |
|  | **P 60** | 16.7 | 16.7 | 8.3 | 8.9 | 16.7 |
|  | **P 70** | 16.7 | 16.7 | 16.7 | 16.7 | 16.7 |
|  | **P 75** | 22.9 | 25.0 | 16.7 | 16.7 | 25.0 |
|  | **P 80** | 25.0 | 25.0 | 16.7 | 25.0 | 25.0 |
|  | **P 90** | 33.3 | 33.3 | 25.0 | 33.3 | 33.3 |
|  | **P 95** | 50.0 | 50.0 | 41.7 | 50.0 | 50.0 |
| **>65** | **n** | 313 | 201 | 112 | 173 | 140 |
|  | **Mean (SD)** | 11.1 (15.8) | 10.7 (14.7) | 11.9 (17.6) | 9.8 (15.7) | 12.8 (15.8) |
|  | **95%CI** | [ 9.4 - 12.9 ] | [ 8.6 - 12.7 ] | [ 8.7 - 15.2 ] | [ 7.4 - 12.1 ] | [ 10.2 - 15.4 ] |
|  | **P 5** | 0.0 | 0.0 | 0.0 | 0.0 | 0.0 |
|  | **P 10** | 0.0 | 0.0 | 0.0 | 0.0 | 0.0 |
|  | **P 20** | 0.0 | 0.0 | 0.0 | 0.0 | 0.0 |
|  | **P 25** | 0.0 | 0.0 | 0.0 | 0.0 | 0.0 |
|  | **P 30** | 0.0 | 0.0 | 0.0 | 0.0 | 0.0 |
|  | **P 40** | 0.0 | 0.0 | 0.0 | 0.0 | 0.0 |
|  | **Median** | 8.3 | 8.3 | 8.3 | 0.0 | 8.3 |
|  | **P 60** | 8.3 | 8.3 | 8.3 | 8.3 | 8.3 |
|  | **P 70** | 16.7 | 16.7 | 16.7 | 16.7 | 16.7 |
|  | **P 75** | 16.7 | 16.7 | 16.7 | 16.7 | 20.8 |
|  | **P 80** | 22.8 | 23.9 | 20.0 | 16.7 | 25.0 |
|  | **P 90** | 33.3 | 25.0 | 33.3 | 25.0 | 33.3 |
|  | **P 95** | 41.7 | 33.3 | 58.3 | 35.8 | 49.6 |

* Deciles were not calculated for subgroups lower than 20 participants.

**Abbreviations**. **SD**: Standard Deviation; **95% CI**: 95% Confidence Interval; **P**: Percentile.

# Supplementary table 1.3.7. Reference norms at diagnosis for the Arm Symptoms Scale of EORTC QLQ-BR23.

|  |  | **EORTC QLQ-BR23: Arm Symptoms Scale** | | | | |
| --- | --- | --- | --- | --- | --- | --- |
|  |  |  | **Charlson** | | **TNM stage** | |
|  |  | **All** | **0** | **≥ 1** | **0 – I** | **II - III** |
| **Age (years)**  **<40** | **n** | 73 | 69 | 4* | 36 | 37 |
|  | **Mean (SD)** | 6.4 (12.5) | 5.5 (11.2) | 22.2 (24.0) | 6.5 (12.0) | 6.3 (13.2) |
|  | **95%CI** | [ 3.5 - 9.3 ] | [ 2.8 - 8.1 ] | [ -1.3 - 45.7 ] | [ 2.6 - 10.4 ] | [ 2.0 - 10.6 ] |
|  | **P 5** | 0.0 | 0.0 |  | 0.0 | 0.0 |
|  | **P 10** | 0.0 | 0.0 |  | 0.0 | 0.0 |
|  | **P 20** | 0.0 | 0.0 |  | 0.0 | 0.0 |
|  | **P 25** | 0.0 | 0.0 |  | 0.0 | 0.0 |
|  | **P 30** | 0.0 | 0.0 |  | 0.0 | 0.0 |
|  | **P 40** | 0.0 | 0.0 |  | 0.0 | 0.0 |
|  | **Median** | 0.0 | 0.0 | 16.7 | 0.0 | 0.0 |
|  | **P 60** | 0.0 | 0.0 |  | 0.0 | 0.0 |
|  | **P 70** | 0.0 | 0.0 |  | 0.0 | 0.0 |
|  | **P 75** | 11.1 | 5.6 |  | 11.1 | 11.1 |
|  | **P 80** | 11.1 | 11.1 |  | 17.8 | 11.1 |
|  | **P 90** | 22.2 | 22.2 |  | 25.6 | 24.4 |
|  | **P 95** | 36.7 | 33.3 |  | 35.0 | 45.6 |
| **40-65** | **n** | 866 | 731 | 135 | 576 | 290 |
|  | **Mean (SD)** | 8.4 (14.7) | 8.1 (14.4) | 10.1 (16.1) | 8.1 (14.6) | 9.2 (14.8) |
|  | **95%CI** | [ 7.5 - 9.4 ] | [ 7.1 - 9.2 ] | [ 7.4 - 12.8 ] | [ 6.9 - 9.3 ] | [ 7.5 - 10.9 ] |
|  | **P 5** | 0.0 | 0.0 | 0.0 | 0.0 | 0.0 |
|  | **P 10** | 0.0 | 0.0 | 0.0 | 0.0 | 0.0 |
|  | **P 20** | 0.0 | 0.0 | 0.0 | 0.0 | 0.0 |
|  | **P 25** | 0.0 | 0.0 | 0.0 | 0.0 | 0.0 |
|  | **P 30** | 0.0 | 0.0 | 0.0 | 0.0 | 0.0 |
|  | **P 40** | 0.0 | 0.0 | 0.0 | 0.0 | 0.0 |
|  | **Median** | 0.0 | 0.0 | 0.0 | 0.0 | 0.0 |
|  | **P 60** | 0.0 | 0.0 | 11.1 | 0.0 | 11.1 |
|  | **P 70** | 11.1 | 11.1 | 11.1 | 11.1 | 11.1 |
|  | **P 75** | 11.1 | 11.1 | 11.1 | 11.1 | 11.1 |
|  | **P 80** | 11.1 | 11.1 | 22.2 | 11.1 | 20.0 |
|  | **P 90** | 22.2 | 22.2 | 33.3 | 22.2 | 33.3 |
|  | **P 95** | 44.4 | 44.4 | 46.7 | 44.4 | 44.4 |
| **>65** | **n** | 311 | 201 | 110 | 172 | 139 |
|  | **Mean (SD)** | 10.0 (18.3) | 9.2 (16.7) | 11.6 (20.9) | 9.5 (17.8) | 10.7 (19.0) |
|  | **95%CI** | [ 8.0 - 12.1 ] | [ 6.9 - 11.5 ] | [ 7.7 - 15.5 ] | [ 6.8 - 12.2 ] | [ 7.6 - 13.9 ] |
|  | **P 5** | 0.0 | 0.0 | 0.0 | 0.0 | 0.0 |
|  | **P 10** | 0.0 | 0.0 | 0.0 | 0.0 | 0.0 |
|  | **P 20** | 0.0 | 0.0 | 0.0 | 0.0 | 0.0 |
|  | **P 25** | 0.0 | 0.0 | 0.0 | 0.0 | 0.0 |
|  | **P 30** | 0.0 | 0.0 | 0.0 | 0.0 | 0.0 |
|  | **P 40** | 0.0 | 0.0 | 0.0 | 0.0 | 0.0 |
|  | **Median** | 0.0 | 0.0 | 0.0 | 0.0 | 0.0 |
|  | **P 60** | 0.0 | 0.0 | 0.0 | 0.0 | 0.0 |
|  | **P 70** | 11.1 | 11.1 | 11.1 | 11.1 | 11.1 |
|  | **P 75** | 11.1 | 11.1 | 11.1 | 11.1 | 11.1 |
|  | **P 80** | 22.2 | 14.4 | 22.2 | 22.2 | 22.2 |
|  | **P 90** | 33.3 | 31.1 | 44.4 | 22.2 | 33.3 |
|  | **P 95** | 55.6 | 44.4 | 66.7 | 55.6 | 55.6 |

* Deciles were not calculated for subgroups lower than 20 participants.

**Abbreviations**. **SD**: Standard Deviation; **95% CI**: 95% Confidence Interval; **P**: Percentile.

# Supplementary table 1.3.8. Reference norms at diagnosis for the Upset by Hair Loss Scale of EORTC QLQ-BR23.

|  |  | **EORTC QLQ-BR23: Upset by Hair Loss Scale** | | | | |
| --- | --- | --- | --- | --- | --- | --- |
|  |  |  | **Charlson** | | **TNM stage** | |
|  |  | **All** | **0** | **≥ 1** | **0 – I** | **II - III** |
| **Age (years)**  **<40** | **n** | 19* | 19* | 0* | 7* | 12* |
|  | **Mean (SD)** | 12.3 (27.7) | 12.3 (27.7) |  |  | 19.4 (33.2) |
|  | **95%CI** | [ -0.2 - 24.7 ] | [ -0.2 - 24.7 ] |  |  | [ 0.7 - 38.2 ] |
|  | **P 5** |  |  |  |  |  |
|  | **P 10** |  |  |  |  |  |
|  | **P 20** |  |  |  |  |  |
|  | **P 25** |  |  |  |  |  |
|  | **P 30** |  |  |  |  |  |
|  | **P 40** |  |  |  |  |  |
|  | **Median** | 0.0 | 0.0 |  | 0.0 | 0.0 |
|  | **P 60** |  |  |  |  |  |
|  | **P 70** |  |  |  |  |  |
|  | **P 75** |  |  |  |  |  |
|  | **P 80** |  |  |  |  |  |
|  | **P 90** |  |  |  |  |  |
|  | **P 95** |  |  |  |  |  |
| **40-65** | **n** | 159 | 141 | 18* | 105 | 54 |
|  | **Mean (SD)** | 25.6 (33.2) | 25.1 (32.6) | 29.6 (37.7) | 21.3 (32.1) | 34.0 (34.0) |
|  | **95%CI** | [ 20.4 - 30.7 ] | [ 19.7 - 30.4 ] | [ 12.2 - 47.1 ] | [ 15.1 - 27.4 ] | [ 24.9 - 43.0 ] |
|  | **P 5** | 0.0 | 0.0 |  | 0.0 | 0.0 |
|  | **P 10** | 0.0 | 0.0 |  | 0.0 | 0.0 |
|  | **P 20** | 0.0 | 0.0 |  | 0.0 | 0.0 |
|  | **P 25** | 0.0 | 0.0 |  | 0.0 | 0.0 |
|  | **P 30** | 0.0 | 0.0 |  | 0.0 | 0.0 |
|  | **P 40** | 0.0 | 0.0 |  | 0.0 | 33.3 |
|  | **Median** | 0.0 | 0.0 | 0.0 | 0.0 | 33.3 |
|  | **P 60** | 33.3 | 33.3 |  | 0.0 | 33.3 |
|  | **P 70** | 33.3 | 33.3 |  | 33.3 | 50.0 |
|  | **P 75** | 33.3 | 33.3 |  | 33.3 | 66.7 |
|  | **P 80** | 66.7 | 66.7 |  | 66.7 | 66.7 |
|  | **P 90** | 66.7 | 66.7 |  | 66.7 | 100.0 |
|  | **P 95** | 100.0 | 100.0 |  | 100.0 | 100.0 |
| **>65** | **n** | 55 | 33 | 22 | 33 | 22 |
|  | **Mean (SD)** | 23.6 (27.7) | 23.2 (27.0) | 24.2 (29.4) | 23.2 (29.4) | 24.2 (25.6) |
|  | **95%CI** | [ 16.3 - 31.0 ] | [ 14.0 - 32.4 ] | [ 11.9 - 36.5 ] | [ 13.2 - 33.3 ] | [ 13.6 - 34.9 ] |
|  | **P 5** | 0.0 | 0.0 | 0.0 | 0.0 | 0.0 |
|  | **P 10** | 0.0 | 0.0 | 0.0 | 0.0 | 0.0 |
|  | **P 20** | 0.0 | 0.0 | 0.0 | 0.0 | 0.0 |
|  | **P 25** | 0.0 | 0.0 | 0.0 | 0.0 | 0.0 |
|  | **P 30** | 0.0 | 0.0 | 0.0 | 0.0 | 0.0 |
|  | **P 40** | 0.0 | 0.0 | 0.0 | 0.0 | 0.0 |
|  | **Median** | 33.3 | 33.3 | 16.7 | 0.0 | 33.3 |
|  | **P 60** | 33.3 | 33.3 | 33.3 | 33.3 | 33.3 |
|  | **P 70** | 33.3 | 33.3 | 33.3 | 33.3 | 33.3 |
|  | **P 75** | 33.3 | 33.3 | 33.3 | 33.3 | 33.3 |
|  | **P 80** | 33.3 | 33.3 | 46.7 | 33.3 | 46.7 |
|  | **P 90** | 66.7 | 66.7 | 66.7 | 66.7 | 66.7 |
|  | **P 95** | 73.3 | 76.7 | 95.0 | 100.0 | 66.7 |

* Deciles were not calculated for subgroups lower than 20 participants.

**Abbreviations**. **SD**: Standard Deviation; **95% CI**: 95% Confidence Interval; **P**: Percentile.

# REFERENCE NORMS AT 2 YEARS

# Supplementary table 2.1.1. Reference norms at 2-year follow-up for the EQ-5D-5L Index.

|  |  | **EQ-5D-5L Index** | | | | |
| --- | --- | --- | --- | --- | --- | --- |
|  |  |  | **Charlson** | | **TNM stage** | |
|  |  | **All** | **0** | **≥ 1** | **0 – I** | **II - III** |
| **Age (years)**  **<40** | **n** | 35 | 33 | 2* | 14* | 21 |
|  | **Mean (SD)** | 0.896 (0.125) | 0.898 (0.127) | 0.860 (0.088) | 0.922 (0.072) | 0.879 (0.149) |
|  | **95%CI** | [ 0.855 - 0.937 ] | [ 0.855 - 0.942 ] | [ 0.737 - 0.982 ] | [ 0.884 - 0.960 ] | [ 0.815 - 0.943 ] |
|  | **P 5** | 0.672 | 0.633 |  |  | 0.397 |
|  | **P 10** | 0.762 | 0.758 |  |  | 0.754 |
|  | **P 20** | 0.805 | 0.830 |  |  | 0.795 |
|  | **P 25** | 0.841 | 0.841 |  |  | 0.797 |
|  | **P 30** | 0.841 | 0.848 |  |  | 0.822 |
|  | **P 40** | 0.919 | 0.919 |  |  | 0.866 |
|  | **Median** | 0.919 | 0.919 | 0.860 | 0.919 | 0.922 |
|  | **P 60** | 0.922 | 0.936 |  |  | 0.938 |
|  | **P 70** | 1.000 | 1.000 |  |  | 1.000 |
|  | **P 75** | 1.000 | 1.000 |  |  | 1.000 |
|  | **P 80** | 1.000 | 1.000 |  |  | 1.000 |
|  | **P 90** | 1.000 | 1.000 |  |  | 1.000 |
|  | **P 95** | 1.000 | 1.000 |  |  | 1.000 |
| **40-65** | **n** | 730 | 618 | 112 | 485 | 245 |
|  | **Mean (SD)** | 0.857 (0.162) | 0.871 (0.146) | 0.782 (0.216) | 0.867 (0.163) | 0.837 (0.158) |
|  | **95%CI** | [ 0.845 - 0.869 ] | [ 0.859 - 0.882 ] | [ 0.742 - 0.822 ] | [ 0.853 - 0.882 ] | [ 0.817 - 0.856 ] |
|  | **P 5** | 0.530 | 0.578 | 0.314 | 0.537 | 0.501 |
|  | **P 10** | 0.657 | 0.685 | 0.477 | 0.685 | 0.632 |
|  | **P 20** | 0.756 | 0.771 | 0.639 | 0.793 | 0.714 |
|  | **P 25** | 0.794 | 0.797 | 0.714 | 0.797 | 0.750 |
|  | **P 30** | 0.818 | 0.840 | 0.757 | 0.841 | 0.794 |
|  | **P 40** | 0.841 | 0.872 | 0.797 | 0.873 | 0.841 |
|  | **Median** | 0.919 | 0.919 | 0.841 | 0.919 | 0.872 |
|  | **P 60** | 0.922 | 0.922 | 0.869 | 0.922 | 0.919 |
|  | **P 70** | 1.000 | 1.000 | 0.916 | 1.000 | 0.922 |
|  | **P 75** | 1.000 | 1.000 | 0.919 | 1.000 | 1.000 |
|  | **P 80** | 1.000 | 1.000 | 0.922 | 1.000 | 1.000 |
|  | **P 90** | 1.000 | 1.000 | 1.000 | 1.000 | 1.000 |
|  | **P 95** | 1.000 | 1.000 | 1.000 | 1.000 | 1.000 |
| **>65** | **n** | 318 | 213 | 105 | 189 | 129 |
|  | **Mean (SD)** | 0.810 (0.201) | 0.839 (0.176) | 0.753 (0.235) | 0.824 (0.183) | 0.790 (0.225) |
|  | **95%CI** | [ 0.788 - 0.833 ] | [ 0.815 - 0.863 ] | [ 0.708 - 0.798 ] | [ 0.798 - 0.850 ] | [ 0.751 - 0.829 ] |
|  | **P 5** | 0.418 | 0.474 | 0.293 | 0.426 | 0.344 |
|  | **P 10** | 0.524 | 0.598 | 0.408 | 0.584 | 0.472 |
|  | **P 20** | 0.666 | 0.725 | 0.570 | 0.698 | 0.620 |
|  | **P 25** | 0.721 | 0.769 | 0.650 | 0.750 | 0.659 |
|  | **P 30** | 0.763 | 0.794 | 0.692 | 0.774 | 0.739 |
|  | **P 40** | 0.821 | 0.841 | 0.756 | 0.823 | 0.815 |
|  | **Median** | 0.841 | 0.899 | 0.815 | 0.841 | 0.850 |
|  | **P 60** | 0.919 | 0.922 | 0.841 | 0.919 | 0.919 |
|  | **P 70** | 0.922 | 0.955 | 0.917 | 0.956 | 0.922 |
|  | **P 75** | 1.000 | 1.000 | 0.922 | 1.000 | 0.922 |
|  | **P 80** | 1.000 | 1.000 | 1.000 | 1.000 | 1.000 |
|  | **P 90** | 1.000 | 1.000 | 1.000 | 1.000 | 1.000 |
|  | **P 95** | 1.000 | 1.000 | 1.000 | 1.000 | 1.000 |

* Deciles were not calculated for subgroups lower than 20 participants.

**Abbreviations**. **SD**: Standard Deviation; **95% CI**: 95% Confidence Interval; **P**: Percentile.

# Supplementary table 2.1.2. Reference norms at 2-year follow-up for the EQ-VAS.

|  |  | **EQ-VAS** | | | | |
| --- | --- | --- | --- | --- | --- | --- |
|  |  |  | **Charlson** | | **TNM stage** | |
|  |  | **All** | **0** | **≥ 1** | **0 – I** | **II - III** |
| **Age (years)**  **<40** | **n** | 35 | 33 | 2* | 14* | 21 |
|  | **Mean (SD)** | 79.7 (13.8) | 80.0 (14.1) | 75.0 (7.1) | 84.3 (7.3) | 76.7 (16.2) |
|  | **95%CI** | [ 75.2 - 84.3 ] | [ 75.2 - 84.8 ] | [ 65.2 - 84.8 ] | [ 80.5 - 88.1 ] | [ 69.7 - 83.6 ] |
|  | **P 5** | 43.0 | 42.0 |  |  | 36.0 |
|  | **P 10** | 60.0 | 60.0 |  |  | 48.0 |
|  | **P 20** | 70.0 | 70.0 |  |  | 64.0 |
|  | **P 25** | 75.0 | 75.0 |  |  | 70.0 |
|  | **P 30** | 79.0 | 80.0 |  |  | 70.0 |
|  | **P 40** | 80.0 | 80.0 |  |  | 75.0 |
|  | **Median** | 80.0 | 80.0 | 75.0 | 82.5 | 80.0 |
|  | **P 60** | 85.0 | 85.0 |  |  | 85.0 |
|  | **P 70** | 90.0 | 90.0 |  |  | 87.0 |
|  | **P 75** | 90.0 | 90.0 |  |  | 90.0 |
|  | **P 80** | 90.0 | 90.0 |  |  | 90.0 |
|  | **P 90** | 92.0 | 93.0 |  |  | 94.0 |
|  | **P 95** | 100.0 | 100.0 |  |  | 99.5 |
| **40-65** | **n** | 732 | 620 | 112 | 490 | 242 |
|  | **Mean (SD)** | 76.2 (17.7) | 77.4 (17.0) | 69.8 (20.1) | 77.3 (17.2) | 74.2 (18.6) |
|  | **95%CI** | [ 75.0 - 77.5 ] | [ 76.1 - 78.7 ] | [ 66.1 - 73.5 ] | [ 75.7 - 78.8 ] | [ 71.8 - 76.5 ] |
|  | **P 5** | 40.0 | 45.0 | 28.3 | 42.8 | 40.0 |
|  | **P 10** | 50.0 | 50.0 | 40.0 | 50.0 | 50.0 |
|  | **P 20** | 60.0 | 65.0 | 50.0 | 65.0 | 60.0 |
|  | **P 25** | 70.0 | 70.0 | 60.0 | 70.0 | 65.0 |
|  | **P 30** | 70.0 | 70.6 | 60.0 | 70.0 | 70.0 |
|  | **P 40** | 75.0 | 80.0 | 70.0 | 75.0 | 75.0 |
|  | **Median** | 80.0 | 80.0 | 70.0 | 80.0 | 80.0 |
|  | **P 60** | 80.0 | 85.0 | 75.0 | 85.0 | 80.0 |
|  | **P 70** | 90.0 | 90.0 | 80.5 | 90.0 | 85.0 |
|  | **P 75** | 90.0 | 90.0 | 85.0 | 90.0 | 90.0 |
|  | **P 80** | 90.0 | 90.0 | 90.0 | 90.0 | 90.0 |
|  | **P 90** | 95.0 | 95.0 | 95.0 | 95.0 | 95.0 |
|  | **P 95** | 100.0 | 100.0 | 95.0 | 100.0 | 100.0 |
| **>65** | **n** | 323 | 214 | 109 | 192 | 131 |
|  | **Mean (SD)** | 70.1 (19.8) | 73.3 (18.4) | 63.9 (21.1) | 70.9 (20.6) | 69.0 (18.6) |
|  | **95%CI** | [ 68.0 - 72.3 ] | [ 70.8 - 75.7 ] | [ 60.0 - 67.9 ] | [ 68.0 - 73.8 ] | [ 65.8 - 72.2 ] |
|  | **P 5** | 31.0 | 40.0 | 22.5 | 30.0 | 33.0 |
|  | **P 10** | 42.0 | 50.0 | 35.0 | 41.5 | 41.0 |
|  | **P 20** | 50.0 | 60.0 | 50.0 | 50.0 | 50.0 |
|  | **P 25** | 55.0 | 60.0 | 50.0 | 60.0 | 50.0 |
|  | **P 30** | 60.0 | 65.0 | 50.0 | 60.0 | 60.0 |
|  | **P 40** | 70.0 | 70.0 | 60.0 | 70.0 | 70.0 |
|  | **Median** | 75.0 | 75.0 | 65.0 | 75.0 | 75.0 |
|  | **P 60** | 80.0 | 80.0 | 70.0 | 80.0 | 75.6 |
|  | **P 70** | 80.0 | 85.0 | 75.0 | 85.0 | 80.0 |
|  | **P 75** | 85.0 | 90.0 | 80.0 | 85.0 | 80.0 |
|  | **P 80** | 90.0 | 90.0 | 85.0 | 90.0 | 85.0 |
|  | **P 90** | 95.0 | 95.0 | 90.0 | 97.1 | 90.0 |
|  | **P 95** | 100.0 | 100.0 | 96.5 | 100.0 | 97.0 |

* Deciles were not calculated for subgroups lower than 20 participants.

**Abbreviations. SD:** Standard Deviation; **95% CI:** 95% Confidence Interval; **P:** Percentile.

# Supplementary table 2.1.3. Reference norms at 2-year follow-up for the dimensions of the EQ-5D-5L.

|  |  | **All** | | **Charlson** | | | | **TNM** | | | |
| --- | --- | --- | --- | --- | --- | --- | --- | --- | --- | --- | --- |
|  |  | **All** | | **0** | | **≥ 1** | | **0 – I** | | **II - III** | |
| **Age (years)** |  | **%** | **SE** | **%** | **SE** | **%** | **SE** | **%** | **SE** | **%** | **SE** |
| **Mobility** |  |  |  |  |  |  |  |  |  |  |  |
| **<40**  **(n = 76)** | No problems | 94.3% | 0.04 | 93.9% | 0.04 | * | | 100.0% | 0.00 | 90.5% | 0.05 |
|  | Slight problems | 5.7% | 0.04 | 6.1% | 0.04 |  |  | 0.0% | 0.00 | 9.5% | 0.05 |
|  | Moderate problems | 0.0% | 0.00 | 0.0% | 0.00 |  |  | 0.0% | 0.00 | 0.0% | 0.00 |
|  | Severe problems | 0.0% | 0.00 | 0.0% | 0.00 |  |  | 0.0% | 0.00 | 0.0% | 0.00 |
|  | Unable | 0.0% | 0.00 | 0.0% | 0.00 |  |  | 0.0% | 0.00 | 0.0% | 0.00 |
| **40-65**  **(n = 878)** | No problems | 78.7% | 0.02 | 82.7% | 0.01 | 56.6% | 0.02 | 80.2% | 0.01 | 75.5% | 0.02 |
|  | Slight problems | 14.0% | 0.01 | 11.9% | 0.01 | 25.7% | 0.02 | 12.4% | 0.01 | 17.1% | 0.01 |
|  | Moderate problems | 6.1% | 0.01 | 5.0% | 0.01 | 12.4% | 0.01 | 6.1% | 0.01 | 6.1% | 0.01 |
|  | Severe problems | 1.0% | 0.00 | 0.2% | 0.00 | 5.3% | 0.01 | 0.8% | 0.00 | 1.2% | 0.00 |
|  | Unable | 0.3% | 0.00 | 0.3% | 0.00 | 0.0% | 0.00 | 0.4% | 0.00 | 0.0% | 0.00 |
| **>65**  **(n = 320)** | No problems | 61.4% | 0.03 | 67.1% | 0.03 | 50.0% | 0.03 | 62.0% | 0.03 | 60.6% | 0.03 |
|  | Slight problems | 18.8% | 0.02 | 18.5% | 0.02 | 19.4% | 0.02 | 21.4% | 0.02 | 15.2% | 0.02 |
|  | Moderate problems | 13.6% | 0.02 | 10.2% | 0.02 | 20.4% | 0.02 | 12.0% | 0.02 | 15.9% | 0.02 |
|  | Severe problems | 5.9% | 0.01 | 4.2% | 0.01 | 9.3% | 0.02 | 4.2% | 0.01 | 8.3% | 0.02 |
|  | Unable | 0.3% | 0.00 | 0.0% | 0.00 | 0.9% | 0.01 | 0.5% | 0.00 | 0.0% | 0.00 |
| **Self-Care** |  |  |  |  |  |  |  |  |  |  |  |
| **<40**  **(n = 76)** | No problems | 94.3% | 0.04 | 93.9% | 0.04 |  |  | 100.0% | 0.00 | 90.5% | 0.05 |
|  | Slight problems | 2.9% | 0.03 | 3.0% | 0.03 |  |  | 0.0% | 0.00 | 4.8% | 0.04 |
|  | Moderate problems | 2.9% | 0.03 | 3.0% | 0.03 |  |  | 0.0% | 0.00 | 4.8% | 0.04 |
|  | Severe problems | 0.0% | 0.00 | 0.0% | 0.00 |  |  | 0.0% | 0.00 | 0.0% | 0.00 |
|  | Unable | 0.0% | 0.00 | 0.0% | 0.00 |  |  | 0.0% | 0.00 | 0.0% | 0.00 |
| **40-65**  **(n = 876)** | No problems | 92.5% | 0.01 | 94.4% | 0.01 | 82.3% | 0.01 | 93.9% | 0.01 | 89.8% | 0.01 |
|  | Slight problems | 4.9% | 0.01 | 3.9% | 0.01 | 10.6% | 0.01 | 3.7% | 0.01 | 7.3% | 0.01 |
|  | Moderate problems | 2.2% | 0.01 | 1.4% | 0.00 | 6.2% | 0.01 | 2.0% | 0.01 | 2.4% | 0.01 |
|  | Severe problems | 0.1% | 0.00 | 0.0% | 0.00 | 0.9% | 0.00 | 0.0% | 0.00 | 0.4% | 0.00 |
|  | Unable | 0.3% | 0.00 | 0.3% | 0.00 | 0.0% | 0.00 | 0.4% | 0.00 | 0.0% | 0.00 |
| **>65**  **(n = 320)** | No problems | 83.2% | 0.02 | 87.4% | 0.02 | 74.8% | 0.02 | 88.0% | 0.02 | 76.2% | 0.02 |
|  | Slight problems | 7.5% | 0.01 | 6.5% | 0.01 | 9.3% | 0.02 | 6.3% | 0.01 | 9.2% | 0.02 |
|  | Moderate problems | 6.2% | 0.01 | 5.6% | 0.01 | 7.5% | 0.01 | 3.6% | 0.01 | 10.0% | 0.02 |
|  | Severe problems | 2.2% | 0.01 | 0.5% | 0.00 | 5.6% | 0.01 | 0.5% | 0.00 | 4.6% | 0.01 |
|  | Unable | 0.9% | 0.01 | 0.0% | 0.00 | 2.8% | 0.01 | 1.6% | 0.01 | 0.0% | 0.00 |
| **Usual Activities** | |  |  |  |  |  |  |  |  |  |  |
| **<40**  **(n = 76)** | No problems | 80.0% | 0.07 | 81.8% | 0.07 |  |  | 85.7% | 0.06 | 76.2% | 0.07 |
|  | Slight problems | 11.4% | 0.05 | 9.1% | 0.05 |  |  | 14.3% | 0.06 | 9.5% | 0.05 |
|  | Moderate problems | 5.7% | 0.04 | 6.1% | 0.04 |  |  | 0.0% | 0.00 | 9.5% | 0.05 |
|  | Severe problems | 2.9% | 0.03 | 3.0% | 0.03 |  |  | 0.0% | 0.00 | 4.8% | 0.04 |
|  | Unable | 0.0% | 0.00 | 0.0% | 0.00 |  |  | 0.0% | 0.00 | 0.0% | 0.00 |
| **40-65**  **(n = 877)** | No problems | 71.2% | 0.02 | 73.7% | 0.02 | 57.5% | 0.02 | 74.7% | 0.02 | 64.2% | 0.02 |
|  | Slight problems | 18.6% | 0.01 | 17.7% | 0.01 | 23.9% | 0.02 | 16.9% | 0.01 | 22.0% | 0.02 |
|  | Moderate problems | 8.0% | 0.01 | 7.1% | 0.01 | 13.3% | 0.01 | 6.3% | 0.01 | 11.4% | 0.01 |
|  | Severe problems | 1.5% | 0.00 | 1.1% | 0.00 | 3.5% | 0.01 | 1.0% | 0.00 | 2.4% | 0.01 |
|  | Unable | 0.7% | 0.00 | 0.5% | 0.00 | 1.8% | 0.00 | 1.0% | 0.00 | 0.0% | 0.00 |
| **>65**  **(n = 320)** | No problems | 62.7% | 0.03 | 68.1% | 0.03 | 51.9% | 0.03 | 65.6% | 0.03 | 58.3% | 0.03 |
|  | Slight problems | 18.2% | 0.02 | 17.1% | 0.02 | 20.4% | 0.02 | 19.3% | 0.02 | 16.7% | 0.02 |
|  | Moderate problems | 13.9% | 0.02 | 12.0% | 0.02 | 17.6% | 0.02 | 11.5% | 0.02 | 17.4% | 0.02 |
|  | Severe problems | 4.3% | 0.01 | 2.3% | 0.01 | 8.3% | 0.02 | 2.6% | 0.01 | 6.8% | 0.01 |
|  | Unable | 0.9% | 0.01 | 0.5% | 0.00 | 1.9% | 0.01 | 1.0% | 0.01 | 0.8% | 0.00 |
| **Pain** |  |  |  |  |  |  |  |  |  |  |  |
| **<40**  **(n = 75)** | No pain | 57.1% | 0.08 | 60.6% | 0.08 |  |  | 71.4% | 0.08 | 47.6% | 0.08 |
|  | Slight pain | 34.3% | 0.08 | 30.3% | 0.08 |  |  | 28.6% | 0.08 | 38.1% | 0.08 |
|  | Moderate pain | 5.7% | 0.04 | 6.1% | 0.04 |  |  | 0.0% | 0.00 | 9.5% | 0.05 |
|  | Severe pain | 2.9% | 0.03 | 3.0% | 0.03 |  |  | 0.0% | 0.00 | 4.8% | 0.04 |
|  | Extreme pain | 0.0% | 0.00 | 0.0% | 0.00 |  |  | 0.0% | 0.00 | 0.0% | 0.00 |
| **40-65**  **(n = 878)** | No pain | 44.5% | 0.02 | 46.5% | 0.02 | 33.6% | 0.02 | 48.6% | 0.02 | 36.3% | 0.02 |
|  | Slight pain | 36.2% | 0.02 | 36.7% | 0.02 | 33.6% | 0.02 | 34.1% | 0.02 | 40.4% | 0.02 |
|  | Moderate pain | 14.7% | 0.01 | 13.3% | 0.01 | 22.1% | 0.02 | 13.2% | 0.01 | 17.6% | 0.01 |
|  | Severe pain | 4.5% | 0.01 | 3.5% | 0.01 | 9.7% | 0.01 | 3.9% | 0.01 | 5.7% | 0.01 |
|  | Extreme pain | 0.1% | 0.00 | 0.0% | 0.00 | 0.9% | 0.00 | 0.2% | 0.00 | 0.0% | 0.00 |
| **>65**  **(n = 320)** | No pain | 40.3% | 0.03 | 44.9% | 0.03 | 31.1% | 0.03 | 43.9% | 0.03 | 35.1% | 0.03 |
|  | Slight pain | 35.9% | 0.03 | 37.9% | 0.03 | 32.1% | 0.03 | 34.4% | 0.03 | 38.2% | 0.03 |
|  | Moderate pain | 18.1% | 0.02 | 13.6% | 0.02 | 27.4% | 0.02 | 16.9% | 0.02 | 19.8% | 0.02 |
|  | Severe pain | 5.6% | 0.01 | 3.7% | 0.01 | 9.4% | 0.02 | 4.8% | 0.01 | 6.9% | 0.01 |
|  | Extreme pain | 0.0% | 0.00 | 0.0% | 0.00 | 0.0% | 0.00 | 0.0% | 0.00 | 0.0% | 0.00 |
| **Anxiety/Depression** | |  |  |  |  |  |  |  |  |  |  |
| **<40**  **(n = 75)** | Not anxious | 54.3% | 0.08 | 54.5% | 0.08 |  |  | 42.9% | 0.08 | 61.9% | 0.08 |
|  | Slightly anxious | 28.6% | 0.08 | 27.3% | 0.08 |  |  | 50.0% | 0.08 | 14.3% | 0.06 |
|  | Moderately anxious | 17.1% | 0.06 | 18.2% | 0.07 |  |  | 7.1% | 0.04 | 23.8% | 0.07 |
|  | Severely anxious | 0.0% | 0.00 | 0.0% | 0.00 |  |  | 0.0% | 0.00 | 0.0% | 0.00 |
|  | Extremely anxious | 0.0% | 0.00 | 0.0% | 0.00 |  |  | 0.0% | 0.00 | 0.0% | 0.00 |
| **40-65**  **(n = 878)** | Not anxious | 55.8% | 0.02 | 57.2% | 0.02 | 47.8% | 0.02 | 58.3% | 0.02 | 50.8% | 0.02 |
|  | Slightly anxious | 30.5% | 0.02 | 29.7% | 0.02 | 34.5% | 0.02 | 30.5% | 0.02 | 30.5% | 0.02 |
|  | Moderately anxious | 9.8% | 0.01 | 9.8% | 0.01 | 9.7% | 0.01 | 6.7% | 0.01 | 15.9% | 0.01 |
|  | Severely anxious | 3.4% | 0.01 | 2.7% | 0.01 | 7.1% | 0.01 | 3.7% | 0.01 | 2.8% | 0.01 |
|  | Extremely anxious | 0.5% | 0.00 | 0.5% | 0.00 | 0.9% | 0.00 | 0.8% | 0.00 | 0.0% | 0.00 |
| **>65**  **(n = 319)** | Not anxious | 55.0% | 0.03 | 57.5% | 0.03 | 50.0% | 0.03 | 53.1% | 0.03 | 57.7% | 0.03 |
|  | Slightly anxious | 27.3% | 0.02 | 25.7% | 0.02 | 30.6% | 0.03 | 30.2% | 0.03 | 23.1% | 0.02 |
|  | Moderately anxious | 13.0% | 0.02 | 12.6% | 0.02 | 13.9% | 0.02 | 12.5% | 0.02 | 13.8% | 0.02 |
|  | Severely anxious | 3.7% | 0.01 | 3.3% | 0.01 | 4.6% | 0.01 | 3.1% | 0.01 | 4.6% | 0.01 |
|  | Extremely anxious | 0.9% | 0.01 | 0.9% | 0.01 | 0.9% | 0.01 | 1.0% | 0.01 | 0.8% | 0.00 |

* Percentages were not calculated for subgroups lower than 20 participants.

**Abbreviations**. **SE**: Standard Error.

# Supplementary table 2.2.1. Reference norms at 2-year follow-up for the Summary score of EORTC QLQ-C30.

|  |  | **EORTC QLQ-C30: Summary score** | | | | |
| --- | --- | --- | --- | --- | --- | --- |
|  |  |  | **Charlson** | | **TNM stage** | |
|  |  | **All** | **0** | **≥ 1** | **0 – I** | **II - III** |
| **Age (years)**  **<40** | **n** | 35 | 33 | 2* | 14* | 21 |
|  | **Mean (SD)** | 87.2 (13.7) | 87.5 (14.1) | 82.6 (0.0) | 91.5 (10.6) | 84.4 (15.0) |
|  | **95%CI** | [ 82.7 - 91.8 ] | [ 82.7 - 92.3 ] | [ 82.5 - 82.6 ] | [ 86.0 - 97.1 ] | [ 78.0 - 90.8 ] |
|  | **P 5** | 57.0 | 54.5 |  |  | 40.1 |
|  | **P 10** | 68.6 | 67.6 |  |  | 66.6 |
|  | **P 20** | 77.6 | 76.9 |  |  | 72.9 |
|  | **P 25** | 81.6 | 79.9 |  |  | 76.0 |
|  | **P 30** | 82.4 | 82.3 |  |  | 80.0 |
|  | **P 40** | 88.1 | 90.6 |  |  | 82.4 |
|  | **Median** | 91.9 | 92.2 | 82.6 | 96.3 | 86.8 |
|  | **P 60** | 95.2 | 95.8 |  |  | 91.9 |
|  | **P 70** | 96.9 | 97.0 |  |  | 95.0 |
|  | **P 75** | 97.4 | 97.4 |  |  | 96.5 |
|  | **P 80** | 98.3 | 98.5 |  |  | 97.9 |
|  | **P 90** | 99.2 | 99.3 |  |  | 99.3 |
|  | **P 95** | 99.6 | 99.6 |  |  | 99.5 |
| **40-65** | **n** | 722 | 609 | 113 | 482 | 240 |
|  | **Mean (SD)** | 85.1 (14.5) | 86.0 (13.7) | 80.3 (17.2) | 86.3 (13.5) | 82.8 (15.9) |
|  | **95%CI** | [ 84.1 - 86.2 ] | [ 84.9 - 87.1 ] | [ 77.2 - 83.5 ] | [ 85.1 - 87.5 ] | [ 80.8 - 84.8 ] |
|  | **P 5** | 55.5 | 57.0 | 41.8 | 57.8 | 48.2 |
|  | **P 10** | 63.0 | 66.1 | 52.2 | 65.9 | 57.6 |
|  | **P 20** | 75.5 | 76.7 | 67.0 | 78.0 | 72.1 |
|  | **P 25** | 79.0 | 79.8 | 73.5 | 80.7 | 74.2 |
|  | **P 30** | 81.7 | 82.9 | 76.9 | 83.5 | 77.6 |
|  | **P 40** | 85.9 | 86.8 | 81.2 | 87.0 | 83.2 |
|  | **Median** | 89.1 | 89.9 | 84.7 | 89.8 | 86.6 |
|  | **P 60** | 92.3 | 92.9 | 88.1 | 92.9 | 91.1 |
|  | **P 70** | 95.0 | 95.5 | 92.6 | 95.5 | 94.2 |
|  | **P 75** | 96.2 | 96.5 | 93.6 | 96.6 | 95.2 |
|  | **P 80** | 97.2 | 97.4 | 95.0 | 97.4 | 96.2 |
|  | **P 90** | 99.4 | 99.5 | 97.8 | 99.4 | 98.7 |
|  | **P 95** | 100.0 | 100.0 | 99.4 | 100.0 | 100.0 |
| **>65** | **n** | 309 | 201 | 108 | 181 | 128 |
|  | **Mean (SD)** | 84.0 (14.6) | 86.6 (12.0) | 79.1 (17.6) | 85.0 (14.2) | 82.5 (15.1) |
|  | **95%CI** | [ 82.4 - 85.6 ] | [ 85.0 - 88.3 ] | [ 75.7 - 82.4 ] | [ 83.0 - 87.1 ] | [ 79.9 - 85.1 ] |
|  | **P 5** | 57.0 | 62.5 | 40.1 | 61.3 | 52.9 |
|  | **P 10** | 65.0 | 69.5 | 53.6 | 66.8 | 61.0 |
|  | **P 20** | 73.4 | 76.4 | 66.4 | 75.7 | 70.5 |
|  | **P 25** | 76.5 | 79.0 | 70.1 | 77.7 | 74.3 |
|  | **P 30** | 78.6 | 81.8 | 73.2 | 80.5 | 76.9 |
|  | **P 40** | 84.0 | 87.6 | 78.9 | 84.7 | 81.4 |
|  | **Median** | 88.4 | 90.3 | 82.0 | 88.8 | 86.9 |
|  | **P 60** | 91.3 | 92.6 | 85.7 | 92.1 | 90.3 |
|  | **P 70** | 93.6 | 94.6 | 91.0 | 94.6 | 92.8 |
|  | **P 75** | 94.9 | 95.3 | 92.7 | 95.6 | 94.3 |
|  | **P 80** | 95.6 | 96.4 | 94.6 | 97.1 | 95.0 |
|  | **P 90** | 99.0 | 99.3 | 98.7 | 99.3 | 98.5 |
|  | **P 95** | 100.0 | 100.0 | 100.0 | 100.0 | 100.0 |

* Deciles were not calculated for subgroups lower than 20 participants.

**Abbreviations**. **SD**: Standard Deviation; **95% CI**: 95% Confidence Interval; **P**: Percentile.

# Supplementary table 2.2.2. Reference norms at 2-year follow-up for the Physical function of EORTC QLQ-C30.

|  |  | **EORTC QLQ-C30: Physical function** | | | | |
| --- | --- | --- | --- | --- | --- | --- |
|  |  |  | **Charlson** | | **TNM stage** | |
|  |  | **All** | **0** | **≥ 1** | **0 – I** | **II - III** |
| **Age (years)**  **<40** | **n** | 35 | 33 | 2* | 14* | 21 |
|  | **Mean (SD)** | 93.0 (10.6) | 93.5 (10.3) | 83.3 (14.1) | 97.1 (4.3) | 90.2 (12.6) |
|  | **95%CI** | [ 89.4 - 96.5 ] | [ 90.0 - 97.1 ] | [ 63.7 - 102.9 ] | [ 94.9 - 99.4 ] | [ 84.8 - 95.5 ] |
|  | **P 5** | 60.0 | 60.0 |  |  | 60.0 |
|  | **P 10** | 77.3 | 82.7 |  |  | 62.7 |
|  | **P 20** | 86.7 | 86.7 |  |  | 82.7 |
|  | **P 25** | 86.7 | 90.0 |  |  | 86.7 |
|  | **P 30** | 93.3 | 93.3 |  |  | 86.7 |
|  | **P 40** | 93.3 | 93.3 |  |  | 92.0 |
|  | **Median** | 100.0 | 100.0 | 83.3 | 100.0 | 93.3 |
|  | **P 60** | 100.0 | 100.0 |  |  | 100.0 |
|  | **P 70** | 100.0 | 100.0 |  |  | 100.0 |
|  | **P 75** | 100.0 | 100.0 |  |  | 100.0 |
|  | **P 80** | 100.0 | 100.0 |  |  | 100.0 |
|  | **P 90** | 100.0 | 100.0 |  |  | 100.0 |
|  | **P 95** | 100.0 | 100.0 |  |  | 100.0 |
| **40-65** | **n** | 732 | 619 | 113 | 488 | 244 |
|  | **Mean (SD)** | 88.6 (14.6) | 89.7 (13.4) | 82.8 (19.1) | 90.0 (14.0) | 85.9 (15.6) |
|  | **95%CI** | [ 87.6 - 89.7 ] | [ 88.6 - 90.8 ] | [ 79.3 - 86.3 ] | [ 88.8 - 91.2 ] | [ 83.9 - 87.8 ] |
|  | **P 5** | 57.7 | 60.0 | 40.0 | 60.0 | 53.3 |
|  | **P 10** | 66.7 | 73.3 | 53.3 | 66.7 | 66.7 |
|  | **P 20** | 80.0 | 80.0 | 66.7 | 86.0 | 80.0 |
|  | **P 25** | 86.7 | 86.7 | 73.3 | 86.7 | 80.0 |
|  | **P 30** | 86.7 | 86.7 | 80.0 | 86.7 | 80.0 |
|  | **P 40** | 93.3 | 93.3 | 86.7 | 93.3 | 86.7 |
|  | **Median** | 93.3 | 93.3 | 86.7 | 93.3 | 89.2 |
|  | **P 60** | 93.3 | 100.0 | 93.3 | 100.0 | 93.3 |
|  | **P 70** | 100.0 | 100.0 | 93.3 | 100.0 | 93.3 |
|  | **P 75** | 100.0 | 100.0 | 100.0 | 100.0 | 100.0 |
|  | **P 80** | 100.0 | 100.0 | 100.0 | 100.0 | 100.0 |
|  | **P 90** | 100.0 | 100.0 | 100.0 | 100.0 | 100.0 |
|  | **P 95** | 100.0 | 100.0 | 100.0 | 100.0 | 100.0 |
| **>65** | **n** | 318 | 209 | 109 | 189 | 129 |
|  | **Mean (SD)** | 81.0 (21.7) | 85.0 (18.4) | 73.3 (25.3) | 83.8 (19.9) | 76.8 (23.6) |
|  | **95%CI** | [ 78.6 - 83.4 ] | [ 82.5 - 87.5 ] | [ 68.6 - 78.1 ] | [ 81.0 - 86.6 ] | [ 72.7 - 80.9 ] |
|  | **P 5** | 33.3 | 43.3 | 10.0 | 43.3 | 23.3 |
|  | **P 10** | 52.7 | 60.0 | 40.0 | 60.0 | 40.0 |
|  | **P 20** | 66.7 | 73.3 | 53.3 | 73.3 | 60.0 |
|  | **P 25** | 73.3 | 80.0 | 60.0 | 73.3 | 66.7 |
|  | **P 30** | 80.0 | 80.0 | 66.7 | 80.0 | 66.7 |
|  | **P 40** | 82.0 | 86.7 | 73.3 | 86.7 | 80.0 |
|  | **Median** | 86.7 | 93.3 | 80.0 | 86.7 | 86.7 |
|  | **P 60** | 93.3 | 93.3 | 86.7 | 93.3 | 86.7 |
|  | **P 70** | 93.3 | 100.0 | 93.3 | 100.0 | 93.3 |
|  | **P 75** | 100.0 | 100.0 | 93.3 | 100.0 | 93.3 |
|  | **P 80** | 100.0 | 100.0 | 93.3 | 100.0 | 93.3 |
|  | **P 90** | 100.0 | 100.0 | 100.0 | 100.0 | 100.0 |
|  | **P 95** | 100.0 | 100.0 | 100.0 | 100.0 | 100.0 |

* Deciles were not calculated for subgroups lower than 20 participants.

**Abbreviations**. **SD**: Standard Deviation; **95% CI**: 95% Confidence Interval; **P**: Percentile.

# Supplementary table 2.2.3. Reference norms at 2-year follow-up for the Role function of EORTC QLQ-C30.

|  |  | **EORTC QLQ-C30: Role function** | | | | |
| --- | --- | --- | --- | --- | --- | --- |
|  |  |  | **Charlson** | | **TNM stage** | |
|  |  | **All** | **0** | **≥ 1** | **0 – I** | **II - III** |
| **Age (years)**  **<40** | **n** | 35 | 33 | 2* | 14* | 21 |
|  | **Mean (SD)** | 88.6 (20.9) | 89.4 (21.2) | 75.0 (11.8) | 92.9 (12.6) | 85.7 (24.9) |
|  | **95%CI** | [ 81.6 - 95.5 ] | [ 82.2 - 96.6 ] | [ 58.7 - 91.3 ] | [ 86.3 - 99.5 ] | [ 75.1 - 96.4 ] |
|  | **P 5** | 40.0 | 35.0 |  |  | 5.0 |
|  | **P 10** | 66.7 | 66.7 |  |  | 53.3 |
|  | **P 20** | 70.0 | 80.0 |  |  | 66.7 |
|  | **P 25** | 83.3 | 83.3 |  |  | 75.0 |
|  | **P 30** | 83.3 | 86.7 |  |  | 83.3 |
|  | **P 40** | 100.0 | 100.0 |  |  | 96.7 |
|  | **Median** | 100.0 | 100.0 | 75.0 | 100.0 | 100.0 |
|  | **P 60** | 100.0 | 100.0 |  |  | 100.0 |
|  | **P 70** | 100.0 | 100.0 |  |  | 100.0 |
|  | **P 75** | 100.0 | 100.0 |  |  | 100.0 |
|  | **P 80** | 100.0 | 100.0 |  |  | 100.0 |
|  | **P 90** | 100.0 | 100.0 |  |  | 100.0 |
|  | **P 95** | 100.0 | 100.0 |  |  | 100.0 |
| **40-65** | **n** | 735 | 622 | 113 | 490 | 245 |
|  | **Mean (SD)** | 87.4 (21.7) | 88.5 (20.6) | 81.6 (26.7) | 89.2 (20.1) | 83.9 (24.3) |
|  | **95%CI** | [ 85.8 - 89.0 ] | [ 86.9 - 90.1 ] | [ 76.6 - 86.5 ] | [ 87.4 - 91.0 ] | [ 80.8 - 86.9 ] |
|  | **P 5** | 33.3 | 33.3 | 16.7 | 33.3 | 33.3 |
|  | **P 10** | 66.7 | 66.7 | 33.3 | 66.7 | 50.0 |
|  | **P 20** | 66.7 | 66.7 | 66.7 | 83.3 | 66.7 |
|  | **P 25** | 83.3 | 83.3 | 66.7 | 83.3 | 66.7 |
|  | **P 30** | 83.3 | 83.3 | 70.0 | 83.3 | 80.0 |
|  | **P 40** | 100.0 | 100.0 | 83.3 | 100.0 | 83.3 |
|  | **Median** | 100.0 | 100.0 | 100.0 | 100.0 | 100.0 |
|  | **P 60** | 100.0 | 100.0 | 100.0 | 100.0 | 100.0 |
|  | **P 70** | 100.0 | 100.0 | 100.0 | 100.0 | 100.0 |
|  | **P 75** | 100.0 | 100.0 | 100.0 | 100.0 | 100.0 |
|  | **P 80** | 100.0 | 100.0 | 100.0 | 100.0 | 100.0 |
|  | **P 90** | 100.0 | 100.0 | 100.0 | 100.0 | 100.0 |
|  | **P 95** | 100.0 | 100.0 | 100.0 | 100.0 | 100.0 |
| **>65** | **n** | 322 | 212 | 110 | 192 | 130 |
|  | **Mean (SD)** | 82.7 (26.8) | 86.1 (24.3) | 76.1 (30.2) | 84.5 (25.2) | 79.9 (29.0) |
|  | **95%CI** | [ 79.7 - 85.6 ] | [ 82.8 - 89.4 ] | [ 70.4 - 81.7 ] | [ 81.0 - 88.1 ] | [ 74.9 - 84.9 ] |
|  | **P 5** | 16.7 | 33.3 | 0.0 | 33.3 | 9.2 |
|  | **P 10** | 33.3 | 50.0 | 33.3 | 50.0 | 33.3 |
|  | **P 20** | 66.7 | 66.7 | 50.0 | 66.7 | 66.7 |
|  | **P 25** | 66.7 | 83.3 | 66.7 | 66.7 | 66.7 |
|  | **P 30** | 66.7 | 83.3 | 66.7 | 83.3 | 66.7 |
|  | **P 40** | 100.0 | 100.0 | 66.7 | 100.0 | 83.3 |
|  | **Median** | 100.0 | 100.0 | 100.0 | 100.0 | 100.0 |
|  | **P 60** | 100.0 | 100.0 | 100.0 | 100.0 | 100.0 |
|  | **P 70** | 100.0 | 100.0 | 100.0 | 100.0 | 100.0 |
|  | **P 75** | 100.0 | 100.0 | 100.0 | 100.0 | 100.0 |
|  | **P 80** | 100.0 | 100.0 | 100.0 | 100.0 | 100.0 |
|  | **P 90** | 100.0 | 100.0 | 100.0 | 100.0 | 100.0 |
|  | **P 95** | 100.0 | 100.0 | 100.0 | 100.0 | 100.0 |

* Deciles were not calculated for subgroups lower than 20 participants.

**Abbreviations**. **SD**: Standard Deviation; **95% CI**: 95% Confidence Interval; **P**: Percentile.

# Supplementary table 2.2.4. Reference norms at 2-year follow-up for the Emotional function of EORTC QLQ-C30.

|  |  | **EORTC QLQ-C30: Emotional function** | | | | |
| --- | --- | --- | --- | --- | --- | --- |
|  |  |  | **Charlson** | | **TNM stage** | |
|  |  | **All** | **0** | **≥ 1** | **0 – I** | **II - III** |
| **Age (years)**  **<40** | **n** | 35 | 33 | 2* | 14* | 21 |
|  | **Mean (SD)** | 78.1 (24.7) | 77.5 (25.3) | 87.5 (5.9) | 79.8 (27.5) | 77.0 (23.3) |
|  | **95%CI** | [ 69.9 - 86.3 ] | [ 68.9 - 86.2 ] | [ 79.3 - 95.7 ] | [ 65.4 - 94.2 ] | [ 67.0 - 86.9 ] |
|  | **P 5** | 26.7 | 23.3 |  |  | 33.3 |
|  | **P 10** | 38.3 | 36.7 |  |  | 35.0 |
|  | **P 20** | 53.3 | 50.0 |  |  | 45.0 |
|  | **P 25** | 66.7 | 66.7 |  |  | 58.3 |
|  | **P 30** | 73.3 | 68.3 |  |  | 71.7 |
|  | **P 40** | 83.3 | 83.3 |  |  | 81.7 |
|  | **Median** | 83.3 | 83.3 | 87.5 | 91.7 | 83.3 |
|  | **P 60** | 91.7 | 91.7 |  |  | 91.7 |
|  | **P 70** | 91.7 | 91.7 |  |  | 91.7 |
|  | **P 75** | 100.0 | 100.0 |  |  | 95.8 |
|  | **P 80** | 100.0 | 100.0 |  |  | 100.0 |
|  | **P 90** | 100.0 | 100.0 |  |  | 100.0 |
|  | **P 95** | 100.0 | 100.0 |  |  | 100.0 |
| **40-65** | **n** | 735 | 622 | 113 | 490 | 245 |
|  | **Mean (SD)** | 77.8 (23.2) | 78.6 (22.5) | 73.2 (26.2) | 78.0 (23.2) | 77.3 (23.2) |
|  | **95%CI** | [ 76.1 - 79.5 ] | [ 76.9 - 80.4 ] | [ 68.3 - 78.0 ] | [ 76.0 - 80.1 ] | [ 74.4 - 80.2 ] |
|  | **P 5** | 33.3 | 33.3 | 16.7 | 33.3 | 33.3 |
|  | **P 10** | 41.7 | 50.0 | 33.3 | 41.7 | 41.7 |
|  | **P 20** | 66.7 | 66.7 | 50.0 | 66.7 | 58.3 |
|  | **P 25** | 66.7 | 66.7 | 66.7 | 66.7 | 66.7 |
|  | **P 30** | 66.7 | 66.7 | 66.7 | 66.7 | 66.7 |
|  | **P 40** | 75.0 | 75.0 | 75.0 | 75.0 | 75.0 |
|  | **Median** | 83.3 | 83.3 | 75.0 | 83.3 | 83.3 |
|  | **P 60** | 91.7 | 91.7 | 91.7 | 91.7 | 91.7 |
|  | **P 70** | 100.0 | 100.0 | 91.7 | 100.0 | 91.7 |
|  | **P 75** | 100.0 | 100.0 | 91.7 | 100.0 | 100.0 |
|  | **P 80** | 100.0 | 100.0 | 100.0 | 100.0 | 100.0 |
|  | **P 90** | 100.0 | 100.0 | 100.0 | 100.0 | 100.0 |
|  | **P 95** | 100.0 | 100.0 | 100.0 | 100.0 | 100.0 |
| **>65** | **n** | 324 | 216 | 108 | 190 | 134 |
|  | **Mean (SD)** | 79.0 (21.0) | 80.0 (19.8) | 77.0 (23.1) | 79.7 (19.8) | 77.9 (22.6) |
|  | **95%CI** | [ 76.7 - 81.3 ] | [ 77.3 - 82.6 ] | [ 72.6 - 81.3 ] | [ 76.9 - 82.5 ] | [ 74.1 - 81.7 ] |
|  | **P 5** | 41.7 | 41.7 | 33.3 | 41.7 | 33.3 |
|  | **P 10** | 50.0 | 50.0 | 41.7 | 50.0 | 41.7 |
|  | **P 20** | 66.7 | 66.7 | 58.3 | 66.7 | 66.7 |
|  | **P 25** | 66.7 | 66.7 | 66.7 | 66.7 | 66.7 |
|  | **P 30** | 66.7 | 66.7 | 66.7 | 66.7 | 66.7 |
|  | **P 40** | 75.0 | 75.0 | 75.0 | 75.0 | 75.0 |
|  | **Median** | 83.3 | 83.3 | 83.3 | 83.3 | 83.3 |
|  | **P 60** | 91.7 | 91.7 | 91.7 | 91.7 | 91.7 |
|  | **P 70** | 100.0 | 100.0 | 94.2 | 91.7 | 100.0 |
|  | **P 75** | 100.0 | 100.0 | 100.0 | 100.0 | 100.0 |
|  | **P 80** | 100.0 | 100.0 | 100.0 | 100.0 | 100.0 |
|  | **P 90** | 100.0 | 100.0 | 100.0 | 100.0 | 100.0 |
|  | **P 95** | 100.0 | 100.0 | 100.0 | 100.0 | 100.0 |

* Deciles were not calculated for subgroups lower than 20 participants.

**Abbreviations**. **SD**: Standard Deviation; **95% CI**: 95% Confidence Interval; **P**: Percentile.

# Supplementary table 2.2.5. Reference norms at 2-year follow-up for the Cognitive function of EORTC QLQ-C30.

|  |  | **EORTC QLQ-C30: Cognitive function** | | | | |
| --- | --- | --- | --- | --- | --- | --- |
|  |  |  | **Charlson** | | **TNM stage** | |
|  |  | **All** | **0** | **≥ 1** | **0 – I** | **II - III** |
| **Age (years)**  **<40** | **n** | 35 | 33 | 2* | 14* | 21 |
|  | **Mean (SD)** | 87.1 (23.3) | 86.4 (23.7) | 100.0 (0.0) | 86.9 (27.9) | 87.3 (20.3) |
|  | **95%CI** | [ 79.4 - 94.8 ] | [ 78.3 - 94.5 ] | [ 100.0 - 100.0 ] | [ 72.3 - 101.5 ] | [ 78.6 - 96.0 ] |
|  | **P 5** | 26.7 | 23.3 |  |  | 33.3 |
|  | **P 10** | 53.3 | 46.7 |  |  | 40.0 |
|  | **P 20** | 83.3 | 80.0 |  |  | 83.3 |
|  | **P 25** | 83.3 | 83.3 |  |  | 83.3 |
|  | **P 30** | 83.3 | 83.3 |  |  | 83.3 |
|  | **P 40** | 100.0 | 93.3 |  |  | 83.3 |
|  | **Median** | 100.0 | 100.0 | 100.0 | 100.0 | 100.0 |
|  | **P 60** | 100.0 | 100.0 |  |  | 100.0 |
|  | **P 70** | 100.0 | 100.0 |  |  | 100.0 |
|  | **P 75** | 100.0 | 100.0 |  |  | 100.0 |
|  | **P 80** | 100.0 | 100.0 |  |  | 100.0 |
|  | **P 90** | 100.0 | 100.0 |  |  | 100.0 |
|  | **P 95** | 100.0 | 100.0 |  |  | 100.0 |
| **40-65** | **n** | 735 | 622 | 113 | 490 | 245 |
|  | **Mean (SD)** | 83.9 (22.7) | 84.4 (22.2) | 81.1 (24.9) | 84.9 (21.9) | 81.7 (24.0) |
|  | **95%CI** | [ 82.2 - 85.5 ] | [ 82.6 - 86.1 ] | [ 76.5 - 85.7 ] | [ 83.0 - 86.9 ] | [ 78.7 - 84.7 ] |
|  | **P 5** | 33.3 | 33.3 | 16.7 | 33.3 | 33.3 |
|  | **P 10** | 50.0 | 50.0 | 50.0 | 50.0 | 50.0 |
|  | **P 20** | 66.7 | 66.7 | 66.7 | 66.7 | 66.7 |
|  | **P 25** | 66.7 | 83.3 | 66.7 | 83.3 | 66.7 |
|  | **P 30** | 83.3 | 83.3 | 83.3 | 83.3 | 83.3 |
|  | **P 40** | 83.3 | 83.3 | 83.3 | 83.3 | 83.3 |
|  | **Median** | 100.0 | 100.0 | 83.3 | 100.0 | 83.3 |
|  | **P 60** | 100.0 | 100.0 | 100.0 | 100.0 | 100.0 |
|  | **P 70** | 100.0 | 100.0 | 100.0 | 100.0 | 100.0 |
|  | **P 75** | 100.0 | 100.0 | 100.0 | 100.0 | 100.0 |
|  | **P 80** | 100.0 | 100.0 | 100.0 | 100.0 | 100.0 |
|  | **P 90** | 100.0 | 100.0 | 100.0 | 100.0 | 100.0 |
|  | **P 95** | 100.0 | 100.0 | 100.0 | 100.0 | 100.0 |
| **>65** | **n** | 326 | 216 | 110 | 193 | 133 |
|  | **Mean (SD)** | 84.1 (20.6) | 85.9 (19.2) | 80.6 (23.0) | 84.0 (19.9) | 84.2 (21.7) |
|  | **95%CI** | [ 81.9 - 86.3 ] | [ 83.3 - 88.4 ] | [ 76.3 - 84.9 ] | [ 81.2 - 86.8 ] | [ 80.5 - 87.9 ] |
|  | **P 5** | 33.3 | 47.5 | 33.3 | 33.3 | 33.3 |
|  | **P 10** | 50.0 | 50.0 | 35.0 | 50.0 | 50.0 |
|  | **P 20** | 66.7 | 66.7 | 66.7 | 66.7 | 66.7 |
|  | **P 25** | 83.3 | 83.3 | 66.7 | 83.3 | 66.7 |
|  | **P 30** | 83.3 | 83.3 | 83.3 | 83.3 | 83.3 |
|  | **P 40** | 83.3 | 83.3 | 83.3 | 83.3 | 83.3 |
|  | **Median** | 83.3 | 100.0 | 83.3 | 83.3 | 100.0 |
|  | **P 60** | 100.0 | 100.0 | 100.0 | 100.0 | 100.0 |
|  | **P 70** | 100.0 | 100.0 | 100.0 | 100.0 | 100.0 |
|  | **P 75** | 100.0 | 100.0 | 100.0 | 100.0 | 100.0 |
|  | **P 80** | 100.0 | 100.0 | 100.0 | 100.0 | 100.0 |
|  | **P 90** | 100.0 | 100.0 | 100.0 | 100.0 | 100.0 |
|  | **P 95** | 100.0 | 100.0 | 100.0 | 100.0 | 100.0 |

* Deciles were not calculated for subgroups lower than 20 participants.

**Abbreviations. SD:** Standard Deviation; **95% CI:** 95% Confidence Interval; **P:** Percentile.

# Supplementary table 2.2.6. Reference norms at 2-year follow-up for the Social function of EORTC QLQ-C30.

|  |  | **EORTC QLQ-C30: Social function** | | | | |
| --- | --- | --- | --- | --- | --- | --- |
|  |  |  | **Charlson** | | **TNM stage** | |
|  |  | **All** | **0** | **≥ 1** | **0 – I** | **II - III** |
| **Age (years)**  **<40** | **n** | 35 | 33 | 2* | 14* | 21 |
|  | **Mean (SD)** | 84.8 (19.5) | 84.8 (19.7) | 83.3 (23.6) | 91.7 (14.2) | 80.2 (21.5) |
|  | **95%CI** | [ 78.3 - 91.2 ] | [ 78.1 - 91.6 ] | [ 50.7 - 116.0 ] | [ 84.2 - 99.1 ] | [ 71.0 - 89.3 ] |
|  | **P 5** | 46.7 | 45.0 |  |  | 35.0 |
|  | **P 10** | 50.0 | 50.0 |  |  | 50.0 |
|  | **P 20** | 66.7 | 66.7 |  |  | 56.7 |
|  | **P 25** | 66.7 | 66.7 |  |  | 66.7 |
|  | **P 30** | 66.7 | 70.0 |  |  | 66.7 |
|  | **P 40** | 83.3 | 83.3 |  |  | 80.0 |
|  | **Median** | 100.0 | 100.0 | 83.3 | 100.0 | 83.3 |
|  | **P 60** | 100.0 | 100.0 |  |  | 100.0 |
|  | **P 70** | 100.0 | 100.0 |  |  | 100.0 |
|  | **P 75** | 100.0 | 100.0 |  |  | 100.0 |
|  | **P 80** | 100.0 | 100.0 |  |  | 100.0 |
|  | **P 90** | 100.0 | 100.0 |  |  | 100.0 |
|  | **P 95** | 100.0 | 100.0 |  |  | 100.0 |
| **40-65** | **n** | 735 | 622 | 113 | 490 | 245 |
|  | **Mean (SD)** | 85.0 (24.2) | 86.0 (23.2) | 79.1 (28.2) | 87.1 (21.9) | 80.7 (27.7) |
|  | **95%CI** | [ 83.2 - 86.7 ] | [ 84.2 - 87.9 ] | [ 73.8 - 84.3 ] | [ 85.1 - 89.0 ] | [ 77.3 - 84.2 ] |
|  | **P 5** | 33.3 | 33.3 | 11.7 | 33.3 | 16.7 |
|  | **P 10** | 50.0 | 50.0 | 33.3 | 50.0 | 33.3 |
|  | **P 20** | 66.7 | 66.7 | 63.3 | 66.7 | 66.7 |
|  | **P 25** | 66.7 | 83.3 | 66.7 | 83.3 | 66.7 |
|  | **P 30** | 83.3 | 83.3 | 66.7 | 83.3 | 66.7 |
|  | **P 40** | 100.0 | 100.0 | 83.3 | 100.0 | 83.3 |
|  | **Median** | 100.0 | 100.0 | 100.0 | 100.0 | 100.0 |
|  | **P 60** | 100.0 | 100.0 | 100.0 | 100.0 | 100.0 |
|  | **P 70** | 100.0 | 100.0 | 100.0 | 100.0 | 100.0 |
|  | **P 75** | 100.0 | 100.0 | 100.0 | 100.0 | 100.0 |
|  | **P 80** | 100.0 | 100.0 | 100.0 | 100.0 | 100.0 |
|  | **P 90** | 100.0 | 100.0 | 100.0 | 100.0 | 100.0 |
|  | **P 95** | 100.0 | 100.0 | 100.0 | 100.0 | 100.0 |
| **>65** | **n** | 323 | 214 | 109 | 190 | 133 |
|  | **Mean (SD)** | 88.0 (22.8) | 90.7 (19.9) | 82.9 (27.0) | 89.8 (19.7) | 85.5 (26.5) |
|  | **95%CI** | [ 85.5 - 90.5 ] | [ 88.0 - 93.3 ] | [ 77.8 - 87.9 ] | [ 87.0 - 92.6 ] | [ 81.0 - 90.0 ] |
|  | **P 5** | 33.3 | 50.0 | 16.7 | 50.0 | 28.3 |
|  | **P 10** | 50.0 | 66.7 | 33.3 | 66.7 | 40.0 |
|  | **P 20** | 83.3 | 83.3 | 66.7 | 83.3 | 66.7 |
|  | **P 25** | 83.3 | 83.3 | 66.7 | 83.3 | 83.3 |
|  | **P 30** | 83.3 | 100.0 | 83.3 | 88.3 | 83.3 |
|  | **P 40** | 100.0 | 100.0 | 83.3 | 100.0 | 100.0 |
|  | **Median** | 100.0 | 100.0 | 100.0 | 100.0 | 100.0 |
|  | **P 60** | 100.0 | 100.0 | 100.0 | 100.0 | 100.0 |
|  | **P 70** | 100.0 | 100.0 | 100.0 | 100.0 | 100.0 |
|  | **P 75** | 100.0 | 100.0 | 100.0 | 100.0 | 100.0 |
|  | **P 80** | 100.0 | 100.0 | 100.0 | 100.0 | 100.0 |
|  | **P 90** | 100.0 | 100.0 | 100.0 | 100.0 | 100.0 |
|  | **P 95** | 100.0 | 100.0 | 100.0 | 100.0 | 100.0 |

* Deciles were not calculated for subgroups lower than 20 participants.

**Abbreviations. SD:** Standard Deviation; **95% CI:** 95% Confidence Interval; **P:** Percentile.

# Supplementary table 2.2.7. Reference norms at 2-year follow-up for the Global health status of EORTC QLQ-C30.

|  |  | **EORTC QLQ-C30: Global health status** | | | | |
| --- | --- | --- | --- | --- | --- | --- |
|  |  |  | **Charlson** | | **TNM stage** | |
|  |  | **All** | **0** | **≥ 1** | **0 – I** | **II - III** |
| **Age (years)**  **<40** | **n** | 35 | 33 | 2* | 14* | 21 |
|  | **Mean (SD)** | 77.1 (17.0) | 77.8 (16.8) | 66.7 (23.6) | 81.5 (13.5) | 74.2 (18.6) |
|  | **95%CI** | [ 71.5 - 82.8 ] | [ 72.1 - 83.5 ] | [ 34.0 - 99.3 ] | [ 74.5 - 88.6 ] | [ 66.2 - 82.2 ] |
|  | **P 5** | 46.7 | 45.0 |  |  | 35.0 |
|  | **P 10** | 50.0 | 50.0 |  |  | 50.0 |
|  | **P 20** | 58.3 | 58.3 |  |  | 53.3 |
|  | **P 25** | 66.7 | 66.7 |  |  | 58.3 |
|  | **P 30** | 73.3 | 75.0 |  |  | 63.3 |
|  | **P 40** | 83.3 | 83.3 |  |  | 73.3 |
|  | **Median** | 83.3 | 83.3 | 66.7 | 83.3 | 83.3 |
|  | **P 60** | 83.3 | 83.3 |  |  | 83.3 |
|  | **P 70** | 83.3 | 83.3 |  |  | 83.3 |
|  | **P 75** | 83.3 | 83.3 |  |  | 83.3 |
|  | **P 80** | 90.0 | 91.7 |  |  | 88.3 |
|  | **P 90** | 100.0 | 100.0 |  |  | 100.0 |
|  | **P 95** | 100.0 | 100.0 |  |  | 100.0 |
| **40-65** | **n** | 734 | 621 | 113 | 490 | 244 |
|  | **Mean (SD)** | 73.6 (19.9) | 75.1 (19.0) | 65.6 (23.0) | 74.9 (18.9) | 71.0 (21.7) |
|  | **95%CI** | [ 72.2 - 75.0 ] | [ 73.6 - 76.5 ] | [ 61.4 - 69.9 ] | [ 73.2 - 76.6 ] | [ 68.3 - 73.8 ] |
|  | **P 5** | 33.3 | 41.7 | 16.7 | 41.7 | 33.3 |
|  | **P 10** | 50.0 | 50.0 | 33.3 | 50.0 | 41.7 |
|  | **P 20** | 58.3 | 66.7 | 50.0 | 66.7 | 50.0 |
|  | **P 25** | 66.7 | 66.7 | 50.0 | 66.7 | 58.3 |
|  | **P 30** | 66.7 | 66.7 | 50.0 | 66.7 | 66.7 |
|  | **P 40** | 66.7 | 75.0 | 66.7 | 66.7 | 66.7 |
|  | **Median** | 83.3 | 83.3 | 66.7 | 83.3 | 75.0 |
|  | **P 60** | 83.3 | 83.3 | 66.7 | 83.3 | 83.3 |
|  | **P 70** | 83.3 | 83.3 | 83.3 | 83.3 | 83.3 |
|  | **P 75** | 83.3 | 83.3 | 83.3 | 83.3 | 83.3 |
|  | **P 80** | 83.3 | 91.7 | 83.3 | 91.7 | 83.3 |
|  | **P 90** | 100.0 | 100.0 | 100.0 | 100.0 | 100.0 |
|  | **P 95** | 100.0 | 100.0 | 100.0 | 100.0 | 100.0 |
| **>65** | **n** | 327 | 217 | 110 | 193 | 134 |
|  | **Mean (SD)** | 69.6 (22.0) | 72.7 (21.1) | 63.5 (22.6) | 71.0 (21.2) | 67.5 (23.0) |
|  | **95%CI** | [ 67.2 - 72.0 ] | [ 69.9 - 75.5 ] | [ 59.3 - 67.7 ] | [ 68.0 - 74.0 ] | [ 63.6 - 71.4 ] |
|  | **P 5** | 33.3 | 33.3 | 16.7 | 33.3 | 31.3 |
|  | **P 10** | 41.7 | 48.3 | 33.3 | 50.0 | 33.3 |
|  | **P 20** | 50.0 | 55.0 | 50.0 | 50.0 | 50.0 |
|  | **P 25** | 50.0 | 66.7 | 50.0 | 54.2 | 50.0 |
|  | **P 30** | 66.7 | 66.7 | 50.0 | 66.7 | 58.3 |
|  | **P 40** | 66.7 | 66.7 | 58.3 | 66.7 | 66.7 |
|  | **Median** | 66.7 | 75.0 | 66.7 | 75.0 | 66.7 |
|  | **P 60** | 83.3 | 83.3 | 66.7 | 83.3 | 75.0 |
|  | **P 70** | 83.3 | 83.3 | 75.0 | 83.3 | 83.3 |
|  | **P 75** | 83.3 | 83.3 | 83.3 | 83.3 | 83.3 |
|  | **P 80** | 83.3 | 91.7 | 83.3 | 91.7 | 83.3 |
|  | **P 90** | 100.0 | 100.0 | 91.7 | 100.0 | 100.0 |
|  | **P 95** | 100.0 | 100.0 | 100.0 | 100.0 | 100.0 |

* Deciles were not calculated for subgroups lower than 20 participants.

**Abbreviations. SD:** Standard Deviation; **95% CI:** 95% Confidence Interval; **P:** Percentile.

# Supplementary table 2.2.8. Reference norms at 2-year follow-up for the Fatigue of EORTC QLQ-C30.

|  |  | **EORTC QLQ-C30: Fatigue** | | | | |
| --- | --- | --- | --- | --- | --- | --- |
|  |  |  | **Charlson** | | **TNM stage** | |
|  |  | **All** | **0** | **≥ 1** | **0 – I** | **II - III** |
| **Age (years)**  **<40** | **n** | 35 | 33 | 2* | 14* | 21 |
|  | **Mean (SD)** | 21.1 (19.7) | 20.0 (19.7) | 38.9 (7.9) | 13.5 (20.1) | 26.2 (18.2) |
|  | **95%CI** | [ 14.6 - 27.6 ] | [ 13.3 - 26.8 ] | [ 28.0 - 49.8 ] | [ 3.0 - 24.0 ] | [ 18.4 - 34.0 ] |
|  | **P 5** | 0.0 | 0.0 |  |  | 0.0 |
|  | **P 10** | 0.0 | 0.0 |  |  | 2.2 |
|  | **P 20** | 0.0 | 0.0 |  |  | 11.1 |
|  | **P 25** | 0.0 | 0.0 |  |  | 11.1 |
|  | **P 30** | 8.9 | 2.2 |  |  | 17.8 |
|  | **P 40** | 11.1 | 11.1 |  |  | 22.2 |
|  | **Median** | 22.2 | 22.2 | 38.9 | 0.0 | 22.2 |
|  | **P 60** | 22.2 | 22.2 |  |  | 33.3 |
|  | **P 70** | 33.3 | 31.1 |  |  | 33.3 |
|  | **P 75** | 33.3 | 33.3 |  |  | 33.3 |
|  | **P 80** | 33.3 | 33.3 |  |  | 33.3 |
|  | **P 90** | 44.4 | 44.4 |  |  | 44.4 |
|  | **P 95** | 61.1 | 63.9 |  |  | 79.4 |
| **40-65** | **n** | 733 | 620 | 113 | 489 | 244 |
|  | **Mean (SD)** | 24.7 (24.2) | 23.4 (23.5) | 31.6 (27.0) | 23.7 (23.9) | 26.6 (24.8) |
|  | **95%CI** | [ 22.9 - 26.4 ] | [ 21.6 - 25.3 ] | [ 26.6 - 36.5 ] | [ 21.6 - 25.8 ] | [ 23.5 - 29.8 ] |
|  | **P 5** | 0.0 | 0.0 | 0.0 | 0.0 | 0.0 |
|  | **P 10** | 0.0 | 0.0 | 0.0 | 0.0 | 0.0 |
|  | **P 20** | 0.0 | 0.0 | 0.0 | 0.0 | 0.0 |
|  | **P 25** | 0.0 | 0.0 | 11.1 | 0.0 | 0.0 |
|  | **P 30** | 0.0 | 0.0 | 11.1 | 0.0 | 11.1 |
|  | **P 40** | 11.1 | 11.1 | 22.2 | 11.1 | 22.2 |
|  | **Median** | 22.2 | 22.2 | 22.2 | 22.2 | 22.2 |
|  | **P 60** | 26.7 | 22.2 | 33.3 | 22.2 | 33.3 |
|  | **P 70** | 33.3 | 33.3 | 44.4 | 33.3 | 33.3 |
|  | **P 75** | 33.3 | 33.3 | 50.0 | 33.3 | 33.3 |
|  | **P 80** | 44.4 | 33.3 | 55.6 | 44.4 | 44.4 |
|  | **P 90** | 66.7 | 55.6 | 66.7 | 55.6 | 66.7 |
|  | **P 95** | 77.8 | 66.7 | 88.9 | 66.7 | 77.8 |
| **>65** | **n** | 320 | 210 | 110 | 190 | 130 |
|  | **Mean (SD)** | 27.2 (25.0) | 22.6 (22.5) | 35.9 (27.1) | 25.7 (24.2) | 29.3 (26.0) |
|  | **95%CI** | [ 24.4 - 29.9 ] | [ 19.6 - 25.7 ] | [ 30.8 - 40.9 ] | [ 22.3 - 29.1 ] | [ 24.8 - 33.8 ] |
|  | **P 5** | 0.0 | 0.0 | 0.0 | 0.0 | 0.0 |
|  | **P 10** | 0.0 | 0.0 | 0.0 | 0.0 | 0.0 |
|  | **P 20** | 0.0 | 0.0 | 11.1 | 0.0 | 0.0 |
|  | **P 25** | 0.0 | 0.0 | 11.1 | 0.0 | 11.1 |
|  | **P 30** | 11.1 | 0.0 | 22.2 | 11.1 | 11.1 |
|  | **P 40** | 22.2 | 11.1 | 22.2 | 11.1 | 22.2 |
|  | **Median** | 22.2 | 22.2 | 33.3 | 22.2 | 22.2 |
|  | **P 60** | 33.3 | 22.2 | 33.3 | 33.3 | 33.3 |
|  | **P 70** | 33.3 | 33.3 | 44.4 | 33.3 | 44.4 |
|  | **P 75** | 44.4 | 33.3 | 55.6 | 33.3 | 44.4 |
|  | **P 80** | 44.4 | 44.4 | 55.6 | 44.4 | 55.6 |
|  | **P 90** | 66.7 | 55.6 | 77.8 | 65.6 | 66.7 |
|  | **P 95** | 77.8 | 66.7 | 88.9 | 77.8 | 82.8 |

* Deciles were not calculated for subgroups lower than 20 participants.

**Abbreviations. SD:** Standard Deviation; **95% CI:** 95% Confidence Interval; **P:** Percentile.

# Supplementary table 2.2.9. Reference norms at 2-year follow-up for the Nausea of EORTC QLQ-C30.

|  |  | **EORTC QLQ-C30: Nausea** | | | | |
| --- | --- | --- | --- | --- | --- | --- |
|  |  |  | **Charlson** | | **TNM stage** | |
|  |  | **All** | **0** | **≥ 1** | **0 – I** | **II - III** |
| **Age (years)**  **<40** | **n** | 35 | 33 | 2* | 14* | 21 |
|  | **Mean (SD)** | 1.0 (3.9) | 1.0 (4.0) | 0.0 (0.0) | 0.0 (0.0) | 1.6 (5.0) |
|  | **95%CI** | [ -0.3 - 2.3 ] | [ -0.4 - 2.4 ] | [ 0.0 - 0.0 ] | [ 0.0 - 0.0 ] | [ -0.6 - 3.7 ] |
|  | **P 5** | 0.0 | 0.0 |  |  | 0.0 |
|  | **P 10** | 0.0 | 0.0 |  |  | 0.0 |
|  | **P 20** | 0.0 | 0.0 |  |  | 0.0 |
|  | **P 25** | 0.0 | 0.0 |  |  | 0.0 |
|  | **P 30** | 0.0 | 0.0 |  |  | 0.0 |
|  | **P 40** | 0.0 | 0.0 |  |  | 0.0 |
|  | **Median** | 0.0 | 0.0 | 0.0 | 0.0 | 0.0 |
|  | **P 60** | 0.0 | 0.0 |  |  | 0.0 |
|  | **P 70** | 0.0 | 0.0 |  |  | 0.0 |
|  | **P 75** | 0.0 | 0.0 |  |  | 0.0 |
|  | **P 80** | 0.0 | 0.0 |  |  | 0.0 |
|  | **P 90** | 0.0 | 0.0 |  |  | 13.3 |
|  | **P 95** | 16.7 | 16.7 |  |  | 16.7 |
| **40-65** | **n** | 735 | 622 | 113 | 490 | 245 |
|  | **Mean (SD)** | 3.3 (10.2) | 3.1 (10.1) | 4.0 (11.0) | 2.7 (9.1) | 4.4 (12.1) |
|  | **95%CI** | [ 2.5 - 4.0 ] | [ 2.3 - 3.9 ] | [ 2.0 - 6.0 ] | [ 1.9 - 3.5 ] | [ 2.9 - 5.9 ] |
|  | **P 5** | 0.0 | 0.0 | 0.0 | 0.0 | 0.0 |
|  | **P 10** | 0.0 | 0.0 | 0.0 | 0.0 | 0.0 |
|  | **P 20** | 0.0 | 0.0 | 0.0 | 0.0 | 0.0 |
|  | **P 25** | 0.0 | 0.0 | 0.0 | 0.0 | 0.0 |
|  | **P 30** | 0.0 | 0.0 | 0.0 | 0.0 | 0.0 |
|  | **P 40** | 0.0 | 0.0 | 0.0 | 0.0 | 0.0 |
|  | **Median** | 0.0 | 0.0 | 0.0 | 0.0 | 0.0 |
|  | **P 60** | 0.0 | 0.0 | 0.0 | 0.0 | 0.0 |
|  | **P 70** | 0.0 | 0.0 | 0.0 | 0.0 | 0.0 |
|  | **P 75** | 0.0 | 0.0 | 0.0 | 0.0 | 0.0 |
|  | **P 80** | 0.0 | 0.0 | 0.0 | 0.0 | 0.0 |
|  | **P 90** | 16.7 | 16.7 | 16.7 | 0.0 | 16.7 |
|  | **P 95** | 33.3 | 33.3 | 33.3 | 24.2 | 33.3 |
| **>65** | **n** | 324 | 215 | 109 | 191 | 133 |
|  | **Mean (SD)** | 3.7 (12.7) | 2.5 (9.1) | 6.0 (17.6) | 2.9 (10.8) | 4.8 (15.0) |
|  | **95%CI** | [ 2.3 - 5.0 ] | [ 1.3 - 3.7 ] | [ 2.7 - 9.3 ] | [ 1.4 - 4.4 ] | [ 2.2 - 7.3 ] |
|  | **P 5** | 0.0 | 0.0 | 0.0 | 0.0 | 0.0 |
|  | **P 10** | 0.0 | 0.0 | 0.0 | 0.0 | 0.0 |
|  | **P 20** | 0.0 | 0.0 | 0.0 | 0.0 | 0.0 |
|  | **P 25** | 0.0 | 0.0 | 0.0 | 0.0 | 0.0 |
|  | **P 30** | 0.0 | 0.0 | 0.0 | 0.0 | 0.0 |
|  | **P 40** | 0.0 | 0.0 | 0.0 | 0.0 | 0.0 |
|  | **Median** | 0.0 | 0.0 | 0.0 | 0.0 | 0.0 |
|  | **P 60** | 0.0 | 0.0 | 0.0 | 0.0 | 0.0 |
|  | **P 70** | 0.0 | 0.0 | 0.0 | 0.0 | 0.0 |
|  | **P 75** | 0.0 | 0.0 | 0.0 | 0.0 | 0.0 |
|  | **P 80** | 0.0 | 0.0 | 0.0 | 0.0 | 0.0 |
|  | **P 90** | 16.7 | 0.0 | 16.7 | 0.0 | 16.7 |
|  | **P 95** | 33.3 | 16.7 | 58.3 | 33.3 | 33.3 |

* Deciles were not calculated for subgroups lower than 20 participants.

**Abbreviations. SD:** Standard Deviation; **95% CI:** 95% Confidence Interval; **P:** Percentile.

# Supplementary table 2.2.10. Reference norms at 2-year follow-up for the Pain of EORTC QLQ-C30.

|  |  | **EORTC QLQ-C30: Pain** | | | | |
| --- | --- | --- | --- | --- | --- | --- |
|  |  |  | **Charlson** | | **TNM stage** | |
|  |  | **All** | **0** | **≥ 1** | **0 – I** | **II - III** |
| **Age (years)**  **<40** | **n** | 35 | 33 | 2* | 14* | 21 |
|  | **Mean (SD)** | 17.1 (23.0) | 16.2 (23.4) | 33.3 (0.0) | 9.5 (14.2) | 22.2 (26.5) |
|  | **95%CI** | [ 9.5 - 24.8 ] | [ 8.2 - 24.1 ] | [ 33.3 - 33.3 ] | [ 2.1 - 17.0 ] | [ 10.9 - 33.6 ] |
|  | **P 5** | 0.0 | 0.0 |  |  | 0.0 |
|  | **P 10** | 0.0 | 0.0 |  |  | 0.0 |
|  | **P 20** | 0.0 | 0.0 |  |  | 0.0 |
|  | **P 25** | 0.0 | 0.0 |  |  | 0.0 |
|  | **P 30** | 0.0 | 0.0 |  |  | 0.0 |
|  | **P 40** | 0.0 | 0.0 |  |  | 0.0 |
|  | **Median** | 0.0 | 0.0 | 33.3 | 0.0 | 16.7 |
|  | **P 60** | 16.7 | 16.7 |  |  | 33.3 |
|  | **P 70** | 33.3 | 30.0 |  |  | 33.3 |
|  | **P 75** | 33.3 | 33.3 |  |  | 33.3 |
|  | **P 80** | 33.3 | 33.3 |  |  | 33.3 |
|  | **P 90** | 40.0 | 43.3 |  |  | 63.3 |
|  | **P 95** | 73.3 | 76.7 |  |  | 96.7 |
| **40-65** | **n** | 736 | 623 | 113 | 491 | 245 |
|  | **Mean (SD)** | 21.0 (24.2) | 20.3 (23.5) | 25.4 (27.4) | 18.8 (23.1) | 25.5 (25.5) |
|  | **95%CI** | [ 19.3 - 22.8 ] | [ 18.4 - 22.1 ] | [ 20.3 - 30.4 ] | [ 16.8 - 20.9 ] | [ 22.3 - 28.7 ] |
|  | **P 5** | 0.0 | 0.0 | 0.0 | 0.0 | 0.0 |
|  | **P 10** | 0.0 | 0.0 | 0.0 | 0.0 | 0.0 |
|  | **P 20** | 0.0 | 0.0 | 0.0 | 0.0 | 0.0 |
|  | **P 25** | 0.0 | 0.0 | 0.0 | 0.0 | 0.0 |
|  | **P 30** | 0.0 | 0.0 | 0.0 | 0.0 | 0.0 |
|  | **P 40** | 0.0 | 0.0 | 16.7 | 0.0 | 16.7 |
|  | **Median** | 16.7 | 16.7 | 16.7 | 16.7 | 16.7 |
|  | **P 60** | 16.7 | 16.7 | 33.3 | 16.7 | 33.3 |
|  | **P 70** | 33.3 | 33.3 | 33.3 | 33.3 | 33.3 |
|  | **P 75** | 33.3 | 33.3 | 33.3 | 33.3 | 33.3 |
|  | **P 80** | 33.3 | 33.3 | 50.0 | 33.3 | 33.3 |
|  | **P 90** | 50.0 | 50.0 | 66.7 | 50.0 | 66.7 |
|  | **P 95** | 66.7 | 66.7 | 83.3 | 66.7 | 83.3 |
| **>65** | **n** | 325 | 215 | 110 | 191 | 134 |
|  | **Mean (SD)** | 22.2 (25.1) | 17.9 (21.9) | 30.5 (28.7) | 20.7 (24.4) | 24.3 (26.1) |
|  | **95%CI** | [ 19.4 - 24.9 ] | [ 15.0 - 20.8 ] | [ 25.1 - 35.8 ] | [ 17.2 - 24.1 ] | [ 19.8 - 28.7 ] |
|  | **P 5** | 0.0 | 0.0 | 0.0 | 0.0 | 0.0 |
|  | **P 10** | 0.0 | 0.0 | 0.0 | 0.0 | 0.0 |
|  | **P 20** | 0.0 | 0.0 | 0.0 | 0.0 | 0.0 |
|  | **P 25** | 0.0 | 0.0 | 0.0 | 0.0 | 0.0 |
|  | **P 30** | 0.0 | 0.0 | 16.7 | 0.0 | 0.0 |
|  | **P 40** | 0.0 | 0.0 | 16.7 | 0.0 | 16.7 |
|  | **Median** | 16.7 | 16.7 | 33.3 | 16.7 | 16.7 |
|  | **P 60** | 16.7 | 16.7 | 33.3 | 16.7 | 33.3 |
|  | **P 70** | 33.3 | 33.3 | 33.3 | 33.3 | 33.3 |
|  | **P 75** | 33.3 | 33.3 | 50.0 | 33.3 | 33.3 |
|  | **P 80** | 33.3 | 33.3 | 50.0 | 33.3 | 33.3 |
|  | **P 90** | 66.7 | 50.0 | 81.7 | 63.3 | 66.7 |
|  | **P 95** | 83.3 | 66.7 | 90.8 | 66.7 | 83.3 |

* Deciles were not calculated for subgroups lower than 20 participants.

**Abbreviations. SD:** Standard Deviation; **95% CI:** 95% Confidence Interval; **P:** Percentile.

# Supplementary table 2.2.11. Reference norms at 2-year follow-up for the Dyspnea of EORTC QLQ-C30.

|  |  | **EORTC QLQ-C30: Dyspnea** | | | | |
| --- | --- | --- | --- | --- | --- | --- |
|  |  |  | **Charlson** | | **TNM stage** | |
|  |  | **All** | **0** | **≥ 1** | **0 – I** | **II - III** |
| **Age (years)**  **<40** | **n** | 35 | 33 | 2* | 14* | 21 |
|  | **Mean (SD)** | 11.4 (22.8) | 12.1 (23.3) | 0.0 (0.0) | 4.8 (12.1) | 15.9 (27.1) |
|  | **95%CI** | [ 3.9 - 19.0 ] | [ 4.2 - 20.1 ] | [ 0.0 - 0.0 ] | [ -1.6 - 11.1 ] | [ 4.3 - 27.5 ] |
|  | **P 5** | 0.0 | 0.0 |  |  | 0.0 |
|  | **P 10** | 0.0 | 0.0 |  |  | 0.0 |
|  | **P 20** | 0.0 | 0.0 |  |  | 0.0 |
|  | **P 25** | 0.0 | 0.0 |  |  | 0.0 |
|  | **P 30** | 0.0 | 0.0 |  |  | 0.0 |
|  | **P 40** | 0.0 | 0.0 |  |  | 0.0 |
|  | **Median** | 0.0 | 0.0 | 0.0 | 0.0 | 0.0 |
|  | **P 60** | 0.0 | 0.0 |  |  | 0.0 |
|  | **P 70** | 0.0 | 0.0 |  |  | 33.3 |
|  | **P 75** | 33.3 | 33.3 |  |  | 33.3 |
|  | **P 80** | 33.3 | 33.3 |  |  | 33.3 |
|  | **P 90** | 33.3 | 33.3 |  |  | 60.0 |
|  | **P 95** | 73.3 | 76.7 |  |  | 96.7 |
| **40-65** | **n** | 731 | 618 | 113 | 489 | 242 |
|  | **Mean (SD)** | 12.3 (21.7) | 10.8 (20.4) | 20.4 (26.1) | 11.2 (20.2) | 14.5 (24.2) |
|  | **95%CI** | [ 10.7 - 13.8 ] | [ 9.2 - 12.4 ] | [ 15.5 - 25.2 ] | [ 9.4 - 13.0 ] | [ 11.4 - 17.5 ] |
|  | **P 5** | 0.0 | 0.0 | 0.0 | 0.0 | 0.0 |
|  | **P 10** | 0.0 | 0.0 | 0.0 | 0.0 | 0.0 |
|  | **P 20** | 0.0 | 0.0 | 0.0 | 0.0 | 0.0 |
|  | **P 25** | 0.0 | 0.0 | 0.0 | 0.0 | 0.0 |
|  | **P 30** | 0.0 | 0.0 | 0.0 | 0.0 | 0.0 |
|  | **P 40** | 0.0 | 0.0 | 0.0 | 0.0 | 0.0 |
|  | **Median** | 0.0 | 0.0 | 0.0 | 0.0 | 0.0 |
|  | **P 60** | 0.0 | 0.0 | 33.3 | 0.0 | 0.0 |
|  | **P 70** | 0.0 | 0.0 | 33.3 | 0.0 | 33.3 |
|  | **P 75** | 33.3 | 33.3 | 33.3 | 33.3 | 33.3 |
|  | **P 80** | 33.3 | 33.3 | 33.3 | 33.3 | 33.3 |
|  | **P 90** | 33.3 | 33.3 | 66.7 | 33.3 | 56.7 |
|  | **P 95** | 66.7 | 66.7 | 66.7 | 66.7 | 66.7 |
| **>65** | **n** | 317 | 209 | 108 | 187 | 130 |
|  | **Mean (SD)** | 9.6 (20.8) | 7.2 (16.9) | 14.2 (26.3) | 10.0 (20.3) | 9.0 (21.5) |
|  | **95%CI** | [ 7.3 - 11.9 ] | [ 4.9 - 9.5 ] | [ 9.2 - 19.2 ] | [ 7.1 - 12.9 ] | [ 5.3 - 12.7 ] |
|  | **P 5** | 0.0 | 0.0 | 0.0 | 0.0 | 0.0 |
|  | **P 10** | 0.0 | 0.0 | 0.0 | 0.0 | 0.0 |
|  | **P 20** | 0.0 | 0.0 | 0.0 | 0.0 | 0.0 |
|  | **P 25** | 0.0 | 0.0 | 0.0 | 0.0 | 0.0 |
|  | **P 30** | 0.0 | 0.0 | 0.0 | 0.0 | 0.0 |
|  | **P 40** | 0.0 | 0.0 | 0.0 | 0.0 | 0.0 |
|  | **Median** | 0.0 | 0.0 | 0.0 | 0.0 | 0.0 |
|  | **P 60** | 0.0 | 0.0 | 0.0 | 0.0 | 0.0 |
|  | **P 70** | 0.0 | 0.0 | 0.0 | 0.0 | 0.0 |
|  | **P 75** | 0.0 | 0.0 | 33.3 | 0.0 | 0.0 |
|  | **P 80** | 33.3 | 0.0 | 33.3 | 33.3 | 0.0 |
|  | **P 90** | 33.3 | 33.3 | 66.7 | 33.3 | 33.3 |
|  | **P 95** | 66.7 | 33.3 | 66.7 | 53.3 | 66.7 |

* Deciles were not calculated for subgroups lower than 20 participants.

**Abbreviations. SD:** Standard Deviation; **95% CI:** 95% Confidence Interval; **P:** Percentile.

# Supplementary table 2.2.12. Reference norms at 2-year follow-up for the Insomnia of EORTC QLQ-C30.

|  |  | **EORTC QLQ-C30: Insomnia** | | | | |
| --- | --- | --- | --- | --- | --- | --- |
|  |  |  | **Charlson** | | **TNM stage** | |
|  |  | **All** | **0** | **≥ 1** | **0 – I** | **II - III** |
| **Age (years)**  **<40** | **n** | 35 | 33 | 2* | 14* | 21 |
|  | **Mean (SD)** | 25.7 (30.3) | 25.3 (31.2) | 33.3 (0.0) | 21.4 (31.0) | 28.6 (30.3) |
|  | **95%CI** | [ 15.7 - 35.8 ] | [ 14.6 - 35.9 ] | [ 33.3 - 33.3 ] | [ 5.2 - 37.6 ] | [ 15.6 - 41.5 ] |
|  | **P 5** | 0.0 | 0.0 |  |  | 0.0 |
|  | **P 10** | 0.0 | 0.0 |  |  | 0.0 |
|  | **P 20** | 0.0 | 0.0 |  |  | 0.0 |
|  | **P 25** | 0.0 | 0.0 |  |  | 0.0 |
|  | **P 30** | 0.0 | 0.0 |  |  | 0.0 |
|  | **P 40** | 0.0 | 0.0 |  |  | 0.0 |
|  | **Median** | 33.3 | 0.0 | 33.3 | 0.0 | 33.3 |
|  | **P 60** | 33.3 | 33.3 |  |  | 33.3 |
|  | **P 70** | 33.3 | 33.3 |  |  | 33.3 |
|  | **P 75** | 33.3 | 33.3 |  |  | 50.0 |
|  | **P 80** | 60.0 | 66.7 |  |  | 66.7 |
|  | **P 90** | 66.7 | 66.7 |  |  | 66.7 |
|  | **P 95** | 100.0 | 100.0 |  |  | 96.7 |
| **40-65** | **n** | 731 | 618 | 113 | 487 | 244 |
|  | **Mean (SD)** | 32.0 (32.8) | 31.0 (32.2) | 37.5 (35.7) | 30.4 (32.5) | 35.1 (33.4) |
|  | **95%CI** | [ 29.6 - 34.3 ] | [ 28.4 - 33.5 ] | [ 30.9 - 44.0 ] | [ 27.5 - 33.3 ] | [ 30.9 - 39.3 ] |
|  | **P 5** | 0.0 | 0.0 | 0.0 | 0.0 | 0.0 |
|  | **P 10** | 0.0 | 0.0 | 0.0 | 0.0 | 0.0 |
|  | **P 20** | 0.0 | 0.0 | 0.0 | 0.0 | 0.0 |
|  | **P 25** | 0.0 | 0.0 | 0.0 | 0.0 | 0.0 |
|  | **P 30** | 0.0 | 0.0 | 0.0 | 0.0 | 0.0 |
|  | **P 40** | 0.0 | 0.0 | 33.3 | 0.0 | 33.3 |
|  | **Median** | 33.3 | 33.3 | 33.3 | 33.3 | 33.3 |
|  | **P 60** | 33.3 | 33.3 | 33.3 | 33.3 | 33.3 |
|  | **P 70** | 33.3 | 33.3 | 66.7 | 33.3 | 66.7 |
|  | **P 75** | 66.7 | 66.7 | 66.7 | 33.3 | 66.7 |
|  | **P 80** | 66.7 | 66.7 | 66.7 | 66.7 | 66.7 |
|  | **P 90** | 100.0 | 66.7 | 100.0 | 66.7 | 100.0 |
|  | **P 95** | 100.0 | 100.0 | 100.0 | 100.0 | 100.0 |
| **>65** | **n** | 321 | 212 | 109 | 190 | 131 |
|  | **Mean (SD)** | 30.4 (31.5) | 28.8 (29.8) | 33.6 (34.4) | 30.9 (30.8) | 29.8 (32.6) |
|  | **95%CI** | [ 27.0 - 33.9 ] | [ 24.8 - 32.8 ] | [ 27.2 - 40.1 ] | [ 26.5 - 35.3 ] | [ 24.2 - 35.4 ] |
|  | **P 5** | 0.0 | 0.0 | 0.0 | 0.0 | 0.0 |
|  | **P 10** | 0.0 | 0.0 | 0.0 | 0.0 | 0.0 |
|  | **P 20** | 0.0 | 0.0 | 0.0 | 0.0 | 0.0 |
|  | **P 25** | 0.0 | 0.0 | 0.0 | 0.0 | 0.0 |
|  | **P 30** | 0.0 | 0.0 | 0.0 | 0.0 | 0.0 |
|  | **P 40** | 0.0 | 0.0 | 0.0 | 33.3 | 0.0 |
|  | **Median** | 33.3 | 33.3 | 33.3 | 33.3 | 33.3 |
|  | **P 60** | 33.3 | 33.3 | 33.3 | 33.3 | 33.3 |
|  | **P 70** | 33.3 | 33.3 | 33.3 | 33.3 | 33.3 |
|  | **P 75** | 33.3 | 33.3 | 66.7 | 33.3 | 33.3 |
|  | **P 80** | 66.7 | 46.7 | 66.7 | 66.7 | 66.7 |
|  | **P 90** | 66.7 | 66.7 | 100.0 | 66.7 | 100.0 |
|  | **P 95** | 100.0 | 100.0 | 100.0 | 100.0 | 100.0 |

* Deciles were not calculated for subgroups lower than 20 participants.

**Abbreviations. SD:** Standard Deviation; **95% CI:** 95% Confidence Interval; **P:** Percentile.

# Supplementary table 2.2.13. Reference norms at 2-year follow-up for the Appetite loss of EORTC QLQ-C30.

|  |  | **EORTC QLQ-C30: Appetite loss** | | | | |
| --- | --- | --- | --- | --- | --- | --- |
|  |  |  | **Charlson** | | **TNM stage** | |
|  |  | **All** | **0** | **≥ 1** | **0 – I** | **II - III** |
| **Age (years)**  **<40** | **n** | 35 | 33 | 2* | 14* | 21 |
|  | **Mean (SD)** | 2.9 (9.5) | 3.0 (9.7) | 0.0 (0.0) | 0.0 (0.0) | 4.8 (12.0) |
|  | **95%CI** | [ -0.3 - 6.0 ] | [ -0.3 - 6.4 ] | [ 0.0 - 0.0 ] | [ 0.0 - 0.0 ] | [ -0.4 - 9.9 ] |
|  | **P 5** | 0.0 | 0.0 |  |  | 0.0 |
|  | **P 10** | 0.0 | 0.0 |  |  | 0.0 |
|  | **P 20** | 0.0 | 0.0 |  |  | 0.0 |
|  | **P 25** | 0.0 | 0.0 |  |  | 0.0 |
|  | **P 30** | 0.0 | 0.0 |  |  | 0.0 |
|  | **P 40** | 0.0 | 0.0 |  |  | 0.0 |
|  | **Median** | 0.0 | 0.0 | 0.0 | 0.0 | 0.0 |
|  | **P 60** | 0.0 | 0.0 |  |  | 0.0 |
|  | **P 70** | 0.0 | 0.0 |  |  | 0.0 |
|  | **P 75** | 0.0 | 0.0 |  |  | 0.0 |
|  | **P 80** | 0.0 | 0.0 |  |  | 0.0 |
|  | **P 90** | 13.3 | 20.0 |  |  | 33.3 |
|  | **P 95** | 33.3 | 33.3 |  |  | 33.3 |
| **40-65** | **n** | 733 | 620 | 113 | 489 | 244 |
|  | **Mean (SD)** | 7.4 (18.3) | 6.8 (17.2) | 10.6 (23.3) | 5.8 (15.3) | 10.7 (22.9) |
|  | **95%CI** | [ 6.1 - 8.7 ] | [ 5.5 - 8.2 ] | [ 6.3 - 14.9 ] | [ 4.4 - 7.1 ] | [ 7.8 - 13.5 ] |
|  | **P 5** | 0.0 | 0.0 | 0.0 | 0.0 | 0.0 |
|  | **P 10** | 0.0 | 0.0 | 0.0 | 0.0 | 0.0 |
|  | **P 20** | 0.0 | 0.0 | 0.0 | 0.0 | 0.0 |
|  | **P 25** | 0.0 | 0.0 | 0.0 | 0.0 | 0.0 |
|  | **P 30** | 0.0 | 0.0 | 0.0 | 0.0 | 0.0 |
|  | **P 40** | 0.0 | 0.0 | 0.0 | 0.0 | 0.0 |
|  | **Median** | 0.0 | 0.0 | 0.0 | 0.0 | 0.0 |
|  | **P 60** | 0.0 | 0.0 | 0.0 | 0.0 | 0.0 |
|  | **P 70** | 0.0 | 0.0 | 0.0 | 0.0 | 0.0 |
|  | **P 75** | 0.0 | 0.0 | 0.0 | 0.0 | 0.0 |
|  | **P 80** | 0.0 | 0.0 | 33.3 | 0.0 | 33.3 |
|  | **P 90** | 33.3 | 33.3 | 33.3 | 33.3 | 33.3 |
|  | **P 95** | 33.3 | 33.3 | 66.7 | 33.3 | 66.7 |
| **>65** | **n** | 317 | 208 | 109 | 188 | 129 |
|  | **Mean (SD)** | 10.6 (23.3) | 7.5 (18.6) | 16.5 (29.6) | 10.1 (22.6) | 11.4 (24.5) |
|  | **95%CI** | [ 8.1 - 13.2 ] | [ 5.0 - 10.1 ] | [ 11.0 - 22.1 ] | [ 6.9 - 13.3 ] | [ 7.1 - 15.6 ] |
|  | **P 5** | 0.0 | 0.0 | 0.0 | 0.0 | 0.0 |
|  | **P 10** | 0.0 | 0.0 | 0.0 | 0.0 | 0.0 |
|  | **P 20** | 0.0 | 0.0 | 0.0 | 0.0 | 0.0 |
|  | **P 25** | 0.0 | 0.0 | 0.0 | 0.0 | 0.0 |
|  | **P 30** | 0.0 | 0.0 | 0.0 | 0.0 | 0.0 |
|  | **P 40** | 0.0 | 0.0 | 0.0 | 0.0 | 0.0 |
|  | **Median** | 0.0 | 0.0 | 0.0 | 0.0 | 0.0 |
|  | **P 60** | 0.0 | 0.0 | 0.0 | 0.0 | 0.0 |
|  | **P 70** | 0.0 | 0.0 | 0.0 | 0.0 | 0.0 |
|  | **P 75** | 0.0 | 0.0 | 33.3 | 0.0 | 0.0 |
|  | **P 80** | 33.3 | 0.0 | 33.3 | 33.3 | 33.3 |
|  | **P 90** | 33.3 | 33.3 | 66.7 | 33.3 | 33.3 |
|  | **P 95** | 66.7 | 33.3 | 100.0 | 66.7 | 66.7 |

* Deciles were not calculated for subgroups lower than 20 participants.

**Abbreviations. SD:** Standard Deviation; **95% CI:** 95% Confidence Interval; **P:** Percentile.

# Supplementary table 2.2.14. Reference norms at 2-year follow-up for the Constipation of EORTC QLQ-C30.

|  |  | **EORTC QLQ-C30: Constipation** | | | | |
| --- | --- | --- | --- | --- | --- | --- |
|  |  |  | **Charlson** | | **TNM stage** | |
|  |  | **All** | **0** | **≥ 1** | **0 – I** | **II - III** |
| **Age (years)**  **<40** | **n** | 35 | 33 | 2* | 14* | 21 |
|  | **Mean (SD)** | 13.3 (24.5) | 12.1 (23.3) | 33.3 (47.1) | 7.1 (19.3) | 17.5 (27.1) |
|  | **95%CI** | [ 5.2 - 21.5 ] | [ 4.2 - 20.1 ] | [ -32.0 - 98.7 ] | [ -3.0 - 17.3 ] | [ 5.9 - 29.1 ] |
|  | **P 5** | 0.0 | 0.0 |  |  | 0.0 |
|  | **P 10** | 0.0 | 0.0 |  |  | 0.0 |
|  | **P 20** | 0.0 | 0.0 |  |  | 0.0 |
|  | **P 25** | 0.0 | 0.0 |  |  | 0.0 |
|  | **P 30** | 0.0 | 0.0 |  |  | 0.0 |
|  | **P 40** | 0.0 | 0.0 |  |  | 0.0 |
|  | **Median** | 0.0 | 0.0 | 33.3 | 0.0 | 0.0 |
|  | **P 60** | 0.0 | 0.0 |  |  | 0.0 |
|  | **P 70** | 0.0 | 0.0 |  |  | 33.3 |
|  | **P 75** | 33.3 | 16.7 |  |  | 33.3 |
|  | **P 80** | 33.3 | 33.3 |  |  | 53.3 |
|  | **P 90** | 66.7 | 66.7 |  |  | 66.7 |
|  | **P 95** | 66.7 | 66.7 |  |  | 66.7 |
| **40-65** | **n** | 735 | 622 | 113 | 490 | 245 |
|  | **Mean (SD)** | 13.2 (23.9) | 12.6 (23.3) | 16.8 (26.8) | 13.0 (23.5) | 13.7 (24.6) |
|  | **95%CI** | [ 11.5 - 15.0 ] | [ 10.8 - 14.4 ] | [ 11.9 - 21.8 ] | [ 10.9 - 15.1 ] | [ 10.7 - 16.8 ] |
|  | **P 5** | 0.0 | 0.0 | 0.0 | 0.0 | 0.0 |
|  | **P 10** | 0.0 | 0.0 | 0.0 | 0.0 | 0.0 |
|  | **P 20** | 0.0 | 0.0 | 0.0 | 0.0 | 0.0 |
|  | **P 25** | 0.0 | 0.0 | 0.0 | 0.0 | 0.0 |
|  | **P 30** | 0.0 | 0.0 | 0.0 | 0.0 | 0.0 |
|  | **P 40** | 0.0 | 0.0 | 0.0 | 0.0 | 0.0 |
|  | **Median** | 0.0 | 0.0 | 0.0 | 0.0 | 0.0 |
|  | **P 60** | 0.0 | 0.0 | 0.0 | 0.0 | 0.0 |
|  | **P 70** | 0.0 | 0.0 | 33.3 | 0.0 | 0.0 |
|  | **P 75** | 33.3 | 33.3 | 33.3 | 33.3 | 33.3 |
|  | **P 80** | 33.3 | 33.3 | 33.3 | 33.3 | 33.3 |
|  | **P 90** | 33.3 | 33.3 | 66.7 | 33.3 | 66.7 |
|  | **P 95** | 66.7 | 66.7 | 66.7 | 66.7 | 66.7 |
| **>65** | **n** | 319 | 211 | 108 | 186 | 133 |
|  | **Mean (SD)** | 17.0 (26.4) | 16.1 (25.7) | 18.8 (27.8) | 17.9 (26.6) | 15.8 (26.1) |
|  | **95%CI** | [ 14.1 - 19.9 ] | [ 12.6 - 19.6 ] | [ 13.6 - 24.1 ] | [ 14.1 - 21.8 ] | [ 11.3 - 20.2 ] |
|  | **P 5** | 0.0 | 0.0 | 0.0 | 0.0 | 0.0 |
|  | **P 10** | 0.0 | 0.0 | 0.0 | 0.0 | 0.0 |
|  | **P 20** | 0.0 | 0.0 | 0.0 | 0.0 | 0.0 |
|  | **P 25** | 0.0 | 0.0 | 0.0 | 0.0 | 0.0 |
|  | **P 30** | 0.0 | 0.0 | 0.0 | 0.0 | 0.0 |
|  | **P 40** | 0.0 | 0.0 | 0.0 | 0.0 | 0.0 |
|  | **Median** | 0.0 | 0.0 | 0.0 | 0.0 | 0.0 |
|  | **P 60** | 0.0 | 0.0 | 0.0 | 0.0 | 0.0 |
|  | **P 70** | 33.3 | 33.3 | 33.3 | 33.3 | 33.3 |
|  | **P 75** | 33.3 | 33.3 | 33.3 | 33.3 | 33.3 |
|  | **P 80** | 33.3 | 33.3 | 33.3 | 33.3 | 33.3 |
|  | **P 90** | 66.7 | 66.7 | 66.7 | 66.7 | 66.7 |
|  | **P 95** | 66.7 | 66.7 | 66.7 | 66.7 | 66.7 |

* Deciles were not calculated for subgroups lower than 20 participants.

**Abbreviations. SD:** Standard Deviation; **95% CI:** 95% Confidence Interval; **P:** Percentile.

# Supplementary table 2.2.15. Reference norms at 2-year follow-up for the Diarrhea of EORTC QLQ-C30.

|  |  | **EORTC QLQ-C30: Diarrhea** | | | | |
| --- | --- | --- | --- | --- | --- | --- |
|  |  |  | **Charlson** | | **TNM stage** | |
|  |  | **All** | **0** | **≥ 1** | **0 – I** | **II - III** |
| **Age (years)**  **<40** | **n** | 35 | 33 | 2* | 14* | 21 |
|  | **Mean (SD)** | 4.8 (14.3) | 4.0 (13.8) | 16.7 (23.6) | 2.4 (8.9) | 6.3 (17.1) |
|  | **95%CI** | [ 0.0 - 9.5 ] | [ -0.7 - 8.8 ] | [ -16.0 - 49.3 ] | [ -2.3 - 7.0 ] | [ -0.9 - 13.6 ] |
|  | **P 5** | 0.0 | 0.0 |  |  | 0.0 |
|  | **P 10** | 0.0 | 0.0 |  |  | 0.0 |
|  | **P 20** | 0.0 | 0.0 |  |  | 0.0 |
|  | **P 25** | 0.0 | 0.0 |  |  | 0.0 |
|  | **P 30** | 0.0 | 0.0 |  |  | 0.0 |
|  | **P 40** | 0.0 | 0.0 |  |  | 0.0 |
|  | **Median** | 0.0 | 0.0 | 16.7 | 0.0 | 0.0 |
|  | **P 60** | 0.0 | 0.0 |  |  | 0.0 |
|  | **P 70** | 0.0 | 0.0 |  |  | 0.0 |
|  | **P 75** | 0.0 | 0.0 |  |  | 0.0 |
|  | **P 80** | 0.0 | 0.0 |  |  | 0.0 |
|  | **P 90** | 33.3 | 20.0 |  |  | 33.3 |
|  | **P 95** | 40.0 | 43.3 |  |  | 63.3 |
| **40-65** | **n** | 730 | 617 | 113 | 487 | 243 |
|  | **Mean (SD)** | 4.9 (14.4) | 4.5 (13.1) | 7.1 (19.6) | 4.7 (14.1) | 5.3 (15.0) |
|  | **95%CI** | [ 3.8 - 5.9 ] | [ 3.4 - 5.5 ] | [ 3.5 - 10.7 ] | [ 3.4 - 5.9 ] | [ 3.5 - 7.2 ] |
|  | **P 5** | 0.0 | 0.0 | 0.0 | 0.0 | 0.0 |
|  | **P 10** | 0.0 | 0.0 | 0.0 | 0.0 | 0.0 |
|  | **P 20** | 0.0 | 0.0 | 0.0 | 0.0 | 0.0 |
|  | **P 25** | 0.0 | 0.0 | 0.0 | 0.0 | 0.0 |
|  | **P 30** | 0.0 | 0.0 | 0.0 | 0.0 | 0.0 |
|  | **P 40** | 0.0 | 0.0 | 0.0 | 0.0 | 0.0 |
|  | **Median** | 0.0 | 0.0 | 0.0 | 0.0 | 0.0 |
|  | **P 60** | 0.0 | 0.0 | 0.0 | 0.0 | 0.0 |
|  | **P 70** | 0.0 | 0.0 | 0.0 | 0.0 | 0.0 |
|  | **P 75** | 0.0 | 0.0 | 0.0 | 0.0 | 0.0 |
|  | **P 80** | 0.0 | 0.0 | 0.0 | 0.0 | 0.0 |
|  | **P 90** | 33.3 | 33.3 | 33.3 | 33.3 | 33.3 |
|  | **P 95** | 33.3 | 33.3 | 66.7 | 33.3 | 33.3 |
| **>65** | **n** | 318 | 210 | 108 | 186 | 132 |
|  | **Mean (SD)** | 8.6 (19.9) | 7.0 (17.4) | 11.7 (23.8) | 7.3 (17.7) | 10.4 (22.6) |
|  | **95%CI** | [ 6.4 - 10.8 ] | [ 4.6 - 9.3 ] | [ 7.2 - 16.2 ] | [ 4.8 - 9.9 ] | [ 6.5 - 14.2 ] |
|  | **P 5** | 0.0 | 0.0 | 0.0 | 0.0 | 0.0 |
|  | **P 10** | 0.0 | 0.0 | 0.0 | 0.0 | 0.0 |
|  | **P 20** | 0.0 | 0.0 | 0.0 | 0.0 | 0.0 |
|  | **P 25** | 0.0 | 0.0 | 0.0 | 0.0 | 0.0 |
|  | **P 30** | 0.0 | 0.0 | 0.0 | 0.0 | 0.0 |
|  | **P 40** | 0.0 | 0.0 | 0.0 | 0.0 | 0.0 |
|  | **Median** | 0.0 | 0.0 | 0.0 | 0.0 | 0.0 |
|  | **P 60** | 0.0 | 0.0 | 0.0 | 0.0 | 0.0 |
|  | **P 70** | 0.0 | 0.0 | 0.0 | 0.0 | 0.0 |
|  | **P 75** | 0.0 | 0.0 | 0.0 | 0.0 | 0.0 |
|  | **P 80** | 0.0 | 0.0 | 33.3 | 0.0 | 33.3 |
|  | **P 90** | 33.3 | 33.3 | 66.7 | 33.3 | 33.3 |
|  | **P 95** | 66.7 | 33.3 | 66.7 | 33.3 | 66.7 |

* Deciles were not calculated for subgroups lower than 20 participants.

**Abbreviations. SD:** Standard Deviation; **95% CI:** 95% Confidence Interval; **P:** Percentile.

# Supplementary table 2.2.16. Reference norms at 2-year follow-up for the Financial difficulties of EORTC QLQ-C30.

|  |  | **EORTC QLQ-C30: Financial difficulties** | | | | |
| --- | --- | --- | --- | --- | --- | --- |
|  |  |  | **Charlson** | | **TNM stage** | |
|  |  | **All** | **0** | **≥ 1** | **0 – I** | **II - III** |
| **Age (years)**  **<40** | **n** | 35 | 33 | 2* | 14* | 21 |
|  | **Mean (SD)** | 17.1 (29.6) | 18.2 (30.2) | 0.0 (0.0) | 4.8 (12.1) | 25.4 (34.8) |
|  | **95%CI** | [ 7.3 - 26.9 ] | [ 7.9 - 28.5 ] | [ 0.0 - 0.0 ] | [ -1.6 - 11.1 ] | [ 10.5 - 40.3 ] |
|  | **P 5** | 0.0 | 0.0 |  |  | 0.0 |
|  | **P 10** | 0.0 | 0.0 |  |  | 0.0 |
|  | **P 20** | 0.0 | 0.0 |  |  | 0.0 |
|  | **P 25** | 0.0 | 0.0 |  |  | 0.0 |
|  | **P 30** | 0.0 | 0.0 |  |  | 0.0 |
|  | **P 40** | 0.0 | 0.0 |  |  | 0.0 |
|  | **Median** | 0.0 | 0.0 | 0.0 | 0.0 | 0.0 |
|  | **P 60** | 0.0 | 0.0 |  |  | 6.7 |
|  | **P 70** | 6.7 | 26.7 |  |  | 66.7 |
|  | **P 75** | 33.3 | 33.3 |  |  | 66.7 |
|  | **P 80** | 60.0 | 66.7 |  |  | 66.7 |
|  | **P 90** | 66.7 | 66.7 |  |  | 66.7 |
|  | **P 95** | 73.3 | 76.7 |  |  | 96.7 |
| **40-65** | **n** | 731 | 618 | 113 | 489 | 242 |
|  | **Mean (SD)** | 13.1 (25.1) | 13.0 (25.1) | 13.6 (25.1) | 11.5 (23.8) | 16.3 (27.4) |
|  | **95%CI** | [ 11.3 - 14.9 ] | [ 11.0 - 15.0 ] | [ 8.9 - 18.2 ] | [ 9.4 - 13.6 ] | [ 12.8 - 19.7 ] |
|  | **P 5** | 0.0 | 0.0 | 0.0 | 0.0 | 0.0 |
|  | **P 10** | 0.0 | 0.0 | 0.0 | 0.0 | 0.0 |
|  | **P 20** | 0.0 | 0.0 | 0.0 | 0.0 | 0.0 |
|  | **P 25** | 0.0 | 0.0 | 0.0 | 0.0 | 0.0 |
|  | **P 30** | 0.0 | 0.0 | 0.0 | 0.0 | 0.0 |
|  | **P 40** | 0.0 | 0.0 | 0.0 | 0.0 | 0.0 |
|  | **Median** | 0.0 | 0.0 | 0.0 | 0.0 | 0.0 |
|  | **P 60** | 0.0 | 0.0 | 0.0 | 0.0 | 0.0 |
|  | **P 70** | 0.0 | 0.0 | 0.0 | 0.0 | 33.3 |
|  | **P 75** | 33.3 | 33.3 | 33.3 | 0.0 | 33.3 |
|  | **P 80** | 33.3 | 33.3 | 33.3 | 33.3 | 33.3 |
|  | **P 90** | 33.3 | 33.3 | 66.7 | 33.3 | 66.7 |
|  | **P 95** | 66.7 | 66.7 | 66.7 | 66.7 | 66.7 |
| **>65** | **n** | 324 | 215 | 109 | 191 | 133 |
|  | **Mean (SD)** | 7.2 (19.0) | 5.6 (15.4) | 10.4 (24.3) | 7.7 (20.8) | 6.5 (16.1) |
|  | **95%CI** | [ 5.1 - 9.3 ] | [ 3.5 - 7.6 ] | [ 5.8 - 15.0 ] | [ 4.7 - 10.6 ] | [ 3.8 - 9.3 ] |
|  | **P 5** | 0.0 | 0.0 | 0.0 | 0.0 | 0.0 |
|  | **P 10** | 0.0 | 0.0 | 0.0 | 0.0 | 0.0 |
|  | **P 20** | 0.0 | 0.0 | 0.0 | 0.0 | 0.0 |
|  | **P 25** | 0.0 | 0.0 | 0.0 | 0.0 | 0.0 |
|  | **P 30** | 0.0 | 0.0 | 0.0 | 0.0 | 0.0 |
|  | **P 40** | 0.0 | 0.0 | 0.0 | 0.0 | 0.0 |
|  | **Median** | 0.0 | 0.0 | 0.0 | 0.0 | 0.0 |
|  | **P 60** | 0.0 | 0.0 | 0.0 | 0.0 | 0.0 |
|  | **P 70** | 0.0 | 0.0 | 0.0 | 0.0 | 0.0 |
|  | **P 75** | 0.0 | 0.0 | 0.0 | 0.0 | 0.0 |
|  | **P 80** | 0.0 | 0.0 | 0.0 | 0.0 | 0.0 |
|  | **P 90** | 33.3 | 33.3 | 33.3 | 33.3 | 33.3 |
|  | **P 95** | 33.3 | 33.3 | 66.7 | 66.7 | 33.3 |

* Deciles were not calculated for subgroups lower than 20 participants.

**Abbreviations. SD:** Standard Deviation; **95% CI:** 95% Confidence Interval; **P:** Percentile.

# Supplementary table 2.3.1. Reference norms at 2-year follow-up for the Body Image Scale of EORTC QLQ-BR23.

|  |  | **EORTC QLQ-BR23: Body Image Scale** | | | | |
| --- | --- | --- | --- | --- | --- | --- |
|  |  |  | **Charlson** | | **TNM stage** | |
|  |  | **All** | **0** | **≥ 1** | **0 – I** | **II - III** |
| **Age (years)**  **<40** | **n** | 33 | 31 | 2* | 14* | 19* |
|  | **Mean (SD)** | 79.8 (29.0) | 78.5 (29.5) | 100.0 (0.0) | 82.7 (29.5) | 77.6 (29.3) |
|  | **95%CI** | [ 69.9 - 89.7 ] | [ 68.1 - 88.9 ] | [ 100.0 - 100.0 ] | [ 67.3 - 98.2 ] | [ 64.5 - 90.8 ] |
|  | **P 5** | 5.8 | 5.0 |  |  |  |
|  | **P 10** | 36.7 | 35.0 |  |  |  |
|  | **P 20** | 48.3 | 45.0 |  |  |  |
|  | **P 25** | 62.5 | 58.3 |  |  |  |
|  | **P 30** | 75.0 | 71.7 |  |  |  |
|  | **P 40** | 88.3 | 83.3 |  |  |  |
|  | **Median** | 100.0 | 91.7 | 100.0 | 100.0 | 91.7 |
|  | **P 60** | 100.0 | 100.0 |  |  |  |
|  | **P 70** | 100.0 | 100.0 |  |  |  |
|  | **P 75** | 100.0 | 100.0 |  |  |  |
|  | **P 80** | 100.0 | 100.0 |  |  |  |
|  | **P 90** | 100.0 | 100.0 |  |  |  |
|  | **P 95** | 100.0 | 100.0 |  |  |  |
| **40-65** | **n** | 738 | 624 | 114 | 493 | 245 |
|  | **Mean (SD)** | 82.7 (25.9) | 82.8 (25.6) | 81.8 (27.7) | 85.6 (23.2) | 76.7 (29.9) |
|  | **95%CI** | [ 80.8 - 84.5 ] | [ 80.8 - 84.8 ] | [ 76.7 - 86.9 ] | [ 83.6 - 87.7 ] | [ 73.0 - 80.4 ] |
|  | **P 5** | 16.7 | 16.7 | 16.7 | 33.3 | 8.3 |
|  | **P 10** | 41.7 | 45.8 | 33.3 | 53.3 | 25.0 |
|  | **P 20** | 66.7 | 66.7 | 66.7 | 66.7 | 50.0 |
|  | **P 25** | 75.0 | 75.0 | 72.9 | 75.0 | 66.7 |
|  | **P 30** | 75.0 | 75.0 | 75.0 | 83.3 | 75.0 |
|  | **P 40** | 91.7 | 91.7 | 91.7 | 91.7 | 75.0 |
|  | **Median** | 100.0 | 100.0 | 100.0 | 100.0 | 91.7 |
|  | **P 60** | 100.0 | 100.0 | 100.0 | 100.0 | 100.0 |
|  | **P 70** | 100.0 | 100.0 | 100.0 | 100.0 | 100.0 |
|  | **P 75** | 100.0 | 100.0 | 100.0 | 100.0 | 100.0 |
|  | **P 80** | 100.0 | 100.0 | 100.0 | 100.0 | 100.0 |
|  | **P 90** | 100.0 | 100.0 | 100.0 | 100.0 | 100.0 |
|  | **P 95** | 100.0 | 100.0 | 100.0 | 100.0 | 100.0 |
| **>65** | **n** | 319 | 215 | 104 | 189 | 130 |
|  | **Mean (SD)** | 89.1 (20.9) | 89.9 (21.1) | 87.3 (20.5) | 91.3 (16.7) | 85.8 (25.5) |
|  | **95%CI** | [ 86.8 - 91.4 ] | [ 87.1 - 92.7 ] | [ 83.4 - 91.3 ] | [ 88.9 - 93.7 ] | [ 81.5 - 90.2 ] |
|  | **P 5** | 41.7 | 33.3 | 41.7 | 50.0 | 21.3 |
|  | **P 10** | 58.3 | 66.7 | 58.3 | 66.7 | 41.7 |
|  | **P 20** | 83.3 | 83.3 | 75.0 | 83.3 | 75.0 |
|  | **P 25** | 83.3 | 91.7 | 83.3 | 91.7 | 83.3 |
|  | **P 30** | 91.7 | 91.7 | 83.3 | 91.7 | 85.8 |
|  | **P 40** | 100.0 | 100.0 | 91.7 | 100.0 | 100.0 |
|  | **Median** | 100.0 | 100.0 | 100.0 | 100.0 | 100.0 |
|  | **P 60** | 100.0 | 100.0 | 100.0 | 100.0 | 100.0 |
|  | **P 70** | 100.0 | 100.0 | 100.0 | 100.0 | 100.0 |
|  | **P 75** | 100.0 | 100.0 | 100.0 | 100.0 | 100.0 |
|  | **P 80** | 100.0 | 100.0 | 100.0 | 100.0 | 100.0 |
|  | **P 90** | 100.0 | 100.0 | 100.0 | 100.0 | 100.0 |
|  | **P 95** | 100.0 | 100.0 | 100.0 | 100.0 | 100.0 |

* Deciles were not calculated for subgroups lower than 20 participants.

**Abbreviations. SD:** Standard Deviation; **95% CI:** 95% Confidence Interval; **P:** Percentile.

# Supplementary table 2.3.2. Reference norms at 2-year follow-up for the Sexual Function Scale of EORTC QLQ-BR23.

|  |  | **EORTC QLQ-BR23: Sexual Function Scale** | | | | |
| --- | --- | --- | --- | --- | --- | --- |
|  |  |  | **Charlson** | | **TNM stage** | |
|  |  | **All** | **0** | **≥ 1** | **0 – I** | **II - III** |
| **Age (years)**  **<40** | **n** | 34 | 32 | 2* | 14* | 20 |
|  | **Mean (SD)** | 30.4 (20.7) | 29.2 (20.3) | 50.0 (23.6) | 39.3 (23.2) | 24.2 (16.6) |
|  | **95%CI** | [ 23.4 - 37.4 ] | [ 22.1 - 36.2 ] | [ 17.3 - 82.7 ] | [ 27.1 - 51.4 ] | [ 16.9 - 31.5 ] |
|  | **P 5** | 0.0 | 0.0 |  |  | 0.0 |
|  | **P 10** | 0.0 | 0.0 |  |  | 0.0 |
|  | **P 20** | 0.0 | 0.0 |  |  | 0.0 |
|  | **P 25** | 16.7 | 16.7 |  |  | 4.2 |
|  | **P 30** | 16.7 | 16.7 |  |  | 16.7 |
|  | **P 40** | 33.3 | 33.3 |  |  | 23.3 |
|  | **Median** | 33.3 | 33.3 | 50.0 | 33.3 | 33.3 |
|  | **P 60** | 33.3 | 33.3 |  |  | 33.3 |
|  | **P 70** | 33.3 | 33.3 |  |  | 33.3 |
|  | **P 75** | 37.5 | 33.3 |  |  | 33.3 |
|  | **P 80** | 50.0 | 50.0 |  |  | 33.3 |
|  | **P 90** | 66.7 | 61.7 |  |  | 48.3 |
|  | **P 95** | 66.7 | 66.7 |  |  | 50.0 |
| **40-65** | **n** | 723 | 610 | 113 | 484 | 239 |
|  | **Mean (SD)** | 25.1 (24.2) | 26.1 (24.4) | 19.5 (22.6) | 25.9 (24.6) | 23.4 (23.3) |
|  | **95%CI** | [ 23.3 - 26.9 ] | [ 24.2 - 28.1 ] | [ 15.3 - 23.6 ] | [ 23.7 - 28.1 ] | [ 20.5 - 26.4 ] |
|  | **P 5** | 0.0 | 0.0 | 0.0 | 0.0 | 0.0 |
|  | **P 10** | 0.0 | 0.0 | 0.0 | 0.0 | 0.0 |
|  | **P 20** | 0.0 | 0.0 | 0.0 | 0.0 | 0.0 |
|  | **P 25** | 0.0 | 0.0 | 0.0 | 0.0 | 0.0 |
|  | **P 30** | 0.0 | 0.0 | 0.0 | 0.0 | 0.0 |
|  | **P 40** | 16.7 | 16.7 | 0.0 | 16.7 | 16.7 |
|  | **Median** | 33.3 | 33.3 | 16.7 | 33.3 | 16.7 |
|  | **P 60** | 33.3 | 33.3 | 33.3 | 33.3 | 33.3 |
|  | **P 70** | 33.3 | 33.3 | 33.3 | 33.3 | 33.3 |
|  | **P 75** | 33.3 | 33.3 | 33.3 | 33.3 | 33.3 |
|  | **P 80** | 33.3 | 33.3 | 33.3 | 33.3 | 33.3 |
|  | **P 90** | 66.7 | 66.7 | 50.0 | 66.7 | 66.7 |
|  | **P 95** | 66.7 | 66.7 | 66.7 | 66.7 | 66.7 |
| **>65** | **n** | 301 | 199 | 102 | 179 | 122 |
|  | **Mean (SD)** | 9.9 (18.1) | 10.1 (18.6) | 9.6 (17.1) | 10.7 (19.1) | 8.7 (16.5) |
|  | **95%CI** | [ 7.9 - 12.0 ] | [ 7.5 - 12.6 ] | [ 6.3 - 13.0 ] | [ 7.9 - 13.5 ] | [ 5.8 - 11.7 ] |
|  | **P 5** | 0.0 | 0.0 | 0.0 | 0.0 | 0.0 |
|  | **P 10** | 0.0 | 0.0 | 0.0 | 0.0 | 0.0 |
|  | **P 20** | 0.0 | 0.0 | 0.0 | 0.0 | 0.0 |
|  | **P 25** | 0.0 | 0.0 | 0.0 | 0.0 | 0.0 |
|  | **P 30** | 0.0 | 0.0 | 0.0 | 0.0 | 0.0 |
|  | **P 40** | 0.0 | 0.0 | 0.0 | 0.0 | 0.0 |
|  | **Median** | 0.0 | 0.0 | 0.0 | 0.0 | 0.0 |
|  | **P 60** | 0.0 | 0.0 | 0.0 | 0.0 | 0.0 |
|  | **P 70** | 0.0 | 0.0 | 1.7 | 16.7 | 0.0 |
|  | **P 75** | 16.7 | 16.7 | 16.7 | 16.7 | 16.7 |
|  | **P 80** | 33.3 | 33.3 | 23.3 | 33.3 | 16.7 |
|  | **P 90** | 33.3 | 33.3 | 33.3 | 33.3 | 33.3 |
|  | **P 95** | 50.0 | 50.0 | 50.0 | 50.0 | 47.5 |

* Deciles were not calculated for subgroups lower than 20 participants.

**Abbreviations. SD:** Standard Deviation; **95% CI:** 95% Confidence Interval; **P:** Percentile.

# Supplementary table 2.3.3. Reference norms for the Sexual enjoyment Scale of EORTC QLQ-BR23.

|  |  | **EORTC QLQ-BR23: Sexual Enjoyment Scale** | | | | |
| --- | --- | --- | --- | --- | --- | --- |
|  |  |  | **Charlson** | | **TNM stage** | |
|  |  | **All** | **0** | **≥ 1** | **0 – I** | **II - III** |
| **Age (years)**  **<40** | **n** | 26 | 24 | 2* | 12* | 14* |
|  | **Mean (SD)** | 62.8 (23.7) | 62.5 (24.7) | 66.7 (0.0) | 72.2 (23.9) | 54.8 (21.1) |
|  | **95%CI** | [ 53.7 - 71.9 ] | [ 52.6 - 72.4 ] | [ 66.7 - 66.7 ] | [ 58.7 - 85.8 ] | [ 43.7 - 65.8 ] |
|  | **P 5** | 33.3 | 33.3 |  |  |  |
|  | **P 10** | 33.3 | 33.3 |  |  |  |
|  | **P 20** | 33.3 | 33.3 |  |  |  |
|  | **P 25** | 33.3 | 33.3 |  |  |  |
|  | **P 30** | 36.7 | 33.3 |  |  |  |
|  | **P 40** | 66.7 | 66.7 |  |  |  |
|  | **Median** | 66.7 | 66.7 | 66.7 | 66.7 | 66.7 |
|  | **P 60** | 66.7 | 66.7 |  |  |  |
|  | **P 70** | 66.7 | 66.7 |  |  |  |
|  | **P 75** | 66.7 | 66.7 |  |  |  |
|  | **P 80** | 86.7 | 100.0 |  |  |  |
|  | **P 90** | 100.0 | 100.0 |  |  |  |
|  | **P 95** | 100.0 | 100.0 |  |  |  |
| **40-65** | **n** | 417 | 362 | 55 | 284 | 133 |
|  | **Mean (SD)** | 53.1 (27.1) | 53.5 (27.2) | 50.3 (26.4) | 54.2 (27.8) | 50.6 (25.5) |
|  | **95%CI** | [ 50.5 - 55.7 ] | [ 50.7 - 56.3 ] | [ 43.3 - 57.3 ] | [ 51.0 - 57.5 ] | [ 46.3 - 55.0 ] |
|  | **P 5** | 0.0 | 0.0 | 0.0 | 0.0 | 0.0 |
|  | **P 10** | 33.3 | 33.3 | 0.0 | 33.3 | 33.3 |
|  | **P 20** | 33.3 | 33.3 | 33.3 | 33.3 | 33.3 |
|  | **P 25** | 33.3 | 33.3 | 33.3 | 33.3 | 33.3 |
|  | **P 30** | 33.3 | 33.3 | 33.3 | 33.3 | 33.3 |
|  | **P 40** | 33.3 | 33.3 | 33.3 | 33.3 | 33.3 |
|  | **Median** | 66.7 | 66.7 | 66.7 | 66.7 | 33.3 |
|  | **P 60** | 66.7 | 66.7 | 66.7 | 66.7 | 66.7 |
|  | **P 70** | 66.7 | 66.7 | 66.7 | 66.7 | 66.7 |
|  | **P 75** | 66.7 | 66.7 | 66.7 | 66.7 | 66.7 |
|  | **P 80** | 66.7 | 66.7 | 66.7 | 66.7 | 66.7 |
|  | **P 90** | 100.0 | 100.0 | 66.7 | 100.0 | 86.7 |
|  | **P 95** | 100.0 | 100.0 | 100.0 | 100.0 | 100.0 |
| **>65** | **n** | 93 | 63 | 30 | 57 | 36 |
|  | **Mean (SD)** | 34.8 (27.3) | 33.9 (28.4) | 36.7 (25.3) | 34.5 (27.4) | 35.2 (27.5) |
|  | **95%CI** | [ 29.2 - 40.3 ] | [ 26.9 - 40.9 ] | [ 27.6 - 45.7 ] | [ 27.4 - 41.6 ] | [ 26.2 - 44.2 ] |
|  | **P 5** | 0.0 | 0.0 | 0.0 | 0.0 | 0.0 |
|  | **P 10** | 0.0 | 0.0 | 0.0 | 0.0 | 0.0 |
|  | **P 20** | 0.0 | 0.0 | 0.0 | 0.0 | 0.0 |
|  | **P 25** | 0.0 | 0.0 | 25.0 | 0.0 | 0.0 |
|  | **P 30** | 33.3 | 0.0 | 33.3 | 13.3 | 33.3 |
|  | **P 40** | 33.3 | 33.3 | 33.3 | 33.3 | 33.3 |
|  | **Median** | 33.3 | 33.3 | 33.3 | 33.3 | 33.3 |
|  | **P 60** | 33.3 | 33.3 | 33.3 | 33.3 | 33.3 |
|  | **P 70** | 66.7 | 60.0 | 66.7 | 66.7 | 63.3 |
|  | **P 75** | 66.7 | 66.7 | 66.7 | 66.7 | 66.7 |
|  | **P 80** | 66.7 | 66.7 | 66.7 | 66.7 | 66.7 |
|  | **P 90** | 66.7 | 66.7 | 66.7 | 66.7 | 66.7 |
|  | **P 95** | 66.7 | 66.7 | 66.7 | 66.7 | 71.7 |

* Deciles were not calculated for subgroups lower than 20 participants.

**Abbreviations. SD:** Standard Deviation; **95% CI:** 95% Confidence Interval; **P:** Percentile.

# Supplementary table 2.3.4. Reference norms at 2-year follow-up for the Future perspective Scale of EORTC QLQ-BR23.

|  |  | **EORTC QLQ-BR23: Future perspective Scale** | | | | |
| --- | --- | --- | --- | --- | --- | --- |
|  |  |  | **Charlson** | | **TNM stage** | |
|  |  | **All** | **0** | **≥ 1** | **0 – I** | **II - III** |
| **Age (years)**  **<40** | **n** | 33 | 31 | 2* | 14* | 19* |
|  | **Mean (SD)** | 56.6 (37.7) | 55.9 (37.9) | 66.7 (47.1) | 66.7 (32.0) | 49.1 (40.6) |
|  | **95%CI** | [ 43.7 - 69.4 ] | [ 42.6 - 69.3 ] | [ 1.3 - 132.0 ] | [ 49.9 - 83.4 ] | [ 30.9 - 67.4 ] |
|  | **P 5** | 0.0 | 0.0 |  |  |  |
|  | **P 10** | 0.0 | 0.0 |  |  |  |
|  | **P 20** | 26.7 | 13.3 |  |  |  |
|  | **P 25** | 33.3 | 33.3 |  |  |  |
|  | **P 30** | 33.3 | 33.3 |  |  |  |
|  | **P 40** | 33.3 | 33.3 |  |  |  |
|  | **Median** | 66.7 | 66.7 | 66.7 | 66.7 | 33.3 |
|  | **P 60** | 66.7 | 66.7 |  |  |  |
|  | **P 70** | 100.0 | 100.0 |  |  |  |
|  | **P 75** | 100.0 | 100.0 |  |  |  |
|  | **P 80** | 100.0 | 100.0 |  |  |  |
|  | **P 90** | 100.0 | 100.0 |  |  |  |
|  | **P 95** | 100.0 | 100.0 |  |  |  |
| **40-65** | **n** | 738 | 624 | 114 | 493 | 245 |
|  | **Mean (SD)** | 57.0 (31.8) | 57.5 (31.4) | 54.4 (33.8) | 57.9 (31.7) | 55.1 (32.0) |
|  | **95%CI** | [ 54.7 - 59.3 ] | [ 55.0 - 59.9 ] | [ 48.2 - 60.6 ] | [ 55.1 - 60.7 ] | [ 51.1 - 59.1 ] |
|  | **P 5** | 0.0 | 0.0 | 0.0 | 0.0 | 0.0 |
|  | **P 10** | 0.0 | 0.0 | 0.0 | 0.0 | 0.0 |
|  | **P 20** | 33.3 | 33.3 | 33.3 | 33.3 | 33.3 |
|  | **P 25** | 33.3 | 33.3 | 33.3 | 33.3 | 33.3 |
|  | **P 30** | 33.3 | 33.3 | 33.3 | 33.3 | 33.3 |
|  | **P 40** | 66.7 | 66.7 | 66.7 | 66.7 | 66.7 |
|  | **Median** | 66.7 | 66.7 | 66.7 | 66.7 | 66.7 |
|  | **P 60** | 66.7 | 66.7 | 66.7 | 66.7 | 66.7 |
|  | **P 70** | 66.7 | 66.7 | 66.7 | 66.7 | 66.7 |
|  | **P 75** | 66.7 | 66.7 | 66.7 | 66.7 | 66.7 |
|  | **P 80** | 100.0 | 100.0 | 100.0 | 100.0 | 66.7 |
|  | **P 90** | 100.0 | 100.0 | 100.0 | 100.0 | 100.0 |
|  | **P 95** | 100.0 | 100.0 | 100.0 | 100.0 | 100.0 |
| **>65** | **n** | 321 | 216 | 105 | 189 | 132 |
|  | **Mean (SD)** | 65.2 (31.5) | 64.7 (31.5) | 66.3 (31.5) | 68.4 (29.3) | 60.6 (33.9) |
|  | **95%CI** | [ 61.8 - 68.7 ] | [ 60.5 - 68.9 ] | [ 60.3 - 72.4 ] | [ 64.2 - 72.6 ] | [ 54.8 - 66.4 ] |
|  | **P 5** | 0.0 | 0.0 | 0.0 | 0.0 | 0.0 |
|  | **P 10** | 6.7 | 0.0 | 20.0 | 33.3 | 0.0 |
|  | **P 20** | 33.3 | 33.3 | 33.3 | 33.3 | 33.3 |
|  | **P 25** | 33.3 | 33.3 | 33.3 | 66.7 | 33.3 |
|  | **P 30** | 66.7 | 66.7 | 66.7 | 66.7 | 33.3 |
|  | **P 40** | 66.7 | 66.7 | 66.7 | 66.7 | 66.7 |
|  | **Median** | 66.7 | 66.7 | 66.7 | 66.7 | 66.7 |
|  | **P 60** | 66.7 | 66.7 | 66.7 | 66.7 | 66.7 |
|  | **P 70** | 100.0 | 100.0 | 100.0 | 100.0 | 66.7 |
|  | **P 75** | 100.0 | 100.0 | 100.0 | 100.0 | 100.0 |
|  | **P 80** | 100.0 | 100.0 | 100.0 | 100.0 | 100.0 |
|  | **P 90** | 100.0 | 100.0 | 100.0 | 100.0 | 100.0 |
|  | **P 95** | 100.0 | 100.0 | 100.0 | 100.0 | 100.0 |

* Deciles were not calculated for subgroups lower than 20 participants.

**Abbreviations. SD:** Standard Deviation; **95% CI:** 95% Confidence Interval; **P:** Percentile.

# Supplementary table 2.3.5. Reference norms at 2-year follow-up for the Systemic Therapy Side Effects Scale of EORTC QLQ-BR23.

|  |  | **EORTC QLQ-BR23: Systemic Therapy Side Effects** | | | | |
| --- | --- | --- | --- | --- | --- | --- |
|  |  |  | **Charlson** | | **TNM stage** | |
|  |  | **All** | **0** | **≥ 1** | **0 – I** | **II - III** |
| **Age (years)**  **<40** | **n** | 33 | 31 | 2* | 14* | 19* |
|  | **Mean (SD)** | 12.0 (11.2) | 11.2 (11.1) | 23.8 (6.7) | 10.5 (9.4) | 13.0 (12.6) |
|  | **95%CI** | [ 8.1 - 15.8 ] | [ 7.3 - 15.1 ] | [ 14.5 - 33.1 ] | [ 5.6 - 15.5 ] | [ 7.4 - 18.7 ] |
|  | **P 5** | 0.0 | 0.0 |  |  |  |
|  | **P 10** | 0.0 | 0.0 |  |  |  |
|  | **P 20** | 0.0 | 0.0 |  |  |  |
|  | **P 25** | 0.0 | 0.0 |  |  |  |
|  | **P 30** | 1.0 | 0.0 |  |  |  |
|  | **P 40** | 4.8 | 4.8 |  |  |  |
|  | **Median** | 14.3 | 9.5 | 23.8 | 11.9 | 14.3 |
|  | **P 60** | 14.3 | 14.3 |  |  |  |
|  | **P 70** | 18.1 | 14.3 |  |  |  |
|  | **P 75** | 19.0 | 19.0 |  |  |  |
|  | **P 80** | 20.0 | 19.0 |  |  |  |
|  | **P 90** | 31.4 | 31.4 |  |  |  |
|  | **P 95** | 34.8 | 35.2 |  |  |  |
| **40-65** | **n** | 738 | 624 | 114 | 493 | 245 |
|  | **Mean (SD)** | 18.9 (16.9) | 18.5 (16.6) | 21.2 (18.4) | 18.1 (16.4) | 20.5 (17.8) |
|  | **95%CI** | [ 17.7 - 20.1 ] | [ 17.2 - 19.8 ] | [ 17.9 - 24.6 ] | [ 16.7 - 19.6 ] | [ 18.2 - 22.7 ] |
|  | **P 5** | 0.0 | 0.0 | 0.0 | 0.0 | 0.0 |
|  | **P 10** | 0.0 | 0.0 | 0.0 | 0.0 | 0.0 |
|  | **P 20** | 4.8 | 4.8 | 4.8 | 4.8 | 4.8 |
|  | **P 25** | 4.8 | 4.8 | 9.5 | 4.8 | 9.5 |
|  | **P 30** | 9.5 | 9.5 | 9.5 | 9.5 | 9.5 |
|  | **P 40** | 12.4 | 9.5 | 14.3 | 9.5 | 14.3 |
|  | **Median** | 14.3 | 14.3 | 14.3 | 14.3 | 14.3 |
|  | **P 60** | 19.0 | 19.0 | 19.0 | 19.0 | 19.0 |
|  | **P 70** | 23.8 | 23.8 | 23.8 | 23.8 | 28.6 |
|  | **P 75** | 28.6 | 28.6 | 28.6 | 23.8 | 28.6 |
|  | **P 80** | 28.6 | 28.6 | 33.3 | 28.6 | 33.3 |
|  | **P 90** | 42.9 | 41.4 | 50.0 | 38.1 | 47.6 |
|  | **P 95** | 52.4 | 52.4 | 58.3 | 52.4 | 57.1 |
| **>65** | **n** | 320 | 215 | 105 | 188 | 132 |
|  | **Mean (SD)** | 17.8 (16.3) | 16.6 (15.9) | 20.3 (16.9) | 16.9 (14.7) | 19.2 (18.2) |
|  | **95%CI** | [ 16.1 - 19.6 ] | [ 14.5 - 18.8 ] | [ 17.0 - 23.5 ] | [ 14.8 - 19.0 ] | [ 16.1 - 22.3 ] |
|  | **P 5** | 0.0 | 0.0 | 0.0 | 0.0 | 0.0 |
|  | **P 10** | 0.0 | 0.0 | 0.0 | 0.0 | 0.0 |
|  | **P 20** | 4.8 | 4.8 | 4.8 | 4.8 | 4.8 |
|  | **P 25** | 4.8 | 4.8 | 5.2 | 4.8 | 4.8 |
|  | **P 30** | 4.8 | 4.8 | 9.5 | 8.3 | 4.8 |
|  | **P 40** | 9.5 | 9.5 | 14.3 | 9.5 | 9.5 |
|  | **Median** | 14.3 | 14.3 | 19.0 | 14.3 | 14.3 |
|  | **P 60** | 19.0 | 15.7 | 21.0 | 19.0 | 19.0 |
|  | **P 70** | 23.8 | 23.8 | 28.6 | 23.8 | 24.3 |
|  | **P 75** | 28.6 | 23.8 | 28.6 | 23.8 | 33.3 |
|  | **P 80** | 28.6 | 28.6 | 33.3 | 28.6 | 33.3 |
|  | **P 90** | 38.1 | 38.1 | 44.8 | 33.8 | 47.6 |
|  | **P 95** | 52.4 | 48.6 | 52.4 | 47.6 | 54.0 |

* Deciles were not calculated for subgroups lower than 20 participants.

**Abbreviations. SD:** Standard Deviation; **95% CI:** 95% Confidence Interval; **P:** Percentile.

# Supplementary table 2.3.6. Reference norms at 2-year follow-up for the Breast Symptoms Scale of EORTC QLQ-BR23.

|  |  | **EORTC QLQ-BR23: Breast Symptoms Scale** | | | | |
| --- | --- | --- | --- | --- | --- | --- |
|  |  |  | **Charlson** | | **TNM stage** | |
|  |  | **All** | **0** | **≥ 1** | **0 – I** | **II - III** |
| **Age (years)**  **<40** | **n** | 34 | 32 | 2* | 14* | 20 |
|  | **Mean (SD)** | 17.4 (19.8) | 16.1 (19.5) | 37.5 (17.7) | 16.7 (16.0) | 17.9 (22.5) |
|  | **95%CI** | [ 10.7 - 24.1 ] | [ 9.4 - 22.9 ] | [ 13.0 - 62.0 ] | [ 8.3 - 25.1 ] | [ 8.1 - 27.8 ] |
|  | **P 5** | 0.0 | 0.0 |  |  | 0.0 |
|  | **P 10** | 0.0 | 0.0 |  |  | 0.0 |
|  | **P 20** | 0.0 | 0.0 |  |  | 0.0 |
|  | **P 25** | 0.0 | 0.0 |  |  | 0.0 |
|  | **P 30** | 0.0 | 0.0 |  |  | 0.0 |
|  | **P 40** | 8.3 | 8.3 |  |  | 8.3 |
|  | **Median** | 12.5 | 8.3 | 37.5 | 12.5 | 12.5 |
|  | **P 60** | 16.7 | 16.7 |  |  | 16.7 |
|  | **P 70** | 25.0 | 25.0 |  |  | 25.0 |
|  | **P 75** | 25.0 | 25.0 |  |  | 25.0 |
|  | **P 80** | 33.3 | 28.3 |  |  | 25.0 |
|  | **P 90** | 41.7 | 33.3 |  |  | 63.3 |
|  | **P 95** | 70.8 | 72.5 |  |  | 82.5 |
| **40-65** | **n** | 735 | 621 | 114 | 492 | 243 |
|  | **Mean (SD)** | 17.3 (18.0) | 17.3 (17.3) | 17.4 (21.1) | 16.7 (17.2) | 18.6 (19.4) |
|  | **95%CI** | [ 16.0 - 18.6 ] | [ 16.0 - 18.7 ] | [ 13.5 - 21.3 ] | [ 15.2 - 18.3 ] | [ 16.1 - 21.0 ] |
|  | **P 5** | 0.0 | 0.0 | 0.0 | 0.0 | 0.0 |
|  | **P 10** | 0.0 | 0.0 | 0.0 | 0.0 | 0.0 |
|  | **P 20** | 0.0 | 0.0 | 0.0 | 0.0 | 0.0 |
|  | **P 25** | 0.0 | 0.0 | 0.0 | 0.0 | 0.0 |
|  | **P 30** | 8.3 | 8.3 | 0.0 | 8.3 | 8.3 |
|  | **P 40** | 8.3 | 8.3 | 8.3 | 8.3 | 8.3 |
|  | **Median** | 16.7 | 16.7 | 8.3 | 16.7 | 16.7 |
|  | **P 60** | 16.7 | 16.7 | 16.7 | 16.7 | 16.7 |
|  | **P 70** | 25.0 | 25.0 | 25.0 | 25.0 | 25.0 |
|  | **P 75** | 25.0 | 25.0 | 25.0 | 25.0 | 25.0 |
|  | **P 80** | 25.0 | 25.0 | 33.3 | 25.0 | 33.3 |
|  | **P 90** | 41.7 | 41.7 | 50.0 | 41.7 | 50.0 |
|  | **P 95** | 50.0 | 50.0 | 66.7 | 50.0 | 58.3 |
| **>65** | **n** | 323 | 217 | 106 | 190 | 133 |
|  | **Mean (SD)** | 12.8 (17.0) | 11.7 (14.6) | 14.9 (20.9) | 13.2 (17.5) | 12.2 (16.2) |
|  | **95%CI** | [ 10.9 - 14.6 ] | [ 9.8 - 13.6 ] | [ 10.9 - 18.9 ] | [ 10.7 - 15.7 ] | [ 9.4 - 14.9 ] |
|  | **P 5** | 0.0 | 0.0 | 0.0 | 0.0 | 0.0 |
|  | **P 10** | 0.0 | 0.0 | 0.0 | 0.0 | 0.0 |
|  | **P 20** | 0.0 | 0.0 | 0.0 | 0.0 | 0.0 |
|  | **P 25** | 0.0 | 0.0 | 0.0 | 0.0 | 0.0 |
|  | **P 30** | 0.0 | 0.0 | 0.0 | 0.0 | 0.0 |
|  | **P 40** | 0.0 | 0.0 | 0.0 | 0.0 | 0.0 |
|  | **Median** | 8.3 | 8.3 | 8.3 | 8.3 | 8.3 |
|  | **P 60** | 8.3 | 8.3 | 8.3 | 8.3 | 8.3 |
|  | **P 70** | 16.7 | 16.7 | 16.7 | 16.7 | 16.7 |
|  | **P 75** | 16.7 | 16.7 | 25.0 | 16.7 | 16.7 |
|  | **P 80** | 25.0 | 16.7 | 33.3 | 25.0 | 25.0 |
|  | **P 90** | 33.3 | 33.3 | 44.2 | 33.3 | 38.3 |
|  | **P 95** | 50.0 | 41.7 | 66.7 | 50.0 | 50.0 |

* Deciles were not calculated for subgroups lower than 20 participants.

**Abbreviations. SD:** Standard Deviation; **95% CI:** 95% Confidence Interval; **P:** Percentile.

# Supplementary table 2.3.7. Reference norms at 2-year follow-up for the Arm Symptoms Scale of EORTC QLQ-BR23.

|  |  | **EORTC QLQ-BR23: Arm Symptoms Scale** | | | | |
| --- | --- | --- | --- | --- | --- | --- |
|  |  |  | **Charlson** | | **TNM stage** | |
|  |  | **All** | **0** | **≥ 1** | **0 – I** | **II - III** |
| **Age (years)**  **<40** | **n** | 34 | 32 | 2* | 14* | 20 |
|  | **Mean (SD)** | 9.5 (15.3) | 9.0 (15.6) | 16.7 (7.9) | 2.4 (4.7) | 14.4 (18.1) |
|  | **95%CI** | [ 4.3 - 14.6 ] | [ 3.6 - 14.4 ] | [ 5.8 - 27.6 ] | [ -0.1 - 4.9 ] | [ 6.5 - 22.4 ] |
|  | **P 5** | 0.0 | 0.0 |  |  | 0.0 |
|  | **P 10** | 0.0 | 0.0 |  |  | 0.0 |
|  | **P 20** | 0.0 | 0.0 |  |  | 0.0 |
|  | **P 25** | 0.0 | 0.0 |  |  | 0.0 |
|  | **P 30** | 0.0 | 0.0 |  |  | 0.0 |
|  | **P 40** | 0.0 | 0.0 |  |  | 0.0 |
|  | **Median** | 0.0 | 0.0 | 16.7 | 0.0 | 11.1 |
|  | **P 60** | 11.1 | 0.0 |  |  | 11.1 |
|  | **P 70** | 11.1 | 11.1 |  |  | 22.2 |
|  | **P 75** | 11.1 | 11.1 |  |  | 22.2 |
|  | **P 80** | 22.2 | 15.6 |  |  | 31.1 |
|  | **P 90** | 33.3 | 33.3 |  |  | 53.3 |
|  | **P 95** | 55.6 | 55.6 |  |  | 55.6 |
| **40-65** | **n** | 735 | 621 | 114 | 492 | 243 |
|  | **Mean (SD)** | 16.0 (20.1) | 15.7 (19.9) | 17.6 (20.7) | 13.9 (18.7) | 20.3 (21.9) |
|  | **95%CI** | [ 14.6 - 17.5 ] | [ 14.2 - 17.3 ] | [ 13.8 - 21.4 ] | [ 12.3 - 15.6 ] | [ 17.5 - 23.1 ] |
|  | **P 5** | 0.0 | 0.0 | 0.0 | 0.0 | 0.0 |
|  | **P 10** | 0.0 | 0.0 | 0.0 | 0.0 | 0.0 |
|  | **P 20** | 0.0 | 0.0 | 0.0 | 0.0 | 0.0 |
|  | **P 25** | 0.0 | 0.0 | 0.0 | 0.0 | 0.0 |
|  | **P 30** | 0.0 | 0.0 | 0.0 | 0.0 | 0.0 |
|  | **P 40** | 0.0 | 0.0 | 11.1 | 0.0 | 11.1 |
|  | **Median** | 11.1 | 11.1 | 11.1 | 11.1 | 11.1 |
|  | **P 60** | 11.1 | 11.1 | 11.1 | 11.1 | 22.2 |
|  | **P 70** | 22.2 | 22.2 | 22.2 | 22.2 | 22.2 |
|  | **P 75** | 22.2 | 22.2 | 33.3 | 22.2 | 33.3 |
|  | **P 80** | 33.3 | 28.9 | 33.3 | 22.2 | 33.3 |
|  | **P 90** | 44.4 | 44.4 | 44.4 | 33.3 | 55.6 |
|  | **P 95** | 55.6 | 55.6 | 66.7 | 55.6 | 66.7 |
| **>65** | **n** | 321 | 215 | 106 | 190 | 131 |
|  | **Mean (SD)** | 15.3 (20.8) | 13.8 (18.4) | 18.2 (24.7) | 14.0 (19.9) | 17.1 (22.0) |
|  | **95%CI** | [ 13.0 - 17.5 ] | [ 11.4 - 16.3 ] | [ 13.5 - 22.9 ] | [ 11.2 - 16.8 ] | [ 13.4 - 20.9 ] |
|  | **P 5** | 0.0 | 0.0 | 0.0 | 0.0 | 0.0 |
|  | **P 10** | 0.0 | 0.0 | 0.0 | 0.0 | 0.0 |
|  | **P 20** | 0.0 | 0.0 | 0.0 | 0.0 | 0.0 |
|  | **P 25** | 0.0 | 0.0 | 0.0 | 0.0 | 0.0 |
|  | **P 30** | 0.0 | 0.0 | 0.0 | 0.0 | 0.0 |
|  | **P 40** | 0.0 | 0.0 | 0.0 | 0.0 | 0.0 |
|  | **Median** | 11.1 | 11.1 | 11.1 | 0.0 | 11.1 |
|  | **P 60** | 11.1 | 11.1 | 11.1 | 11.1 | 11.1 |
|  | **P 70** | 22.2 | 22.2 | 22.2 | 22.2 | 22.2 |
|  | **P 75** | 22.2 | 22.2 | 22.2 | 22.2 | 22.2 |
|  | **P 80** | 33.3 | 22.2 | 33.3 | 22.2 | 33.3 |
|  | **P 90** | 44.4 | 44.4 | 58.9 | 44.4 | 44.4 |
|  | **P 95** | 66.7 | 45.6 | 73.9 | 52.5 | 66.7 |

* Deciles were not calculated for subgroups lower than 20 participants.

**Abbreviations. SD:** Standard Deviation; **95% CI:** 95% Confidence Interval; **P:** Percentile.

# Supplementary table 2.3.8. Reference norms at 2-year follow-up for the Upset by Hair Loss Scale of EORTC QLQ-BR23.

|  |  | **EORTC QLQ-BR23: Upset by Hair Loss Scale** | | | | |
| --- | --- | --- | --- | --- | --- | --- |
|  |  |  | **Charlson** | | **TNM stage** | |
|  |  | **All** | **0** | **≥ 1** | **0 – I** | **II - III** |
| **Age (years)**  **<40** | **n** | 11* | 9* | 2* | 4* | 7* |
|  | **Mean (SD)** | 12.1 (30.8) | 11.1 (33.3) | 16.7 (23.6) | 8.3 (16.7) | 14.3 (37.8) |
|  | **95%CI** | [ -6.1 - 30.3 ] | [ -10.7 - 32.9 ] | [ -16.0 - 49.3 ] | [ -8.0 - 24.7 ] | [ -13.7 - 42.3 ] |
|  | **P 5** |  |  |  |  |  |
|  | **P 10** |  |  |  |  |  |
|  | **P 20** |  |  |  |  |  |
|  | **P 25** |  |  |  |  |  |
|  | **P 30** |  |  |  |  |  |
|  | **P 40** |  |  |  |  |  |
|  | **Median** | 0.0 | 0.0 | 16.7 | 0.0 | 0.0 |
|  | **P 60** |  |  |  |  |  |
|  | **P 70** |  |  |  |  |  |
|  | **P 75** |  |  |  |  |  |
|  | **P 80** |  |  |  |  |  |
|  | **P 90** |  |  |  |  |  |
|  | **P 95** |  |  |  |  |  |
| **40-65** | **n** | 241 | 204 | 37 | 159 | 82 |
|  | **Mean (SD)** | 32.4 (33.5) | 30.2 (32.2) | 44.1 (38.5) | 30.0 (33.8) | 37.0 (32.7) |
|  | **95%CI** | [ 28.1 - 36.6 ] | [ 25.8 - 34.6 ] | [ 31.7 - 56.6 ] | [ 24.7 - 35.2 ] | [ 29.9 - 44.1 ] |
|  | **P 5** | 0.0 | 0.0 | 0.0 | 0.0 | 0.0 |
|  | **P 10** | 0.0 | 0.0 | 0.0 | 0.0 | 0.0 |
|  | **P 20** | 0.0 | 0.0 | 0.0 | 0.0 | 0.0 |
|  | **P 25** | 0.0 | 0.0 | 0.0 | 0.0 | 0.0 |
|  | **P 30** | 0.0 | 0.0 | 13.3 | 0.0 | 30.0 |
|  | **P 40** | 26.7 | 0.0 | 33.3 | 0.0 | 33.3 |
|  | **Median** | 33.3 | 33.3 | 33.3 | 33.3 | 33.3 |
|  | **P 60** | 33.3 | 33.3 | 33.3 | 33.3 | 33.3 |
|  | **P 70** | 33.3 | 33.3 | 66.7 | 33.3 | 33.3 |
|  | **P 75** | 50.0 | 33.3 | 83.3 | 33.3 | 66.7 |
|  | **P 80** | 66.7 | 66.7 | 100.0 | 66.7 | 66.7 |
|  | **P 90** | 100.0 | 83.3 | 100.0 | 100.0 | 100.0 |
|  | **P 95** | 100.0 | 100.0 | 100.0 | 100.0 | 100.0 |
| **>65** | **n** | 147 | 95 | 52 | 91 | 56 |
|  | **Mean (SD)** | 31.1 (34.8) | 30.2 (34.4) | 32.7 (35.8) | 27.5 (31.7) | 36.9 (39.0) |
|  | **95%CI** | [ 25.4 - 36.7 ] | [ 23.3 - 37.1 ] | [ 22.9 - 42.4 ] | [ 21.0 - 34.0 ] | [ 26.7 - 47.1 ] |
|  | **P 5** | 0.0 | 0.0 | 0.0 | 0.0 | 0.0 |
|  | **P 10** | 0.0 | 0.0 | 0.0 | 0.0 | 0.0 |
|  | **P 20** | 0.0 | 0.0 | 0.0 | 0.0 | 0.0 |
|  | **P 25** | 0.0 | 0.0 | 0.0 | 0.0 | 0.0 |
|  | **P 30** | 0.0 | 0.0 | 0.0 | 0.0 | 0.0 |
|  | **P 40** | 0.0 | 0.0 | 0.0 | 0.0 | 0.0 |
|  | **Median** | 33.3 | 33.3 | 33.3 | 33.3 | 33.3 |
|  | **P 60** | 33.3 | 33.3 | 33.3 | 33.3 | 33.3 |
|  | **P 70** | 33.3 | 40.0 | 33.3 | 33.3 | 66.7 |
|  | **P 75** | 66.7 | 66.7 | 58.3 | 33.3 | 66.7 |
|  | **P 80** | 66.7 | 66.7 | 66.7 | 66.7 | 86.7 |
|  | **P 90** | 100.0 | 80.0 | 100.0 | 66.7 | 100.0 |
|  | **P 95** | 100.0 | 100.0 | 100.0 | 100.0 | 100.0 |

* Deciles were not calculated for subgroups lower than 20 participants.

**Abbreviations. SD:** Standard Deviation; **95% CI:** 95% Confidence Interval; **P:** Percentile.
